# Interfacial electronic structure engineering on molybdenum sulfide for robust dual-pH hydrogen evolution

**DOI:** 10.1038/s41467-021-25647-8

**Published:** 2021-09-06

**Authors:** Mingqiang Liu, Jia-Ao Wang, Wantana Klysubun, Gui-Gen Wang, Suchinda Sattayaporn, Fei Li, Ya-Wei Cai, Fuchun Zhang, Jie Yu, Ya Yang

**Affiliations:** 1 Shenzhen Key Laboratory for Advanced Materials, School of Materials Science and Engineering, Harbin Institute of Technology, Shenzhen, People’s Republic of China; 2 CAS Center for Excellence in Nanoscience, Beijing Key laboratory of Micro-nano Energy and Sensor, Beijing Institute of Nanoenergy and Nanosystems, Chinese Academy of Science, Beijing, People’s Republic of China; 3 Department of Chemistry and the Oden Institute for Computational Engineering and Sciences, University of Texas at Austin, Austin, TX USA; 4 Synchrotron Light Research Institute, Muang, Nakhon Ratchasima, Thailand; 5 School of Physics and Electronic Information, Yan’an University, Yan’an, People’s Republic of China; 6 School of Nanoscience and Technology, University of Chinese Academy of Sciences, Beijing, People’s Republic of China

**Keywords:** Electrocatalysis, Nanoscale materials

## Abstract

Molybdenum disulfide, as an electronic highly-adjustable catalysts material, tuning its electronic structure is crucial to enhance its intrinsic hydrogen evolution reaction (HER) activity. Nevertheless, there are yet huge challenges to the understanding and regulation of the surface electronic structure of molybdenum disulfide-based catalysts. Here we address these challenges by tuning its electronic structure of phase modulation synergistic with interfacial chemistry and defects from phosphorus or sulfur implantation, and we then successfully design and synthesize electrocatalysts with the multi-heterojunction interfaces (e.g., 1T_0.81_-MoS_2_@Ni_2_P), demonstrating superior HER activities and good stabilities with a small overpotentials of 38.9 and 95 mV at 10 mA/cm^2^, a low Tafel slopes of 41 and 42 mV/dec in acidic as well as alkaline surroundings, outperforming commercial Pt/C catalyst and other reported Mo-based catalysts. Theoretical calculation verified that the incorporation of metallic-phase and intrinsic HER-active Ni-based materials into molybdenum disulfide could effectively regulate its electronic structure for making the bandgap narrower. Additionally, X-ray absorption spectroscopy indicate that reduced nickel possesses empty orbitals, which is helpful for additional H binding ability. All these factors can decrease Mo-H bond strength, greatly improving the HER catalytic activity of these materials.

## Abstract (web summary)

The understanding and regulation of the surface electronic structure of molybdenum disulfide-based catalysts for hydrogen evolution reaction (HER) remains a challenges. Here, the authors design and synthesize electrocatalysts with multi-heterojunction interfaces showing enhanced HER activities and stabilities.

## Introduction

Extensive use and depletion of fossil fuels resulting in serious pollution. Therefore, green and renewable fuel resources are required for continuing sustainable economic development^1–3^. Electrocatalysis acts as a vital role in the conversion of clean energy to achieve a sustainable approach to various commercial processes, including HER^4,5^. However, electrochemical water splitting is hindered by the large kinetic barrier and slow kinetics^6–9^. Pt-based electrocatalysts are recognized as highly efficient electrocatalysts due to good electrical conductivity^10^, fast kinetics^11,12^, and the preference to overcome the large kinetic energy barrier involved in the above-mentioned process^13^. Unfortunately, high price and not desirable stability hinder the extended Pt-based catalysts’ application^14^. Thus, it is very urgent to develop cost-effective Pt-free electrocatalysts with comparable activity and better stability.

Researchers recently have designed a wide range of low-cost catalysts, including transition-metal chalcogenides (TMDCs)^15,16^, metal nitrides^17,18^, metal carbides^19,20^, and metal phosphides^21,22^. Among these candidates, MoS_2_, a typical layered 2D TMDCs formed by Van der Waals interaction and stacking of S–Mo–S layers, attracts extensive interests with its adjustable bandgap, unique band structure, high energy-conversion efficiency, and earth abundance^23–25^. However, the electrocatalytic activity of MoS_2_ is closely associated with its surface electric structure^26–36^, many researchers have focused on adjusting the electronic structure of the MoS_2_ surface to promote electrocatalytic activity, such as surface engineering^26^, doping^27^, single-atom anchoring^28^, phase structure^29–33^, interface active site^34,35^, and defect^36^. Interestingly, two main phases of MoS_2_ were widely justified: 2H and 1T phases^29^. 2H phase has the most thermodynamical stability among the molybdenum sulfide family, whose HER activities are restrained by the amount and active site types as well as conductivity. Unlike the 2H phase, 1T-phase one demonstrates higher catalytic activity since it has numerous active sites on the edges and a fast transfer rate. However, it is remaining a giant challenge of directly synthesizing the high percentage 1T-phase molybdenum sulfide due to the thermodynamic instability of 1T_phase_-MoS_2_^30^. To solve this problem, a feasible strategy is to efficiently realize the 2H → 1T-phase transformation to improve HER capability. Wang et al. found that a partial 2H → 1T-MoS_2_ phase transition by facile one-pot annealing of a large amount of 2H_phase_-MoS_2_ under phosphorus vapor is able to enhance HER catalytic activities^31^. A synergistic strategy of doping nitrogen and intercalating PO_4_^3−^ is reported, which can convert 2H- to 1T-phase with a conversion rate of up to 41%, and has excellent HER performance^32^. However, the electronic transport capacity and phase stability of the phase boundary of a single component (pure 1T-phase) are generally poor. In order to overcome the puzzles, the HER activity of the pure phase can be improved by constructing a heterogeneous boundary. Therefore, it is expected to further enhance the HER performance and its stability of traditional single 1T-phase or 2H-phase interface by constructing a composite heterojunction between 1T-phase and the other phases^33^.

Interface modification could be an effective approach to construct a composite heterojunction^34,35^. Ni-based materials (such as Ni_2_P, NiS_2_, Ni_2_S_3,_ etc) with high activity and conductivity have been considered as highly efficient electrocatalysis materials for HER^21,22,37^, as another heterogeneous interface, which is also very important to control the electronic structure of the MoS_2_ interface. Kim et al. reported that Ni_2_P nanoparticles were used to activate the MoS_2_ base surface, which exhibits Pt-like HER performance in 0.5 M HCl solution^37^. Because the electronic structure of Ni_2_P is a $P\bar{6}2m$ space group, which could facilitate recombination at the atomic scale. Moreover, Ni has a unique α and β orbital integral asymmetric *d* orbital, which makes it easy for the lone pair of electrons to recombine with the *d* orbital of the exposed Mo atom on MoS_2_ to generate new interface electrons, thereby improving HER performance. Lin et al. reported that a defect-rich heterogeneous interfacial catalyst (MoS_2_/NiS_2_) could provide abundant active sites to promote electron transfer, thereby further rapidly promoting electrocatalytic hydrogen evolution^38^. More importantly, the introduction of NiS_2_ hybridization on the surface of MoS_2_ generates a new form of interface electrons, and Ni^δ+^ is reduced to low-valence Ni to improve the binding energy with hydrogen elements, thereby weakening the Mo–H strength. To sum up, although the heterojunction-phase catalyst synthesized by the above-mentioned approach further improves the HER activity and good stability, the understanding and regulation of the surface electronic structure on the MoS_2_ interface are still huge challenges, and thus it is very necessary to develop an efficient synthesis approach to obtain stable multi-heterogeneous interface catalyst.

**Fig. 1:**
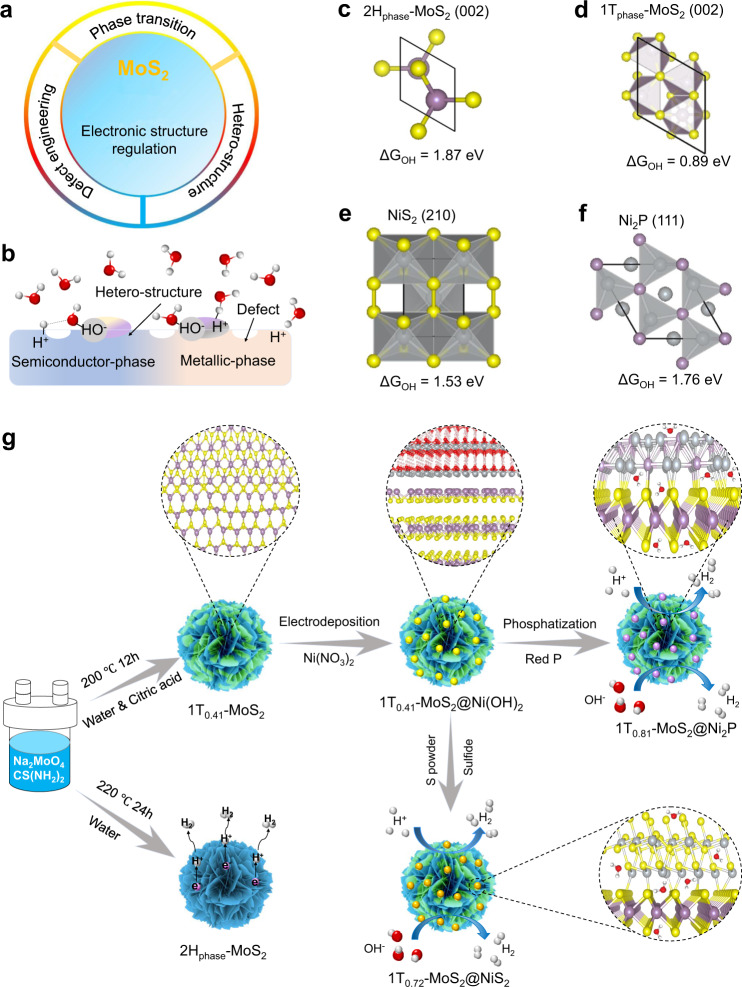
Motivation and design of electrocatalyst. **a** Tuning strategy of the electronic structure of the MoS_2_ surface. **b** Design ideas of hydrogen evolution catalyst. **c**–**f** the energetics of hydroxyl species on 2H_phase_-MoS_2_ (002), 1T_phase_-MoS_2_ (002), NiS_2_ (210), and Ni_2_P (111) HER electrocatalyst surfaces. **g** Schematics of the 1T_0.72_-MoS_2_@NiS_2_ and 1T_0.81_-MoS_2_@Ni_2_P synthesis steps.

Here, we address these challenges by tuning its electronic structure through phase modulation synergistic with interfacial chemistry and defects of phosphorus or sulfur implantation, and we then successfully design and prepare a series of heterojunction-phase-interface electrocatalysts (denoted 1T_0.81_-MoS_2_@Ni_2_P and 1T_0.72_-MoS_2_@NiS_2_) with an outstanding HER activity and are stable in dual-pH surroundings. The strategies to control the electronic characteristics of the MoS_2_ surface include surface phase modulation, surface defects, and the construction of hetero-structure (Fig. 1a). Furthermore, we control the hydrogen and hydroxyl adsorption energy through the synergistic effect of heterojunction-phase-interface catalysts (Fig. 1b, c–f) because the energy of the hydroxyl species is very important for the hydrolysis accelerator. Starting from hydrothermally synthesized MoS_2_ nanosheets, we develop a simple surface electronic structure modulation strategy of constructing multi-heterogeneous-phase-interface 1T_0.81_-MoS_2_@Ni_2_P and 1T_0.72_-MoS_2_@NiS_2_ electrocatalysts (Fig. 1a) by citric acid-induced hydrothermal synthesis, electrodeposition and then phosphorus (or sulfur) vapor thermal treatment approach for the first time. Our approach can not only realize the construction of abundant catalytic reactive sites but also improve the conversion rate of 2H to 1T (81%), and it is also convenient to introduce Ni_2_P or NiS_2_ heterogeneous interfaces. As to the surface electronic structure of catalysts, high-resolution transmission electron microscopy (HRTEM) images show that such phase-structures, heterojunction-phase-interface edges, and defects are derived by the featured electronic states and Ni atomic coordination. Additionally, X-ray photoelectron spectra (XPS) showed that citric acid induces hydrothermal synthesis of stable 1T_0.41_-MoS_2_ (41% of 1T-phase), and the 1T_0.81_-MoS_2_ or 1T_0.72_-MoS_2_ (81% or 72% of 1T-phase) conversion rate is further improved after phosphorus or sulfur vapor thermal treatment. As-synthesized 1T_0.81_-MoS_2_@Ni_2_P (or 1T_0.72_-MoS_2_@NiS_2_) multi-heterogeneous catalyst exhibits the remarkable HER catalytic activity, achieving the low overpotentials of 38.9 (or 186) and 98.5 mV (or 128 mV) for HER at a current density of 10 mA/cm^2^. They also have Tafel slopes of 41 (or 79) and 42 (or 68) mV/dec in 0.5 M H_2_SO_4_ or 1.0 M KOH media, and good stability during testing for 16 h in both media, respectively. The 1T_0.81_-MoS_2_@Ni_2_P (or 1T_0.72_-MoS_2_@NiS_2_) catalysts exhibited superior activities with Tafel slope values and the overpotentials lower than the values reported for Mo-base HER catalysts in both alkaline and acidic media^30,31,33,37–40^. Moreover, as-synthesized 1T_0.72_-MoS_2_@NiS_2_ (or 1T_0.81_-MoS_2_@Ni_2_P) catalyst also exhibits excellent OER and overall-water splitting catalytic activity. DFT calculation results display that the introduction of 1T-phase MoS_2_ and Ni-based materials can regulate MoS_2_ electronic structure effectively for making the bandgap narrower, and decreasing H* and water adsorption energy. In situ electrochemical-Raman spectra results indicate that the OH– ions are driven to be adsorbed on Mo, Ni atoms in the alkaline medium, and then there forms the OOH* intermediates. There is a strong interaction between Ni and Mo on the surface of the catalyst, thereby increasing the local electronic state of Mo atoms, reducing the hydrogen-adsorption energy for protons on Mo atoms, and thus improving its intrinsic catalytic. Moreover, X-ray absorption spectroscopy results imply that reduced Ni supply empty *d*-orbitals to facilitate H atom capture, and decrease Mo–H strength of 1T_0.81_-MoS_2_@Ni_2_P (or 1T_0.72_-MoS_2_@NiS_2_) catalyst. This work provides useful insights for exploring the enhancement mechanisms of HER with an optimized surface electronic structure on the MoS_2_ interface, which provides an effective insight of constructing invaluable metal electrocatalysts for HER and other fields.

## Results

### Preparation and characterizations of multi-heterojunction interface electrocatalysts

The formation process of multi-heterojunction interface electrocatalysts is schematically illustrated in Fig. 1g. 1T_0.81_-MoS_2_@Ni_2_P and 1T_0.72_-MoS_2_@NiS_2_ catalysts were synthesized by a three-step procedure. First, 1T_0.41_-MoS_2_ nanospheres were obtained on carbon cloth (CC) by acid-induced hydrothermal approach at 200 °C for 12 h (see details in “Methods” section). The as-obtained 1T_0.41_-MoS_2_ shows a large number of microspheres (Supplementary Fig. 1b–d) with a narrow diameter distribution of 2.0–4.0 µm distributed uniformly on the surface of CC substrate. Flower-shaped MoS_2_ microspheres consist of many aligned 1T_0.41_-MoS_2_ nanosheets, on which the Ni(OH)_2_ nanoparticles were then electrodeposited (see details in “Methods” section). 1T_0.41_-MoS_2_@Ni(OH)_2_ material inherited its morphology from spherical MoS_2_. When being electrodeposited for 100 s, a small amount of Ni(OH)_2_ nanoparticles can be anchored on the surface of MoS_2_ nanospheres (Supplementary Fig. 2). As the electrodeposition time increases to 300 s, a large number of Ni(OH)_2_ nanoparticles can be observed to adhere to the MoS_2_ surface (Supplementary Fig. 3). Subsequently, as-prepared 1T_0.41_-MoS_2_@Ni(OH)_2_ was loaded into a quartz tube mixed with red phosphorus or sulfur powder and sealed by oxyacetylene flame. Finally, these were heated to 600 °C for the reaction with red phosphorus or sulfur to synthesize 1T_0.81_-MoS_2_@Ni_2_P and 1T_0.72_-MoS_2_@NiS_2_ catalysts, respectively (Supplementary Figs. 4 and 5). As to 1T_0.81_-MoS_2_@Ni_2_P catalyst, the MoS_2_ microspheres are very rough, on which there distribute many random Ni_2_P nanoparticles (Supplementary Fig. 5). It is because that the 1T/2H-mixed phase and heterojunction-interface structure reduces the adhesion of the gas-solid interface and facilitates releasing hydrogen from the catalyst surface, which is essential for enhancing HER^34^.

Next, the phase composition and crystal structure of 1T_0.81_-MoS_2_@Ni_2_P and 1T_0.72_-MoS_2_@NiS_2_ were obtained by X-ray diffraction (XRD) and Raman spectroscopy. There are some obvious characteristic diffraction peaks of 14.3°, 33.4°, and 59.2° (Supplementary Fig. 6b, c), which can be ascribed to 2H_phase_-MoS_2_ (JCPDS#75-1539). However, the XRD peak of 1T_0.81_-MoS_2_@Ni_2_P and 1T_0.72_-MoS_2_@NiS_2_ located at 2θ ≈ 28.8° can be indexed as the (004) peak of 1T_phase_-MoS_2_, which indicates that 1T- and 2H-mixed phases were successfully hydrothermally synthesized^41^. The other characteristic peaks (2*θ* ≈ 31.3°, 35.2°, 38.8°, 44.9°, and 53.3°) demonstrate that the 1T_0.72_-MoS_2_@NiS_2_ is a hybrid of NiS_2_ (JCPDS#11-0099), which verifies the presence of NiS_2_ nanoparticles. Similarly, as to 1T_0.81_-MoS_2_@Ni_2_P catalyst, its XRD results also showed the presence of Ni_2_P nanoparticles (JCPDS#21-0590) on the 1T_0.41_-MoS_2_ surface. Raman spectroscopy showed *E*_2g_^1^ and *A*_1g_ vibrational bands at 376.2 and 402.9 cm^−1^ peaks typical for 2H_phase_-MoS_2_^42^_._ *J*_1_, *J*_2_, and *J*_3_ vibrations at 147.3, 235.4 and 335.2 cm^−1^ are characteristic for 1T_phase_-MoS_2_^43^ (Supplementary Fig. 6b). These results prove that the 1T-phase of MoS_2_ is formed by the hydrothermal reaction induced by organic acids (e.g., citric acid)^41^. 1T_0.72_-MoS_2_@NiS_2_ or 1T_0.81_-MoS_2_@Ni_2_P demonstrated three characteristic peaks of 1T_phase_-MoS_2_ and the two characteristic peaks (*E*_2g_^1^ and *A*_1g_) of 2H_phase_-MoS_2_. Additionally, they showed a vibrational peak (437.3 cm^−1^) of Ni–S^38^ or three vibrational peaks (216.2, 249.7, and 269.5 cm^−1^) of Ni–P^37^. More importantly, the *E*_2g_^1^ and *A*_1g_ vibrations of 1T_0.72_-MoS_2_@NiS_2_ at 382.2 and 408.1 cm^−1^ were red-shifted by 6.0 and 5.2 cm^−1^, respectively (Supplementary Fig. 6d). This could be attributed to the exploits the S layer of MoS_2_ as an external S source to grow NiS_2_ in situ. Therefore, it changes the original vibration mode of the Mo–S bonds, and the out-of-plane vibration mode has a more significant change^44–46^. Similarly, the *E*_2g_^1^ and *A*_1g_ peaks for the 1T_0.81_-MoS_2_@Ni_2_P catalyst slightly red-shifted by 7.3 and 3.0 cm^−1^, respectively, because of interfacial stress between Ni_2_P and MoS_2_, indicating that the formation of MoS_2_@Ni_2_P heterojunction leads to the Raman shift of MoS_2_^44–46^. These results confirm that rich multi-heterojunction interface edges active sites catalysts were successfully synthesized.

### Electronic structure characterizations of 1T_0.72_-MoS_2_@NiS_2_ and 1T_0.81_-MoS_2_@Ni_2_P catalysts

**Fig. 2:**
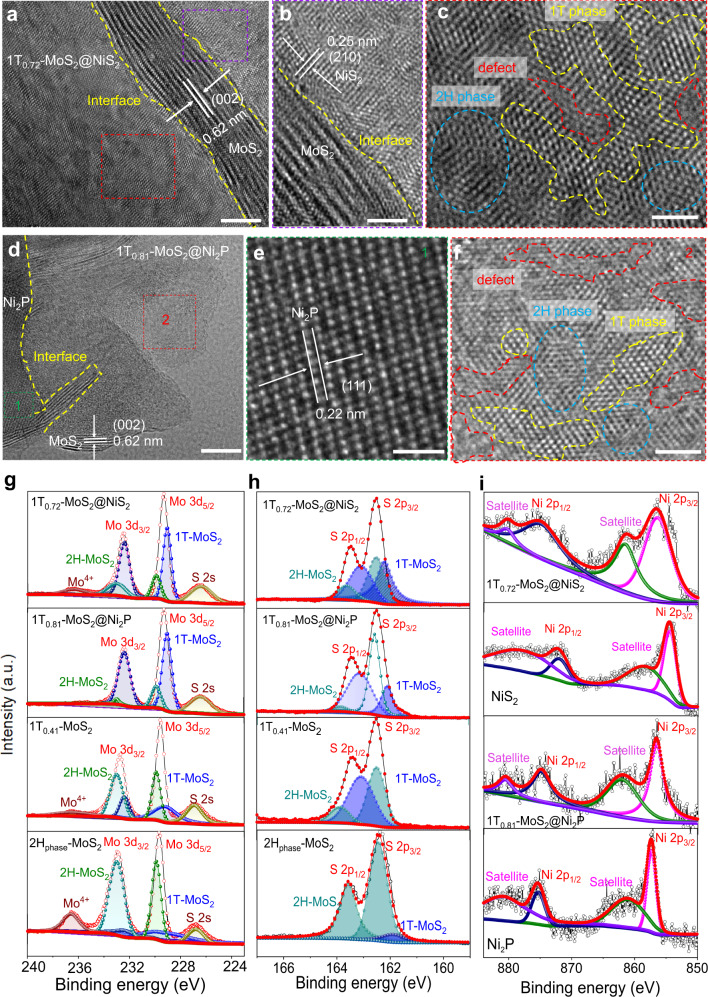
Electronic structure characterizations of 1T_0.72_-MoS_2_@NiS_2_ and 1T_0.81_-MoS_2_@Ni_2_P catalysts. **a**–**c** HRTEM image of 1T_0.72_-MoS_2_@NiS_2_. **a** MoS_2_ lattice, scale bars are 5 nm. **b** NiS_2_ lattice and heterojunction interface, scale bars are 1 nm. **c** shows 2H and 1T lattices, scale bars are 2 nm. **d**–**f** Typical HRTEM image of 1T_0.81_-MoS_2_@Ni_2_P. **d** shows MoS_2_ lattice and heterojunction interface, scale bars are 5 nm. **e** shows Ni_2_P lattice fringes, scale bars are 1 nm. **f** shows 2H and 1T lattices, scale bars are 2 nm. **g** HR Mo 3*d* core-level XPS spectra of 1T_0.72_-MoS_2_@NiS_2_, 1T_0.81_-MoS_2_@Ni_2_P, 1T_0.41_-MoS_2_, and 2H_phase_-MoS_2_. **h** S 2*p* core-level XPS spectra of 1T_0.72_-MoS_2_@NiS_2_, 1T_0.81_-MoS_2_@Ni_2_P, 1T_0.41_-MoS_2_ and 2H_phase_-MoS_2_, respectively. **i** Ni 2*p* XPS spectrum for 1T_0.72_-MoS_2_@NiS_2_, 1T_0.81_-MoS_2_@Ni_2_P, pure Ni_2_P, and Ni_2_S.

To further identify the surface electronic structure of multi-heterogeneous interface catalysts, we applied the high-resolution transmission electron microscopy (HRTEM) to assess the morphology and crystal structures of 1T_0.81_-MoS_2_@Ni_2_P and 1T_0.72_-MoS_2_@NiS_2_ catalysts. Supplementary Fig. 7a, b shows the typical low-magnification image of the 1T_0.72_-MoS_2_@NiS_2_ on the Cu grid, which confirms the flower-like nanosphere morphologies of 1T_0.72_-MoS_2_@NiS_2_. TEM and corresponding elemental distribution map obtained for the 1T_0.72_-MoS_2_@NiS_2_ sample demonstrated uniformly distributed Mo, Ni, and S (Supplementary Fig. 7c-c4). As revealed by the HRTEM image (Fig. 2a–c and Supplementary Fig. 7e, f), NiS_2_ nanoparticles are decorated on MoS_2_ nanosheets edge (Supplementary Fig. 10a, b). The HRTEM image of 1T_0.72_-MoS_2_@NiS_2_clearly shows the crystal lattice of 0.25 nm, referring to the NiS_2_ (210). Interestingly, Fig. 2a shows the HRTEM image of 1T_0.72_-MoS_2_@NiS_2_ flower-like nanosheets, which there demonstrate the lattice fringes perpendicularly to the electron beam direction circled by blood color, justifying the S defect (Fig. 2c). The trigonal lattice in the yellow circle implies the presence of 1T-phase MoS_2_, while the hexagonal lattice in the blue circle suggests the presence of 2H phase MoS_2_. The above-described results further confirm the successful preparation of the 1T_0.72_-MoS_2_@NiS_2_ multi-heterojunction interface catalyst. The anion is changed to be P to produce 1T_0.81_-MoS_2_@Ni_2_P multi-heterojunction interface catalyst by phosphorus vapor thermal treatment. Supplementary Fig. 8a, b displays the morphologies of 1T_0.81_-MoS_2_@Ni_2_P catalyst, overlapping nanosheets with many embedded particles can be clearly identified. There is an obvious alternation of 1T and 2H phases, and a large number of defects or disorder (Fig. 2f and Supplementary Fig. 9). As shown in Supplementary Fig. 8c, there are the distributions of Mo, Ni, S, and P over the whole 1T_0.81_-MoS_2_@Ni_2_P, verifying that Ni_2_P nanoparticles are encapsulated by MoS_2_ edges (Supplementary Fig. 10c, d). The interplanar spacings of 0.62 and 0.22 nm are assigned to (002) and (111) interplanar distances of MoS_2_ and Ni_2_P, respectively (Fig. 2d, e). Similarly, Fig. 2e, f displays two amplified HRTEM images truncated from Fig. 2d, in which Fig. 2f demonstrates some hexagonal and trigonal lattice areas of semiconductor 2H_phase_- and metallic 1T_phase_-MoS_2,_ respectively. The HRTEM results further confirm the successful preparation of the 1T_0.81_-MoS_2_@Ni_2_P multi-heterojunction interface catalyst.

Next, we performed XPS measurement to assess the elemental valence states of all the as-synthesized samples (Fig. 2g–i and Supplementary Fig. 13a). Full XPS spectrum for 1T_0.72_-MoS_2_@NiS_2_ (Supplementary Fig. 13a) showed that atomic ratios of Mo, S, and Ni were equal to 13.96%, 36.96%, and 4.39%, respectively, and close to that measured by HRTEM elemental mapping (~14.30%, 35.87%, and 4.76%). Mo 3*d* spectra obtained for the 1T_0.41_-MoS_2_ sample shows Mo^4+^ 3*d*_3/2_ and Mo^4+^ 3*d*_5/2_ peaks at 232.68 and 229.43 eV (Fig. 2g), respectively, confirming the existence of Mo^4+^ for the 1T_0.41_-MoS_2_. As to the 1T_0.72_-MoS_2_@NiS_2_, or 1T_0.81_-MoS_2_@Ni_2_P heterostructures catalyst, the high-solution Mo 3*d* XPS spectrum shows that both Mo^4+^ 3*d*_3/2_ and Mo^4+^ 3*d*_5/2_ peaks for mixed-phase MoS_2_ has a shift of 0.23 eV and 0.15 eV to lower binding energy compared with 1T_0.41_-MoS_2_ (Supplementary Fig. 11a), which is attributed to the existence of 1T_phase_-MoS_2_^47^. In addition, two peaks of 163.41 and 162.22 eV are observed in the 1T_0.41_-MoS_2_, corresponding to S^2−^ 2*p*_1/2_ and S^2−^ 2*p*_3/2_, respectively (Fig. 2h). However, the binding energies of S^2−^ 2*p*_1/2_ and S^2−^ 2*p*_3/2_ in 1T_0.72_-MoS_2_@NiS_2_, or 1T_0.81_-MoS_2_@Ni_2_P heterostructures catalyst shift to 163.28 and 162.10 eV, respectively (Supplementary Fig. 11b). This negative-shift (0.13 eV) suggests little electron transfer between NiS_2_ (or Ni_2_P) and MoS_2_, also suggesting the reconfiguration of the electronic structure during the transferring of electron from Mo^4+^ to the surrounding Ni sites^48^. Interestingly, the 1T-phase contents in the 1T_0.81_-MoS_2_@Ni_2_P and 1T_0.72_-MoS_2_@NiS_2_ samples (81% and 72%, respectively) were higher than the 41% value observed for the 1T_0.41_-MoS_2_. Thus, phosphorus or sulfur implantation further facilitates the phase transformation of 1T_phase_-MoS_2_^24,32^. The reason may be that phosphorus can be simultaneously inserted into S–Mo–S atomic planes, inducing the glide of S atomic planes, affording in-plane heterostructures between 1T and 2H MoS_2_ domains (Supplementary Fig. 14), which is consistent with previous reports^31,32^. As shown in Fig. 2i, the Ni 2*p* spectrum of 1T_0.81_-MoS_2_@Ni_2_P shows two spin–orbit doublets at 856.6 and 874.9 eV corresponding to Ni^2+^ 2*p*_3/2_ and Ni^2+^ 2*p*_1/2_ oxidation states in Ni_2_P, respectively, and two satellite peaks (identified as “Satellite.”)^49^. Notably, compared with the binding energies of Ni 2*p*_3/2_ (857.4 eV) and Ni 2*p*_1/2_ (875.4 eV) of pure Ni_2_P, the two binding energies of Ni 2*p*_3/2_ and Ni 2p_1/2_ have a significant negative-shift of approximately 0.8 and 0.6 eV in 1T_0.81_-MoS_2_@Ni_2_P (Fig. 2i), respectively. This result implies the transfer of electrons from Mo^4+^ to Ni^2+^ sites in the 1T_0.81_-MoS_2_@Ni_2_P sample, resulting in a low-valence state and electron-rich structure of Ni^2+^ sites^50^. For pure NiS_2_ sample, the peaks of Ni 2*p*_3/2_ and Ni 2*p*_1/2_ located at 854.7 and 872.2 eV, and the corresponding satellites appear at 858.7 and 878.6 eV, respectively. However, the binding energies of Ni 2*p*_3/2_, Ni 2*p*_1/2_ and satellite in 1T_0.72_-MoS_2_@NiS_2_ sample (Fig. 2i) are positive-shifted to 856.6 (by 1.9 eV), 875.3 (by 3.1 eV), 861.8 (by 3.1 eV) and 862.2 eV (by 1.9 eV), respectively. The positive-shift of the Ni 2*p* binding energies peaks manifest a higher valence state, which are ascribed to Ni bonded to S and O atoms, such as sulfides or surface oxides/hydroxides^51^. For the 1T_0.81_-MoS_2_@Ni_2_P, the P 2*p* spectrum shows two peaks at 130.4 and 129.5 eV corresponding to P 2*p*_1/2_ and P 2*p*_3/2_, respectively, suggesting the existence of Ni_2_P. In addition, it also can be observed another peak at 134.7 eV of oxidized phosphate (P–O) species (Supplementary Fig. 13b), which is due to the partial oxidation of Ni_2_P in air. Notably, the binding energies of Ni 2*p*_3/2_ (852.7 eV) and P 2*p*_3/2_ (129.5 eV) are both shifted, indicating that charge transfer occurs from Ni to P, which can greatly promote the catalytic activity of 1T_0.81_-MoS_2_@Ni_2_P.

### Electrocatalytic HER performances in alkaline and acidic media

**Fig. 3:**
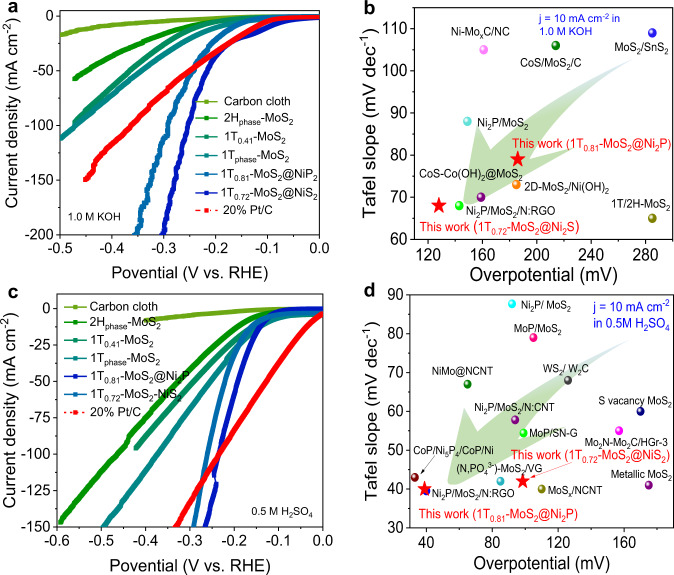
HER was performed in alkaline and acidic electrolytes. **a** LSV curves in 1 M KOH. **b** η_10_ and Tafel slopes for various Mo-based HER electrocatalysts in 1.0 M KOH. **c** LSV curves in 0.5 M H_2_SO_4_. **d** η_10_ and Tafel slopes for various Mo-based HER catalysts in 0.5 M H_2_SO_4_. (All LSV curves were corrected without iR-compensation).

1T_0.72_-MoS_2_@NiS_2_ and 1T_0.81_-MoS_2_@Ni_2_P electrodes exhibited attractive multi-heterogeneous interface edges, plentiful active sites, and abundant mass transfer and gas release channels and are expected to be used as very effective and stable catalysts for H_2_ production. First, we analyzed HER activities (in 1.0 M KOH) of the electrodes containing these electrodes. The 1T_0.72_-MoS_2_@NiS_2_ and 1T_0.81_-MoS_2_@Ni_2_P electrodes exhibit small overpotentials of 95 and 170 mV at 10 mA/cm^2^, respectively (see linear sweep voltammetry (LSV) results in Fig. 3a), which are better than the commercial Pt/C electrode (127 mV). To in-depth understand the HER kinetic mechanism, we calculated the Tafel slopes of these electrodes using the Tafel equation^52^ and obtained the smallest slopes equal to 68 and 79 mV/dec for the 1T_0.72_-MoS_2_@NiS_2_ and 1T_0.81_-MoS_2_@Ni_2_P electrodes, respectively (Supplementary Fig. 15a). These values are even closer to the Tafel slope of the Pt/C electrode (56 mV/dec). Thus, the 1T_0.72_-MoS_2_@NiS_2_ and 1T_0.81_-MoS_2_@Ni_2_P electrodes as active electrocatalysts exhibit the fastest HER processes and better reactivity, which is attributed to the multi-heterogeneous interface effect, a large number of defects, and a higher proportion of 1T_phase_-MoS_2_. Next, we evaluated the long-term cycling stability of the as-prepared electrodes using the chronopotentiometry technique at 10 and 30 mA/cm^2^, respectively. The 1T_0.72_-MoS_2_@NiS_2_ and 1T_0.81_-MoS_2_@Ni_2_P electrodes were very robust and exhibited negligible damping after 16 h measurement (Supplementary Fig. 15b), and the LSV curves measured before and after the long-term tests are almost the same (Supplementary Fig. 15c), demonstrating excellent long-term stability. Supplementary Fig. 15d lists the overpotential values for the 20.0 wt % Pt/C, 1T_0.72_-MoS_2_@NiS_2_, and 1T_0.81_-MoS_2_@Ni_2_P electrodes in 1.0 M KOH at various current densities. 1T_0.72_-MoS_2_@NiS_2_ electrodes exhibited lower overpotential. Generally, low overpotential and Tafel slope values demonstrated the superior HER catalytic activities, which was the case for our 1T_0.72_-MoS_2_@NiS_2_ and 1T_0.81_-MoS_2_@Ni_2_P electrodes. Moreover, 1T_0.72_-MoS_2_@NiS_2_ electrode has such excellent HER activity comparable to those of as-reported Mo-based materials (Fig. 3b) and composites and various representative catalysts^30,31,33,37–40^ (Supplementary Table 3). Thus, 1T_0.72_-MoS_2_@NiS_2_ electrode is a catalyst with the best HER activity in alkaline solutions.

To obtain the electrochemically active area (ECSA) of the 1T_0.72_-MoS_2_@NiS_2_ and 1T_0.81_-MoS_2_@Ni_2_P electrodes, the double-layer capacitance (*C*_dl_) was calculated because the two values are proportional to each other. Therefore, we tested their cyclic voltammetry (CV) by continuously increasing scanning speed (Supplementary Fig. 16a-c) in order to obtain the CV curve of the electrode materials in the non-Faraday region (−0.2 to 0.4 V). Then, as shown in Supplementary Fig. 16d, the *C*_dl_ was calculated from the plot slope (slope = 2*C*_dl_) between current-density difference (∆*j*) (0.15 V vs. RHE) and scan rate. The 1T_0.72_-MoS_2_@NiS_2_ electrodes possessed the highest *C*_dl_ value (*C*_dl_ = 359.7 mF/cm^2^), suggesting a multi-heterogeneous interface could be effectively enhanced conductivity and exposed more active sites of as-prepared electrodes. We recorded the electrochemical impedance spectra (EIS). The corresponding Nyquist (Supplementary Fig. 17) of the 1T_0.72_-MoS_2_@NiS_2_ electrode showed the lowest value for the charge transfer resistance (*R*_ct_). Thus, it possessed very favorable charge transfer kinetics. To further reveal the intrinsic catalytic activity of each active sites, the turnover frequency (TOF) is also calculated^53^. Based on the above-mentioned analysis, CV approach is regarded as the promising way to determine reasonable results (Supplementary Fig. 18). The TOF value of 1T_0.81_-MoS_2_@Ni_2_P (3.56 S^−1^) and 1T_0.72_-MoS_2_@NiS_2_ (2.26 S^−1^) heterojunction catalyst at the overpotential of 200 mV is 18.7 and 11.9 times higher than of 2H_phase_-MoS_2_ catalyst (0.19 S^−1^) for HER, respectively (Supplementary Table 1). Typically, the amount of hydrogen evolution was measured of 1T_0.72_-MoS_2_@NiS_2_ catalyst in 1.0 M KOH solution (Supplementary Fig. 19), presenting HER Faraday efficiency of 97.6 ± 0.6%, owing to the synergistic effect of the phase, defect and interface engineering of electrocatalyst.

Next, we also studied the HER performance of all the as-prepared electrodes in 0.5 M H_2_SO_4_ (Fig. 3c). The HER catalytic performance of the 1T_0.81_-MoS_2_@Ni_2_P and 1T_0.72_-MoS_2_@NiS_2_ electrodes was significantly improved their HER activities according to the LSV data: their overpotential values at 10 mA/cm^2^ were as low as 38.5 and 152 mV, respectively, which is lower than the values for the electrodes containing 1T_0.41_-MoS_2_@Ni(OH)_2_ (236 mV), 1T_0.41_-MoS_2_ (389 mV), 1T_phase_-MoS_2_ (392 mV), and 2H_phase_-MoS_2_ (354 mV). The Tafel slopes for the 1T_0.81_-MoS_2_@Ni_2_P and 1T_0.72_-MoS_2_@NiS_2_ electrodes were 41 and 42 mV/dec (Supplementary Fig. 20a). These values were lower than the values obtained for 1T_0.41_-MoS_2_ (169 mV/dec), 1T_phase_-MoS_2_ (163 mV/dec), and 2H_phase_-MoS_2_ (189 mV/dec) electrodes and were better than the electrode based on 20 wt% Pt/C (86 mV/dec). It is probably because, in the acidic environment, the H_2_ desorption is the limiting step because H^+^ are abundant. The 1T_0.81_-MoS_2_@Ni_2_P electrode had a weaker adsorption capacity toward H_ads_ so it exhibits a better catalytic effect than 2H_phase_-MoS_2_^54^. Meanwhile, compared to the other electrodes, 1T_0.81_-MoS_2_@Ni_2_P also has a higher ECSA because it has a larger *C*_dl_ (*C*_dl_ = 106.15 mF/cm^2^, Supplementary Fig. 21) and, as a result, more catalytical sites, which significantly contributed to the overall activity. Furthermore, 1T_0.81_-MoS_2_@Ni_2_P also possesses a much smaller *R*_ct_, in contrast to other electrodes at 300 mV overpotential vs. RHE (Supplementary Fig. 22), revealing satisfied electron transport and good catalytic kinetics, which leads to high activity and low Tafel slope. Supplementary Fig. 20b shows that at 10 and 45 mA/cm^2^, 1T_0.81_-MoS_2_@Ni_2_P and 1T_0.72_-MoS_2_@NiS_2_ electrodes were very durable and possesses negligible damping after 16 h measurement, which displays excellent long-term stability. In addition, even after 16 h of a chronoamperometric stability test of the electrodes, the current density remains above 95% (Supplementary Fig. 20c), and there is only a slight deviation for the LSV recorded after the stability test, indicating that as-prepared electrodes have very good stability in an acidic environment. As to 20.0 wt% Pt/C, 1T_0.72_-MoS_2_@NiS_2_, and 1T_0.81_-MoS_2_@Ni_2_P electrodes in 0.5 M H_2_SO_4_, Supplementary Fig. 20d shows overpotentials vs. various current densities. 1T_0.81_-MoS_2_@Ni_2_P exhibits lower overpotential. We also compared the overpotentials (at 10 mA/cm^2^ in acidic medium) and Tafel slopes with previously excellent Mo-based electrocatalysts^8,32,37,55–57^ (Fig. 3d and Supplementary Table 4). Catalytic HER performance of 1 T_0.81_-MoS_2_@Ni_2_P is also superior. Afterward, the amount of hydrogen evolution of 1T_0.81_-MoS_2_@Ni_2_P catalyst was given in Supplementary Fig. 23, demonstrating a promising Faraday efficiency of 98.7 ± 0.5% towards real water splitting into hydrogen. Based on the above-mentioned results, 1T_0.81_-MoS_2_@Ni_2_P multi-heterogeneous interface catalyst shows the remarkable intrinsic HER activities in acidic medium mainly attributed to multi-heterointerface interface edges active sites. In addition, as-synthesized 1T_0.72_-MoS_2_@NiS_2_ (or 1T_0.81_-MoS_2_@Ni_2_P) catalyst also exhibits excellent OER and overall-water splitting catalytic activity (Please see Supplementary Information for details, Supplementary Figs. 25–27).

### Theoretical calculation and mechanisms analysis of the surface electronic structure and HER activation energy for the as-prepared electrocatalysts

**Fig. 4:**
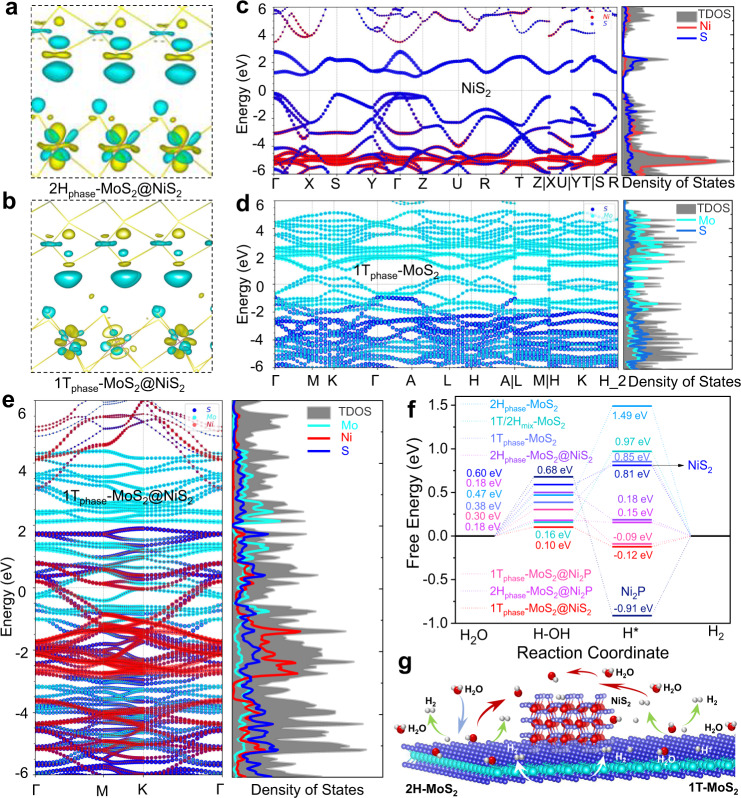
Theoretical calculation and mechanisms analysis of the surface structure and HER activation energy of the as-prepared electrocatalysts. the deformation of the electronic density of **a** 2H_phase_-MoS_2_@NiS_2_ and **b** 1T_phase_-MoS_2_@NiS_2_ interface, in which yellow/green isosurfaces correspond to positive/negative spin densities (0.00295308 e/Å^3^). Band structure and density of states (DOS) for **c** NiS_2_, **d** 1T_phase_-MoS_2_ and **e** 1T_phase_-MoS_2_@NiS_2_. **f** Free-energy diagrams for HER on the 2H_phase_-MoS_2_, 1T_phase_-MoS_2_, pure Ni_2_P, pure NiS_2_, 1T/2H_mix_-MoS_2_, 2H_phase_-MoS_2_@Ni_2_P, 2H_phase_-MoS_2_@NiS_2_, 1T_phase_-MoS_2_@NiS_2_ and 1T_phase_-MoS_2_@Ni_2_P interface edge. **g** Schematics showing water activation, *H intermediate formation and hydrogen generation on multi-heterojunction electrocatalysts.

To explain the distinguished synergistic effect of 1T_0.72_-MoS_2_@NiS_2_ (or 1T_0.81_-MoS_2_@Ni_2_P) multi-heterogeneous interface catalysts, Density functional theory (DFT) calculations were also performed. Model building and computational parameters can be seen in the “Methods” section. Firstly, the interfacial electron interaction was investigated. The charge difference images (Fig. 4a, b and Supplementary Fig. 37) reveal the charge transfer from 1T_0.41_-MoS_2_ to the Ni_2_S or/and Ni_2_P interface, and the introduction of 1T-phase is more conducive to charge transfer from MoS_2_ to NiS_2_ or Ni_2_P interface, which significantly increases the interface electron concentration and thus improves its activity. To better understand the surface electronic structure reconfiguration of MoS_2_ through a coordinated phase transition and interface regulation in theory, the band structure and density of states (DOS) of bare NiS_2_, Ni_2_P, 2H_phase_-MoS_2_, 1T_phase_-MoS_2_, 2H_phase_-MoS_2_@NiS_2_, 2H_phase_-MoS_2_@Ni_2_P, 1T_phase_-MoS_2_@NiS_2_, and 1T_phase_-MoS_2_@Ni_2_P (Fig. 4c–e and Supplementary Figs. 38–40) obtained using the hybrid DFT-HSE06 exchange–correlation functional, which is presented in the Supplementary Information. The calculation results show that the bare NiS_2_ exhibits typical semiconductor characteristics (Fig. 4c), with a narrow bandgap equal to 0.68 eV (Supplementary Figs. 38 and 39a). The band structure of 1T_phase_-MoS_2_ (Fig. 4d) and 1T_phase_-MoS_2_@NiS_2_ (Fig. 4e) exhibited a certain zero bandgap, indicating a complete transition from the semiconductor phase (0.91 eV) to the metallic phase (0 eV) with improved conductivities^27^. Notably, the intensity of PDOS of 1T_phase_-MoS_2_@NiS_2_ was higher than that of 1T_phase_-MoS_2_ and NiS_2_ at the Fermi level (Supplementary Figs. 38 and 39). Thus, the electron mobility of the 1T_phase_-MoS_2_@NiS_2_ catalysts was more favorable for the efficient charge transfer, which agrees consistent with the EIS test results^58^. Moreover, the PDOS results imply that the NiS_2_ interface hybrid generates some new interface electronic states in 1T_phase_-MoS_2_ (Supplementary Fig. 39c), which was very likely because of the hybridization of the *d*-orbital of Mo and an empty *d*-orbital of Ni. Thus, higher HER activity of 1T_phase_-MoS_2_@NiS_2_ in comparison to 1T_phase_-MoS_2_ agrees with the Fermi level DOS (Fig. 4d, e). Thus, the actual electrochemical performance would show even faster conductivity and charge transfer kinetics.

To reveal further the relationship of HER activity of catalysts with phase structure and heterojunction-interface, we used DFT to calculate the optimized structures and free-energy diagrams for HER on 2H_phase_-MoS_2_, 1T_phase_-MoS_2_, 1T/2H_mix_-MoS_2_, pure Ni_2_P, pure NiS_2_, 2H_phase_-MoS_2_@NiS_2_, 2H_phase_-MoS_2_@Ni_2_P, 1T_phase_-MoS_2_@NiS_2_, and 1T_phase_-MoS_2_@Ni_2_P catalysts with partially multi-heterojunction interface modification. As shown in Fig. 4f and Supplementary Fig. 42, the reaction pathway for alkaline HER is constructed^59,60^, including prior H_2_O dissociation to form H* intermediates (Volmer step) and hydrogen generation (Tafel step or Heyrovsky step). However, the energy of the intermediate state H*(ΔG_H*_) is a critical indicator of the ability of hydrogen evolution (Tafel step or Heyrovsky step)^35,59^. Figure 4f displays the calculated free-energy diagram on the most stable energy of the 2H_phase_-MoS_2_, 1T_phase_-MoS_2_, Ni_2_P, NiS_2_, 2H_phase_-MoS_2_@Ni_2_P, 2H_phase_-MoS_2_@NiS_2_, 1T_phase_-MoS_2_@NiS_2_, and 1T_phase_-MoS_2_@Ni_2_P catalysts (Supplementary Fig. 41). For 2H_phase_-MoS_2_, the ΔG_H*_ is very positive (1.49 eV), indicating that there is a strong interaction between H* and 2H_phase_-MoS_2_, showing poor HER reaction kinetics. More importantly, MoS_2_ shows unfavorable catalyst-OH_ad_ energetics (Δ*G*_H2O_ = 0.82 eV), suggesting that the relatively high activated H_2_O-adsorption energy will hinder the decomposition of H_2_O into H* intermediates and results in slow HER kinetics. The introduction of the 1T/2H_mix_-phase into MoS_2_ can obviously decrease the value of Δ*G*_H*_ to 0.97 eV and Δ*G*_H2O_ to 0.16 eV, implying promoted HER activity compared to 2H_phase_-MoS_2_. Notably, constructing multi-heterointerface interface edges active sites with NiS_2_ can provide the active sites for –OH adsorption, and the followed Δ*G*_H2O_ and Δ*G*_H*_ are decreased to 0.10 and −0.12 eV on the 1T_phase_-MoS_2_@NiS_2_ interface, indicating the 1T/2H_mix_-phase and NiS_2_ nanoparticles are effective for cleaving HO–H bonds and weaker interaction between H*. Also, the charge transfer from Ni_2_P to the MoS_2_ is verified by the DFT calculations, and hence there is a more optimal Δ*G*_H*_ value of about −0.09 eV. Hence, the NiS_2_ (or Ni_2_P) can act as a promoter of H_2_O dissociation and form hydrogen intermediates which then adsorb on nearby MoS_2_ catalyst sites. In this way, the multi-heterointerface can also accelerate the subsequent generation of H_2_. The reaction pathways on the single side (such as Ni_2_P, NiS_2_, and MoS_2_) of the interface have also been shown in Fig. 4f and Supplementary Fig. 42. These both show there is more unfavorable energetics than that of the synergetic pathway on MoS_2_@NiS_2_ or MoS_2_@Ni_2_P interface. The reason is that H* adsorbed on the surface of 2H_phase_-MoS_2_ bounds to Mo atoms, and strong Mo–H strength and poor conductivity. However, H* can be absorbed not only by the 1T_phase_-MoS_2_@NiS_2_ surface. Ni atoms possess empty *d* orbitals capable of binding H atoms, thereby weakening the Mo–H strength. More importantly, the introduction of the 1T-phase not only increases its electrical conductivity but also creates abundant active sites at the multi-heterojunction interface edges, which synergistically promote HER activity (Fig. 4g). Thus, our work demonstrates a novel and efficient design to create multi-heterogeneous interfacial electrocatalysts without noble metal materials and with excellent HER activity.

### In situ electrochemical-Raman spectroscopy

To better understand the active sites of the 1T_0.81_-MoS_2_@Ni_2_P and 1T_0.72_-MoS_2_@NiS_2_ electrodes during the HER process, we used in situ Raman spectroscopy to study it (Supplementary Fig. 46). The in situ Raman test system is shown in Supplementary Fig. 46a. The as-prepared sample, Ag/AgCl, and Pt wires were used as working electrode, reference electrode, and counter electrode, respectively. In addition, the electrolyte was 1.0 M KOH. As shown in Supplementary Fig. 46c, d, the Raman spectra of 1T_0.72_-MoS_2_@NiS_2_ electrode collected under potentiostatic conditions at stepped potential values from 0 V to −1.5 V. The results show that three characteristic peaks (147.3, 235.4, and 335.2 cm^−1^ are attributed to *J*_1_, *J*_2_, and *J*_3_ vibrations) of 1T-MoS_2_, two characteristic peaks (382 and 407 cm^–1^ are attributed to the *E*_2g_^1^ and *A*_1g_ vibrational bands) of 2H-MoS_2_, and a vibrational peak (437.3 cm^−1^) of Ni–S. However, when the 1T_0.72_-MoS_2_@NiS_2_ sample was put in the electrolyte solution (1.0 M KOH solution), at different applied voltages from the −0.4 to −1.5 V during electrocatalytic HER, these Raman peaks are significantly enhanced. In addition, many new peaks aroused at 152, 188, 222, 284, 322, 453, 500, 548 cm^–1^ of MoS_2_, respectively^61^. The changes of these Raman peaks indicate that new chemical bonds are formed between our samples and the functional groups of –OH, H^+^, and H_2_O molecules in the electrolyte, suggesting that it has a strong absorption capacity of ions and H_2_O molecules. In addition, it can be observed for two slight new peaks of 429 and 488 cm^−1^ under the bias potential of −0.4 V (Supplementary Fig. 46d), corresponding to the *v*_Ni-OH_ band of our samples. This result indicates the adsorbed H_2_O molecules during the cathodic polarization process are decomposed into H_ads_ species and OH^−^ ions^62^. More importantly, the intensity of these characteristic peaks of 1T_0.72_-MoS_2_@NiS_2_ are increased significantly as the potential varies from −0.4 to −1.5 V. It may be due to the OH^−^ being driven to adsorb on Mo, Ni, S atoms in the alkaline medium, and then OOH* intermediates are formed^63^. As to 1T_0.81_-MoS_2_@Ni_2_P sample, we also obtained similar results (Supplementary Fig. 46e, f). We used in situ growth of NiS_2_ (or Ni_2_P) nanoparticles on the entire surface of 1T-2H MoS_2_ microspheres to construct multi-heterojunction interface, which may generate Ni–Mo metal bonds, thereby increasing the number of effective active sites of the catalyst. Due to the introduction of Ni atoms, there is a strong interaction between Ni and Mo atoms on the surface of the catalyst, thereby increasing the local electronic state of Mo atoms, reducing the hydrogen-adsorption energy of the H^+^ on Mo atoms, and thus improving its intrinsic catalytic activity.

### X-ray absorption spectroscopy

**Fig. 5:**
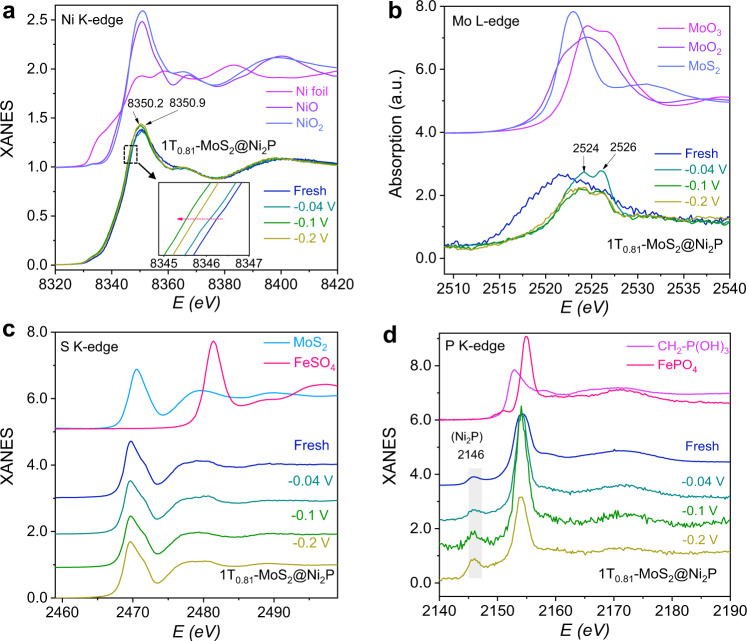
XANES spectra measurements of fresh 1T_0.81_-MoS_2_@Ni_2_P catalyst and after being used in the HER process at −0.04, −0.1, and −0.2 V vs RHE, respectively. **a** Ni *K*-edge XANES spectra of 1T_0.81_-MoS_2_@Ni_2_P catalyst and standards Ni foil, NiO, and NiO_2_. Inset, Magnified pre-edge XANES region. **b** Mo *L*-edge XANES spectra of 1T_0.81_-MoS_2_@Ni_2_P catalyst and standards MoS_2_, MoO_2_, and MoO_3_. **c** S *K*-edge XANES spectra of 1T_0.81_-MoS_2_@Ni_2_P catalyst and standards MoS_2_, and FeSO_4_. **d** P *K*-edge XANES spectra of 1T_0.81_-MoS_2_@Ni_2_P catalyst and standards CH_2_-P(OH)_3_, and FeSO_4_.

To investigate electronic states of catalysts, X-ray absorption near-edge structure (XANES) spectra were measured on the fresh catalysts and those after being used in the HER process at three representative potentials (−0.04, −0.1, and −0.2 V), near the onset potential and the overpotential at the current densities of 5 and 10 mA cm^–2^ (for 1T_0.81_-MoS_2_@Ni_2_P sample), respectively. Figure 5a presents Ni *K*-edge XANES spectra of 1T_0.81_-MoS_2_@Ni_2_P catalyst recorded at different applied potentials and reference spectra of Ni foil, NiO, and NiO_2_. From the fresh catalyst to that under the −0.04 V potential condition, the absorption edge is shifted to the lower energy side by ~0.5 eV, along with a broadening of the white-line peak, meaning a decrease of the Ni oxidation state. Moreover, when cathodic potentials of −0.04 and −0.1 V versus RHE were applied, a further shift of the absorption edge towards lower energy by ~0.2 eV occurs in relation to the case under the −0.04 V potential condition, implying a distinct decrease in the Ni valence state in 1T_0.81_-MoS_2_@Ni_2_P during the HER. Notably, all catalyst spectra exhibit white line at 8350.2-8350.9 eV (Supplementary Fig. 47), corresponding to the 1*s* to 4*p* electronic transition, indicating Ni–O local coordination similar to NiOOH and Ni oxides^64^. Using the edge positions of NiO and NiO_2_ as references, the Ni average valence state is determined as +3.3, +2.2, +1.8, and +2.0 for the fresh and those used at −0.04, −0.1, and −0.2 V, respectively (Supplementary Table 9). Therefore, Ni cations are reduced under working conditions, which is consistent with the Ni 2*p* XPS results (Supplementary Fig. 28d). In addition, 1T_0.72_-MoS_2_@NiS_2_ (Supplementary Fig. 48) and 1T_0.41_-MoS_2_@Ni(OH)_2_ (Supplementary Fig. 49) show similar behavior as Ni valency is decreased from +3.6 (fresh) to +2.4 (−0.1 V) and from +2.7 (fresh) to 1.8 (−0.2 V), respectively. In addition, oxidation of Mo from +4 to +6 after catalysis is observed as shown in Fig. 5b. The fresh 1T_0.81_-MoS_2_@Ni_2_P exhibit much broader Mo *L*_3_ XANES absorption than those of the Mo standards (MoS_2_, MoO_2_, and MoO_3_), and its lower edge position indicates reduced Mo admixture. In most cases, the broadening of XANES relates to a lack of crystallinity. Interestingly, after the HER reaction, the broad peak is shifted to higher energy and split, indicating *4dt*_2g_ and *4de*_g_ absorption bands of MoO_3_^65^ located at 2524 and 2526 eV, respectively. These results indicate the valence states of Mo cations are increased from approximately +4 to a higher oxidation state (+6) under working conditions. Moreover, sulfur does not take part in catalysis as shown in Fig. 5c. All S *K*-edge XANES spectra of catalysts before and after the reaction are similar and agree well with that of MoS_2_ standard. P *K*-edge XANES spectra of 1T_0.81_-MoS_2_@Ni_2_P and FePO_4_ are shown in Fig. 5d. The white line at 2154 eV belongs to PO_4_^2−^ associated with hybridized O 2*p*- P 3*p* absorption band^66^ whereas the original Ni_2_P species appear only as a minor peak at 2146 eV. The peak position and absorption line shape of Ni_2_P are close to that of Co_2_P indicates the valence state P^3-^^67^. The ratio of Ni_2_P to PO_4_^2−^ changes with the cathodic potential, and the highest ratio is 1:3 at −0.2 V. As to the fresh catalyst, the front peak is much weaker, which indicates that although there is a Ni–P bond, its signal is changed by the presence of some other elements. When cathodic potentials of −0.1 and −0.2 V versus RHE were applied, showing the highest intensity of the front peak, which indicates that both P and Ni are involved in the reaction during the HER process.

Overall, the XANES spectra studies provide clear evidence that the structures of as-prepared catalysts can drastically change under realistic catalytic conditions. The Ni site at the interface of heterojunction is most susceptible to low-valence induced by chemisorbed OH^−^ under electrochemical conditions. While the valence state of the Mo site at the interface increases, suggesting that the charge transfer (electron transfer from the Mo site to the Ni site) on the surface of the heterojunction catalyst is accelerated during the HER reaction. Therefore, reduced nickel possesses empty *d*-orbitals, which is beneficial to additional H binding ability. Moreover, it can decrease Mo–H bond strength, and so greatly enhance the HER catalytic activity of as-prepared catalysts.

## Discussion

In summary, we have constructed multi-heterogeneous-interface catalysts (1T_0.81_-MoS_2_@Ni_2_P and 1T_0.72_-MoS_2_@NiS_2_) by tuning its electronic structure of phase modulation synergistic with interfacial chemistry and defects to phosphorus or sulfur implantation strategies, which is an efficient approach to obtain abundant reactive sites of long-cycling and stable electrocatalysts for HER in alkaline and acidic surroundings. The as-achieved 1T_0.81_-MoS_2_@Ni_2_P and 1T_0.72_-MoS_2_@NiS_2_ electrodes only require small overpotentials of 38.9 (or 186) and 98.5 (or 128) mV to drive HER at 10 mA/cm^2^ and have low Tafel slopes: 41 (or 79) and 42 (or 68) mV/dec in 0.5 M H_2_SO_4_ (or 1.0 M KOH). Accordingly, these results show varieties of multi-heterogeneous interfaces in 1T_0.81_-MoS_2_@Ni_2_P and 1T_0.72_-MoS_2_@NiS_2_ electrodes, which can be considered versatile electroactive sites and facilitate electron transfer because of their unique heterogeneous effects. DFT calculation results also display that the introduction of metallic-phase MoS_2_ and intrinsic HER-active Ni-based materials can regulate MoS_2_ electronic structure effectively for making the bandgap narrower. In situ electrochemical-Raman spectroscopy indicates that the OH^−^ ions are driven to be adsorbed on Mo, Ni atoms in the alkaline medium, and then there form the OOH* intermediates. There is a strong interaction between Ni and Mo on the surface of the catalyst, thereby increasing the local electronic state of Mo atoms, reducing the hydrogen-adsorption energy for protons on Mo atoms, and thus improving its intrinsic catalytic. Additionally, XANES spectroscopy results imply that reduced Ni supply empty *d*-orbitals to facilitate H atom capture, and decrease Mo–H strength of 1T_0.81_-MoS_2_@Ni_2_P (or 1T_0.72_-MoS_2_@NiS_2_) catalysts that account for the outstanding HER properties with lower Tafel slopes and overpotentials compared with 2H_phase_-MoS_2_, 1T_phase_-MoS_2_ counterparts and other Mo-based catalysts. Thus, our work provides a new horizon for rationally designing multi-heterogeneous interfaces of non-precious electrocatalysts to realize excellent HER activities.

## Methods

### Synthesis of 1T_0.41_-MoS_2_

MoS_2_ microspheres were grown on carbon cloth (CC) hydrothermally. First, a CC (2 × 4 cm) was cleaned (for 15 min) using acetone and then sonicated in deionized water and ethanol for 10 min. Then, sodium molybdate (Na_2_MoO_4_·2H_2_O, 411.9 mg) and thiourea (CS(NH_2_)_2_, 608.96 mg) were added to deionized water (40 mL) and citric acid (20 mL). The mixture was magnetically stirred to form a cleaning solution, then placed into a 100 mL Teflon-lined autoclave and held in it at 180 °C for 12 h. Finally, the CC substrates with 1T_0.41_-MoS_2_ microspheres (denoted through the paper as 1T_0.41_-MoS_2_) were rinsed using deionized water and ethanol and vacuum-dried for 6 h at 60 °C. For comparison, deionized water was used as the solvent, and 2H_phase_-MoS_2_ microspheres were synthesized hydrothermally at 220 °C for 24 h from the same precursors.

### Synthesis of 1T_phase_-MoS_2_

We used Li-intercalated bulk MoS_2_ to prepare 1T_phase_-MoS_2_^68^. In an Ar-filled glove box, bulk MoS_2_ (1.0 g) prepared by stripping were dispersed in 15 mL of 2 M *n*-BuLi/hexane solution and stirred at ambient conditions for 48 h. The resulting black materials were repeatedly rinsed with anhydrous *n*-hexane and then centrifuged to eliminate *n*-butyl lithium excess and other solution impurities. The 1T_phase_-MoS_2_ powder was prepared and was then coated on the CC substrate. In order to promote better contact between 1T_phase_-MoS_2_ and CC substrate, we annealed (500 °C) the CC loaded with 1T_phase_-MoS_2_ sample under the protection of Ar gas.

### Synthesis of 1T_0.41_-MoS2@Ni(OH)_2_

We use a standard three-electrode system to prepare 1T_0.41_-MoS_2_@Ni(OH)_2_. 1T_0.41_-MoS_2_ acted as a working electrode, while Pt sheet and Ag/AgCl/3.5 M KCl acted as counter and reference electrodes. Ni(OH)_2_ was electrodeposited on the 1T_0.41_-MoS_2_ using 0.1 M Ni(NO_3_)_2_ at 5.0 mA/cm^2^ cathode current density applied for 300 s. 1T_0.41_-MoS_2_@Ni(OH)_2_ samples were rinsed with deionized water and ethanol several times and vacuum-dried at 60 °C.

### Synthesis of 1T_0.72_-MoS_2_@NiS_2_

The 1T_0.72_-MoS_2_@NiS_2_ multi-heterogeneous interfaces were prepared by the solid-vapor reaction method. First, a piece of 1T_0.41_-MoS_2_@Ni(OH)_2_ grew on CC was put into the quartz tube with 32.0 mg S powder and was then sealed. Secondly, the quartz tube was positioned inside a tube furnace and was calcinated at 500 °C for 60 min to obtain a 1T_0.72_-MoS_2_@NiS_2_ electrode.

### Synthesis of 1T_0.81_-MoS_2_@Ni_2_P

Similarly, the 1T_0.81_-MoS_2_@Ni_2_P multi-heterogeneous interfaces were also obtained by the solid-vapor reaction method. First, a piece of 1T_0.41_-MoS_2_@Ni(OH)_2_ grew on CC was put into the quartz tube with 31.0 mg red phosphorus and was then sealed. Secondly, the quartz tube was also calcinated at 580 °C for 1.0 h to prepare the 1T_0.81_-MoS_2_@Ni_2_P electrode. Additionally, 20 wt% Pt/C was also coated on CC substrate (2.0 mg/cm^2^) and was labeled as 20% Pt/C for comparison.

### Materials characterization

All as-synthetized electrodes were characterized by XRD (performed by Bruker D8 Advance instrument) and Raman spectroscopy (performed using Horiba LabRAB HR800 instrument). The sample morphologies were studied using SEM performed by Hitachi SU8010 instrument and TEM (performed by FEI Tecnai F30 instrument). XPS spectra were collected by the ESCALAB 250Xi instrument manufactured by ThermoFisher using Al Kα radiation.

### Electrochemical measurements

All electrochemical measurements were performed with a CHI 660E Electrochemical Workstation (CHI Instruments, Shanghai Chenhua Instrument Corp., China). The HER performance of different catalysts (1.0 cm^2^) was characterized using a three-electrode electrochemical cell in N_2_-saturated 1.0 M KOH and 0.5 M H_2_SO_4_ electrolyte, respectively. Before testing the polarization curve, we first perform cyclic voltammetry (CV) for more than 20 cycles to activate the as-prepared catalysts with a scan rate of 50 mV/s. The EIS tests were measured by AC impedance spectroscopy at the frequency ranges 10^6^ to 1.0 Hz at 300 mV. According to the Nernst equation (*E*_RHE_ = *E*_Hg/HgO_ + 0.059 pH + 0.098), where *E*_RHE_ was the potential vs. a reversible hydrogen potential, *E*_Hg/HgO_ was the potential vs. Hg/HgO electrode, and pH was the pH value of electrolyte. The electrochemical stability was evaluated by chronoamperometry measurements at a static overpotential, during which the current variation with time was recorded. The ECSA values were measured through CV in the selected non-faradaic range. The current densities have a linear relationship against different scan rates (10–60 mV/s) and the values of the slop were considered as twice of *C*_dl_. The Faraday efficiency of the as-fabricated electrodes was determined by the water drainage method, which can be found in Supplementary Note 2 in Supplementary Information for details. The OER tests were performed in O_2_-saturated 1.0 M KOH solution, and the others are the same as HER test conditions. The overall-water splitting performance was characterized in 1.0 M KOH using a two-electrode configuration, and the polarization curve was recorded at a scan rate of 5 mV/s. In order to better compare, Pt/C and IrO_2_ ink were also synthesized by placing 8 mg Pt/C and 8 mg IrO_2_ powder in the mixture of 700 µL ethanol, 300 µL deionized water, and 50 µL Nafion followed by ultrasonication for 30 min, respectively. Then the as-obtained ink was coated onto the carbon cloth (CC) with the loading mass density of about 3.0 mg/cm^2^ and was then dried at 60 °C. The long-term stability measurements were carried out using the chronoamperometry measurements. All polarization curves at 5 mV/s were corrected without iR-compensation.

### DFT theoretical calculation

#### Model building

According to the HRTEM micrographs, 1T_phase_-MoS_2_, 2H_phase_-MoS_2_, Ni_2_P, and NiS_2_ formed a multiphase heterojunction. 1T_phase_-MoS_2_@Ni_2_P interface, 1T_phase_-MoS_2_@NiS_2_ interface, 2H_phase_-MoS_2_@Ni_2_P interface, 2H_phase_-MoS_2_@NiS_2_ interface, bulk 1T_phase_-MoS_2_, 2H_phase_-MoS_2_ were also constructed as comparisons. Considering the Van der Waals forces between the two phases, the unrelaxed heterojunction interface distance was set to 3.0 Å. These original structures were obtained from Materials Project Database^69^.

#### Computational parameters

DFT calculation was applied to calculate electronic structures of two crystal structures by the partial augmented plane-wave method (PAW) implemented in the VASP^70^ using VASPKIT code for post-processing. Considering the heterojunction structure, the long-range force correction was considered by using the DFT-D3 correction method of Grimme^71^. The Perdew–Burke–Ernzerhof (PBE) generalized gradient approximation^72^ was implemented for exchange-correlation energy calculations using 550 eV kinetic energy cut off for the plane-wave basis. Then structural optimizations using a conjugate gradient (CG) method based on the pre-optimized structure were repeated until the maximum force component on each atom remained below 0.01 eV/Å. Monkhorst-Pack k-point meshes in the first Brillouin zone of the primitive cell were used the VASPKIT code recommended accuracy levels of 0.04 for the optimization calculation and 0.02 for the static calculation, respectively. After fully relaxing the structures, one final (electronic scf) step with the tetrahedron method using Blöchl corrections and denser k-meshes was employed for DOS calculation. In addition to the H adsorbed energy calculations, the frequency calculation of free H and free-energy correction at 298.15 K (including the entropy and zero-point energy contributions) were also calculated. To avoid abnormal entropy contribution, frequencies < 50 cm^−1^ are set to be 50 cm^−1^.

### XANES spectra measurements

The Mo *L*_3_-edge, P, S, and Ni *K*-edge XANES spectra were measured at the BL8 beamline of Synchrotron Light Research Institute (SLRI), Thailand^73^. The SLRI storage ring was operated at 1.2 GeV with an electron current of 80–150 mA. The incident X-ray beam was monochromatized with a double-crystal monochromator equipped with InSb (111) and Ge (220) crystals. XANES measurements were carried out in air at Ni K-edge and under He atmosphere at the lower edges on as-prepared catalysts embedded on carbon cloth and those used as the working electrode in electrochemical reaction with 1 M KOH solution. Cathode voltages from −0.04 V to −0.2 V vs. RHE (*E*_RHE_ = *E*_Ag/AgCl_ + 0.059 pH + 0.197) were applied for 160 s using an electrochemical workstation (Autolab PGSTAT204) before the XANES experiment. All XANES spectra were collected in fluorescence-yield mode using a 13-element Si drift detector. Ni and foils, and elemental S and P were used for photon energy calibration. The edge position was defined as the point corresponding to the maximum value in the derivative curves of the XANES spectra. Data normalization was carried out using the Athena software^74^.

## Supplementary information

Supplementary Information

Peer Review File

## Data Availability

The authors declare that the main data supporting the findings of this study are available within the article and its Supplementary Information. Extra data are available from the corresponding author upon request.
