# Supplementary material for: Interfacial electronic structure engineering on molybdenum sulfide for robust dual-pH hydrogen evolution
Source: Nat Commun. 2021 Sep 6;12:5260. doi: 10.1038/s41467-021-25647-8 (PMC8421379; doi:10.1038/s41467-021-25647-8)
Supplement: Supplementary file 1 — Supplementary Information [file 41467_2021_25647_MOESM1_ESM.pdf]

Supplementary Information for ***Nature Communications***

Interfacial Electronic Structure Engineering on Molybdenum Sulfide for Robust Dual-pH Hydrogen Evolution

Mingqiang Liu[1,2,†], Jia-Ao Wang[3,†], Wantana Klysubun[4], Gui-Gen Wang[1*], Suchinda Sattayaporn[4], Fei Li[1], Ya-Wei Cai[1], Fuchun Zhang[5], Jie Yu[1] & Ya Yang[2,6*]

[1] Shenzhen Key Laboratory for Advanced Materials, School of Materials Science and Engineering, Harbin Institute of Technology, Shenzhen, 518055, P. R. China.

[2] CAS Center for Excellence in Nanoscience, Beijing Key laboratory of Micro-nano Energy and Sensor, Beijing Institute of Nanoenergy and Nanosystems, Chinese Academy of Science, Beijing 101400, P.R. China.

[3] Department of Chemistry and the Oden Institute for Computational Engineering and Sciences, University of Texas at Austin, Austin, TX, 78712-0165, USA.

[4] Synchrotron Light Research Institute 111 Moo 6, University Ave., Muang Nakhon Ratchasima 30000, Thailand.

[5] School of Physics and Electronic Information, Yan'an University, Yan'an 716000, China.

[6] School of Nanoscience and Technology, University of Chinese Academy of Sciences, Beijing, 100049, P.R. China.

Address correspondence to G. G. Wang (email: wangguigen@hit.edu.cn) or Y. Yang (email: yayang@binn.cas.cn).

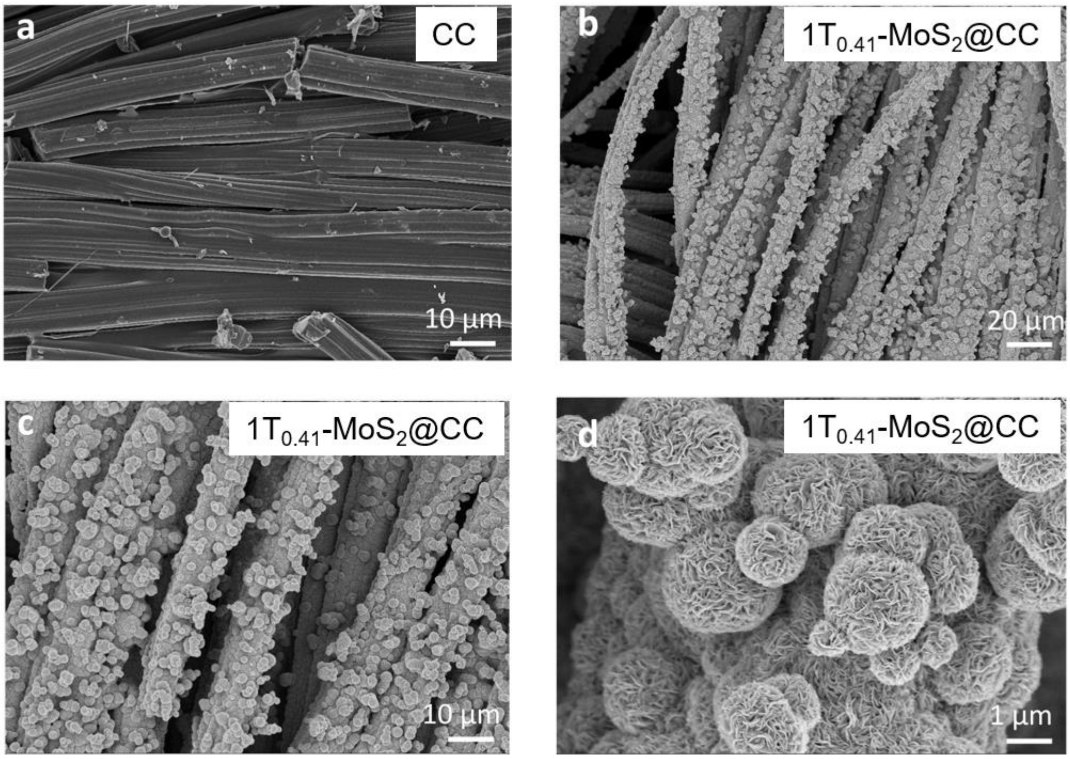

Supplementary Fig. 1. SEM images of **a** CC, **b-d** $1T_{0.41}$-MoS_2@CC.

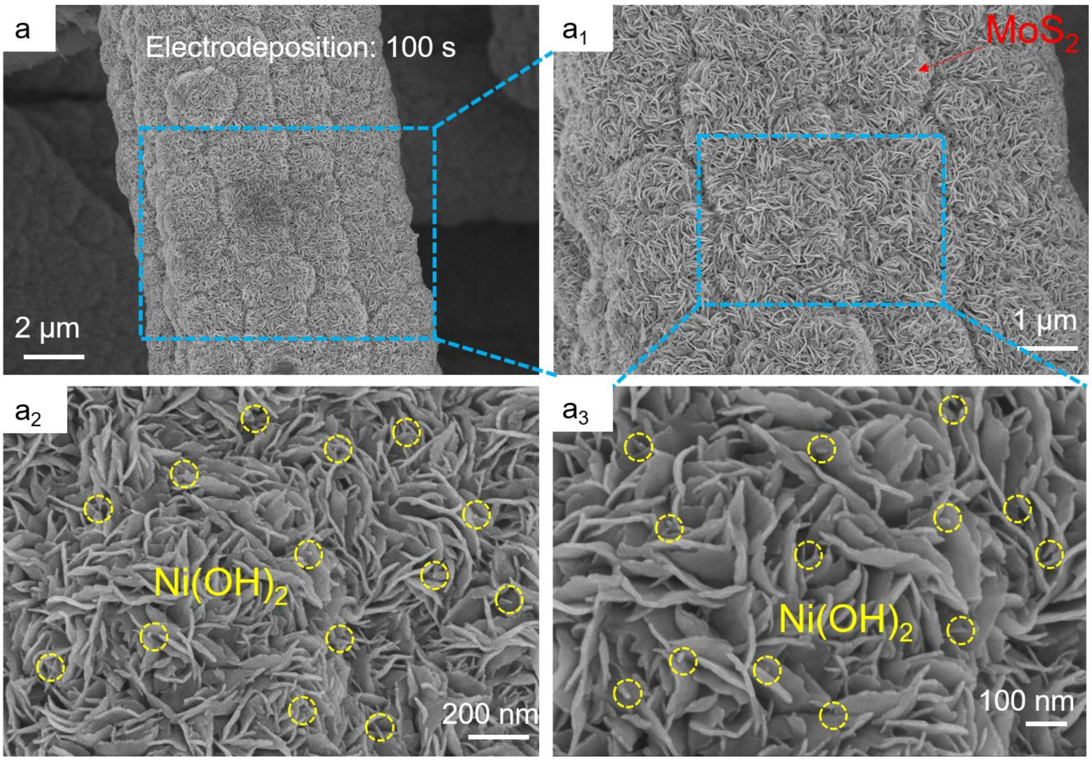

Supplementary Fig. 2. **a-a$_3$** SEM images of $1T_{0.41}$-MoS_2@$Ni(OH)_2$. $Ni(OH)_2$ nanoparticles were electrodeposited on the $1T_{0.41}$-MoS_2 using 0.1 M $Ni(NO_3)_2$ at 5.0 mA/cm^2 cathode current density applied for 100 s.

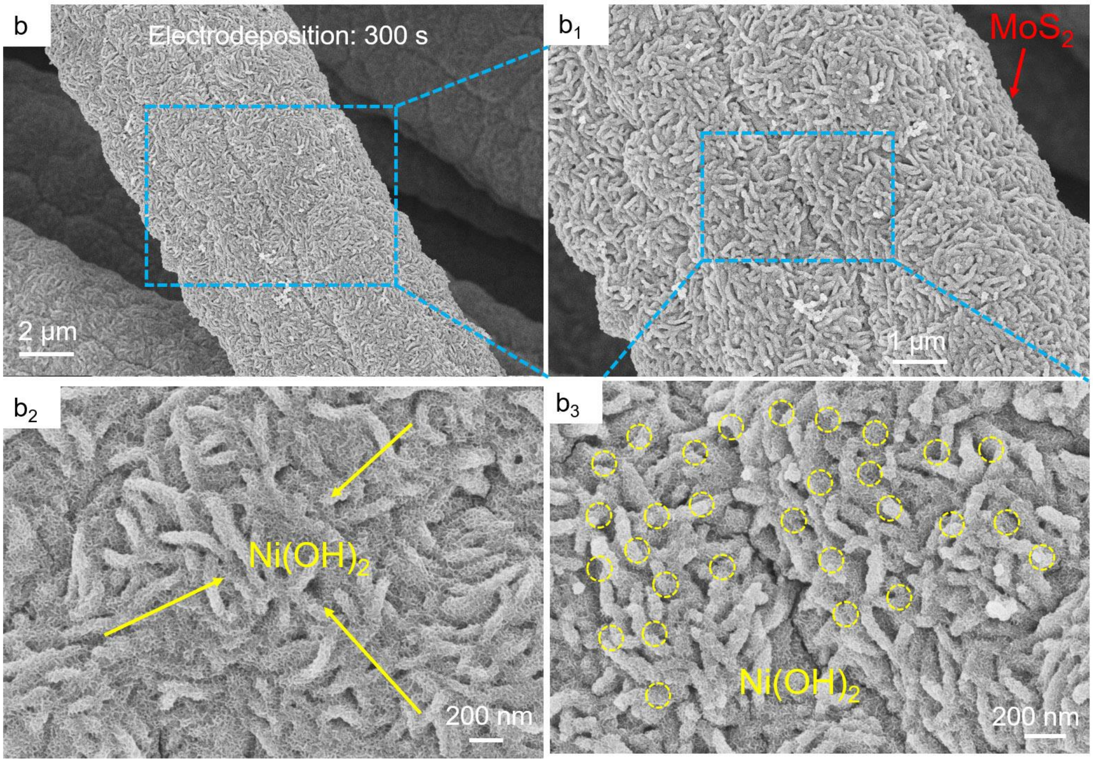

Supplementary Fig. 3. **b-b$_3$** SEM images of $1T_{0.41}$-MoS_2@$Ni(OH)_2$. $Ni(OH)_2$ nanoparticles were electrodeposited on the $1T_{0.41}$-MoS_2 using 0.1 M $Ni(NO_3)_2$ at 5.0 mA/cm^2 cathode current density applied for 300 s.

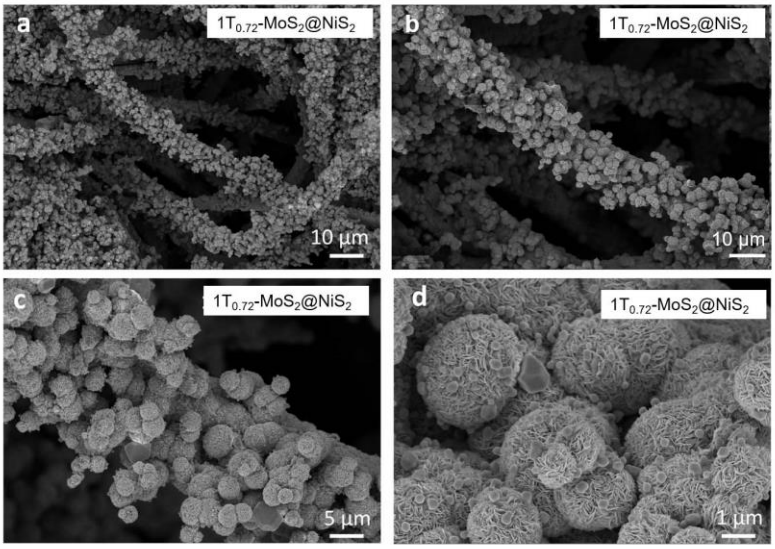

Supplementary Fig. 4. **a-d** SEM images of $1T_{0.72}$-MoS_2@NiS_2.

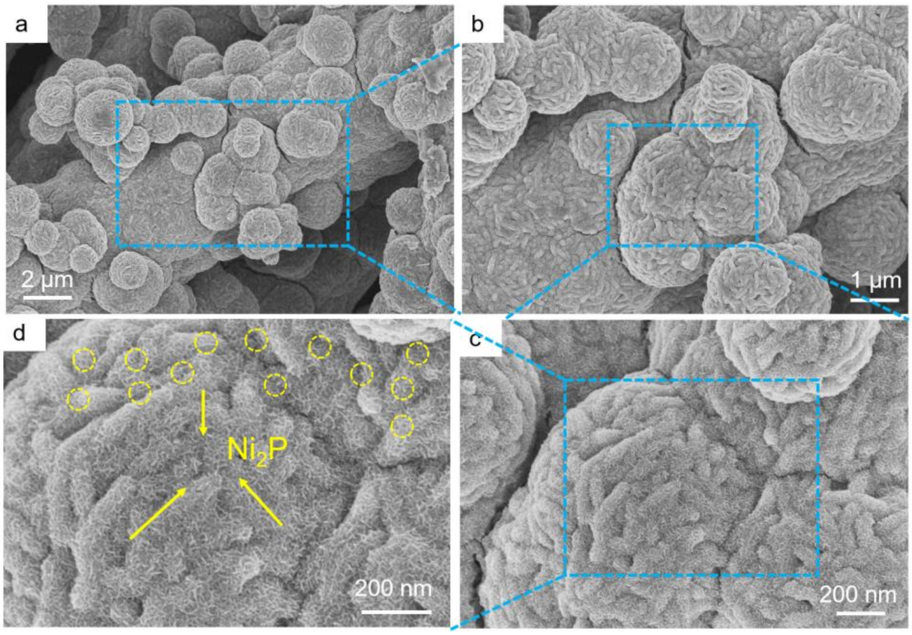

Supplementary Fig. 5. **a-d** SEM images of $1T_{0.81}$-MoS_2@Ni_2P.

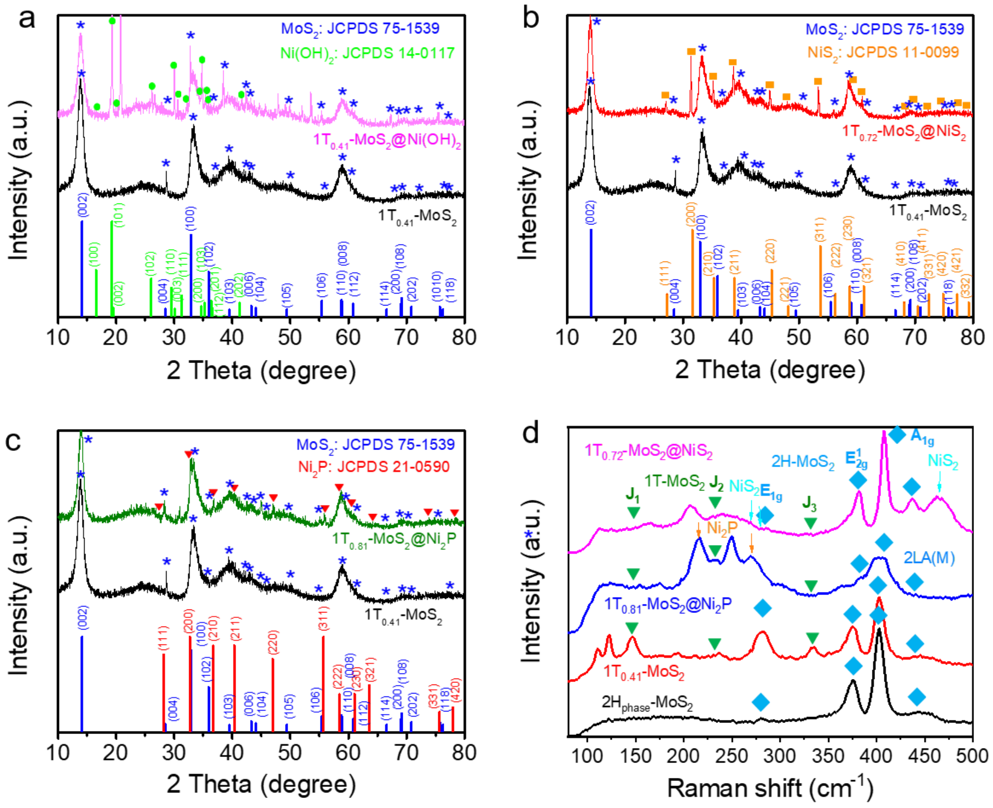

Supplementary Fig. 6. XRD patterns of as-synthesized materials on carbon cloth. **a** XRD patterns of $1T_{0.41}$-MoS_2 and $1T_{0.41}$-MoS_2@$Ni(OH)_2$. **b** XRD patterns of $1T_{0.41}$-MoS_2 and $1T_{0.72}$-MoS_2@NiS_2 catalysts. **c** XRD patterns of $1T_{0.41}$-MoS_2 and $1T_{0.81}$-MoS_2@Ni_2P catalysts. **d** Raman spectra of $2H_{phase}$-MoS_2, $1T_{0.41}$-MoS_2@$Ni(OH)_2$, $1T_{0.72}$-MoS_2@NiS_2 and $1T_{0.81}$-MoS_2@Ni_2P catalysts.

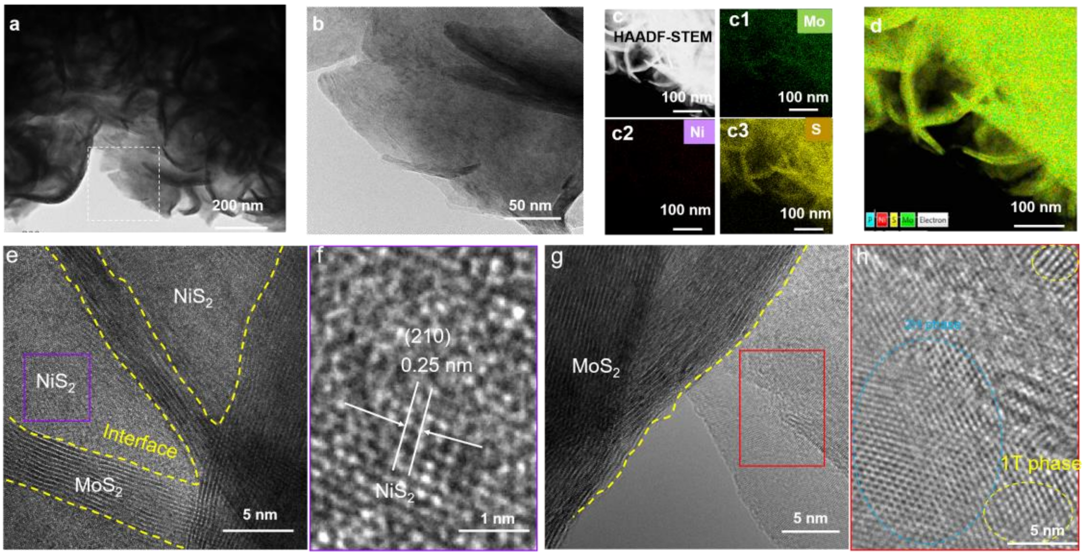

Supplementary Fig. 7. a, b Typical TEM images of $1T_{0.72}$-MoS_2@NiS_2. **c** EDS mapping of $\mathbf{c_1}$ Mo, $\mathbf{c_2}$ Ni, and $\mathbf{c_3}$ S elements of $1T_{0.72}$-MoS_2@NiS_2, and **d** overlap mapping of elements. **e-h** HRTEM images of $1T_{0.72}$-MoS_2@NiS_2.

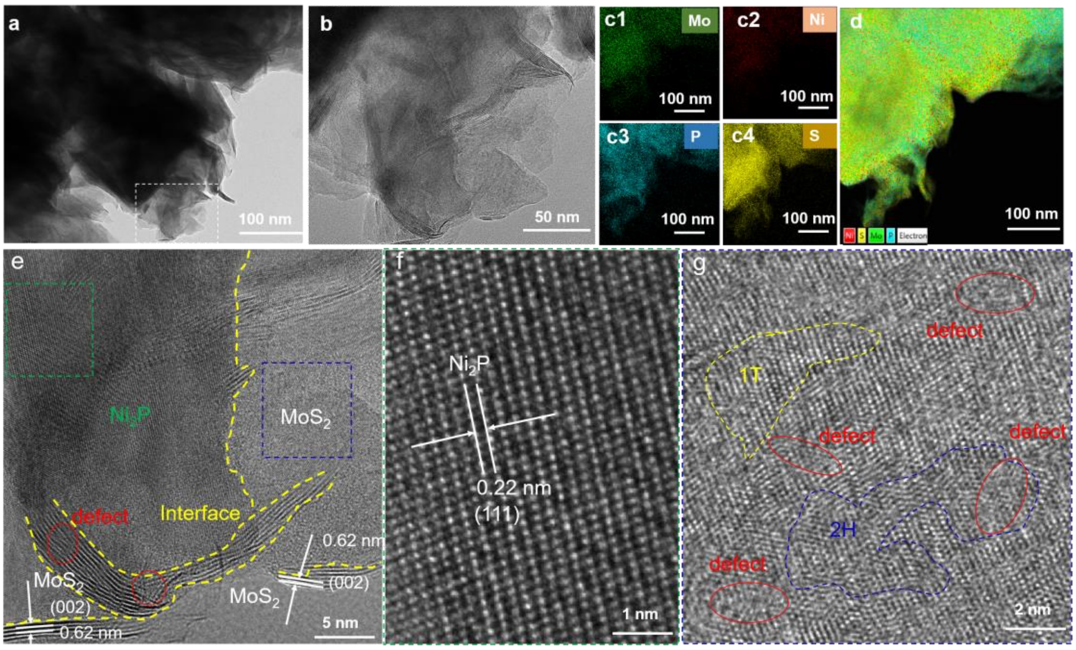

Supplementary Fig. 8. a, b Typical TEM images of $1T_{0.81}$-MoS_2@Ni_2P. **c** EDS mapping of **c_1** Mo, **c_2** Ni, **c_3** P and **c_4** S elements of $1T_{0.81}$-MoS_2@Ni_2P, and **d** overlap mapping of elements. **e-g** HRTEM images of $1T_{0.81}$-MoS_2@Ni_2P.

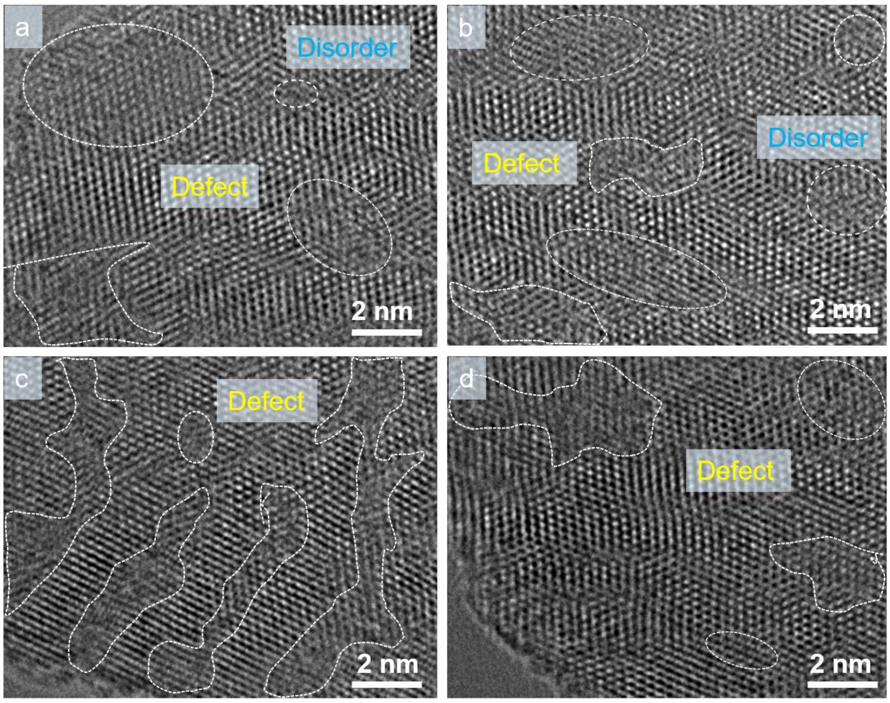

Supplementary Fig. 9. Typical HRTEM image of $1T_{0.81}$-MoS_2@Ni_2P.

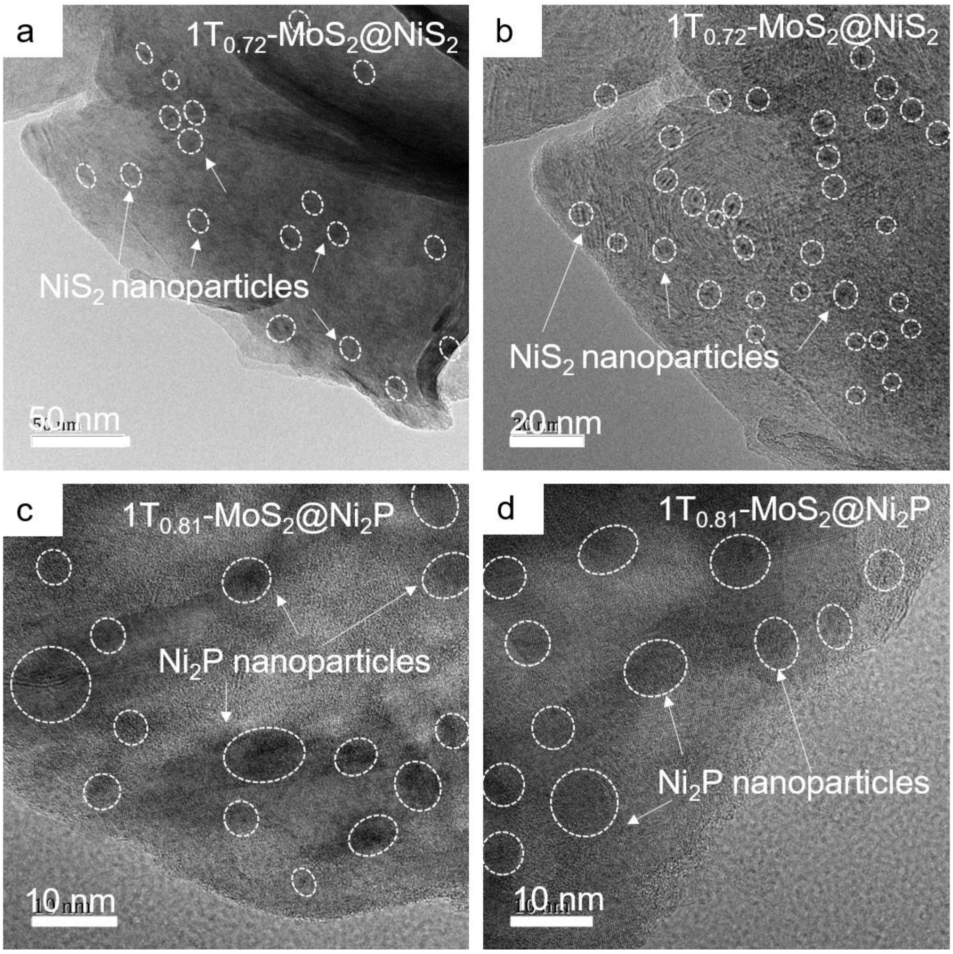

Supplementary Fig. 10. **a, b** Typical TEM images of $1T_{0.72}$-MoS_2@NiS_2 sample. **c-d** Typical TEM images of $1T_{0.81}$-MoS_2@Ni_2P sample.

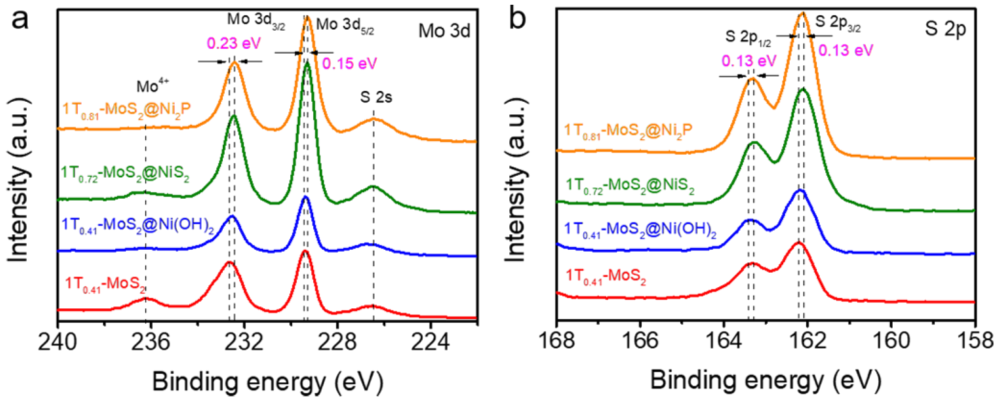

Supplementary Fig. 11. **a** The high-resolution Mo 3*d* core-level XPS spectra of $1T_{0.41}$-MoS_2 and $1T_{0.41}$-MoS_2@$Ni(OH)_2$, $1T_{0.72}$-MoS_2@NiS_2, and $1T_{0.81}$-MoS_2@Ni_2P. **b** The high-resolution S 2*p* core-level XPS spectra of $1T_{0.41}$-MoS_2 and $1T_{0.41}$-MoS_2@$Ni(OH)_2$, $1T_{0.72}$-MoS_2@NiS_2, and $1T_{0.81}$-MoS_2@Ni_2P.

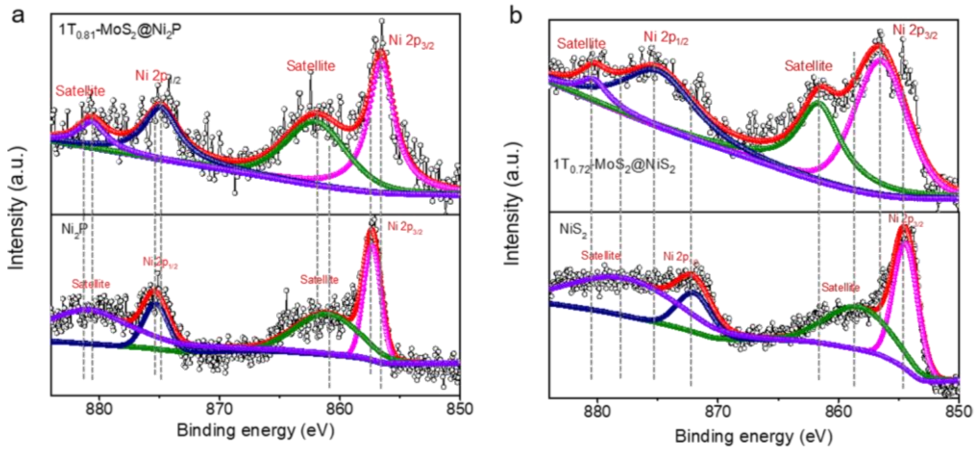

Supplementary Fig. 12. a XPS spectra of $1T_{0.81}$-MoS_2@Ni_2P catalyst and pure Ni_2P.

b XPS spectra of $1T_{0.72}$-MoS_2@NiS_2 catalyst and pure NiS_2.

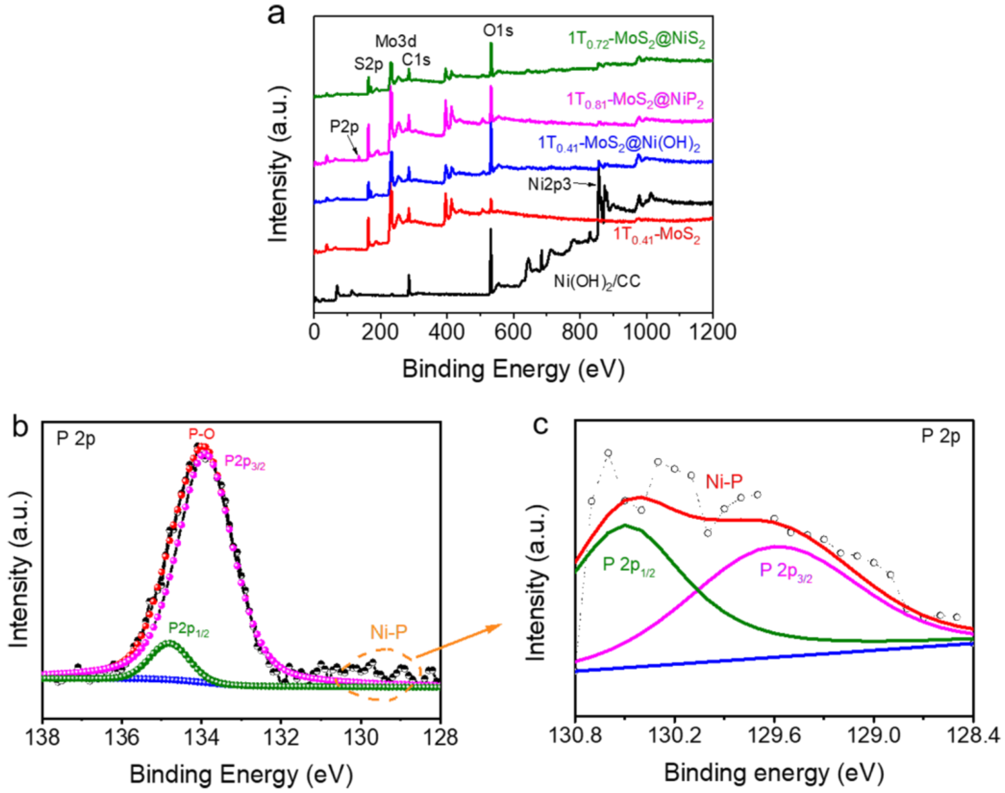

Supplementary Fig. 13. a Survey XPS spectra of $1T_{0.72}$-MoS_2@NiS_2, $1T_{0.81}$-MoS_2@Ni_2P, $1T_{0.41}$-MoS_2@$Ni(OH)_2$, $1T_{0.41}$-MoS_2, and $Ni(OH)_2$/CC, respectively. **b-c** High-resolution P 2*p* core-level XPS spectra of $1T_{0.81}$-MoS_2@Ni_2P.

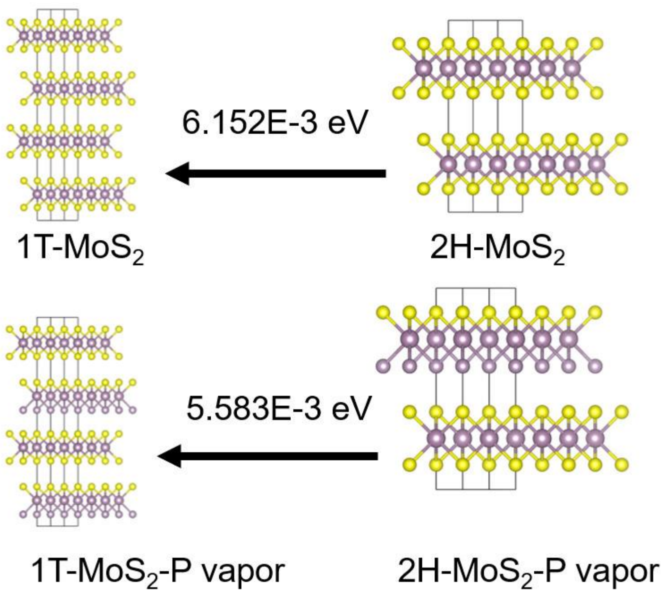

Supplementary Fig. 14. DFT calculation for comparing the phase-changing free energy of 2H→1T and 2H(P) →1T(P) to simulate the process.

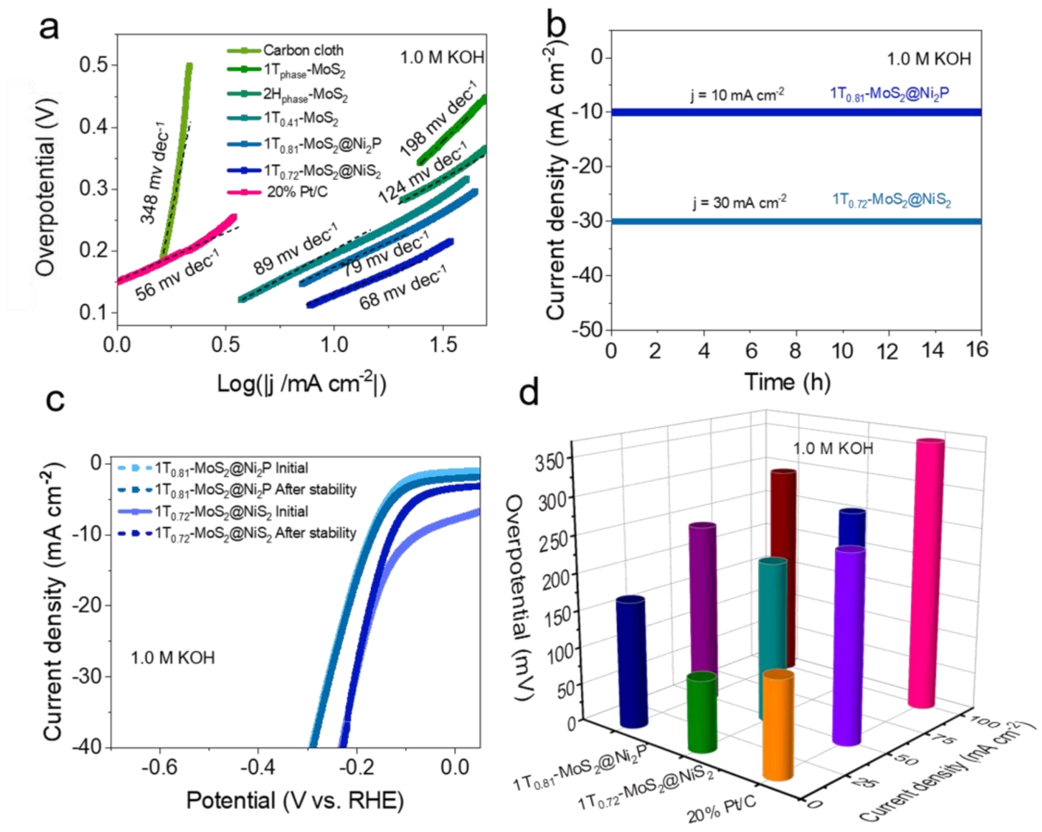

Supplementary Fig. 15. **a** Tafel plots in 1M KOH. **b** Plot of current density versus time at constant overpotential for $1T_{0.72}$-MoS_2@NiS_2 and $1T_{0.81}$-MoS_2@Ni_2P in 1.0 M KOH electrolyte for 16 h, showing its excellent stability performance during continuous tests. **c** LSV curves of durability of $1T_{0.72}$-MoS_2@NiS_2 and $1T_{0.81}$-MoS_2@Ni_2P. **d** Overpotentials at various current densities of 20.0 wt% Pt/C, $1T_{0.72}$-MoS_2@NiS_2, and $1T_{0.81}$-MoS_2@Ni_2P in 1.0 M KOH.

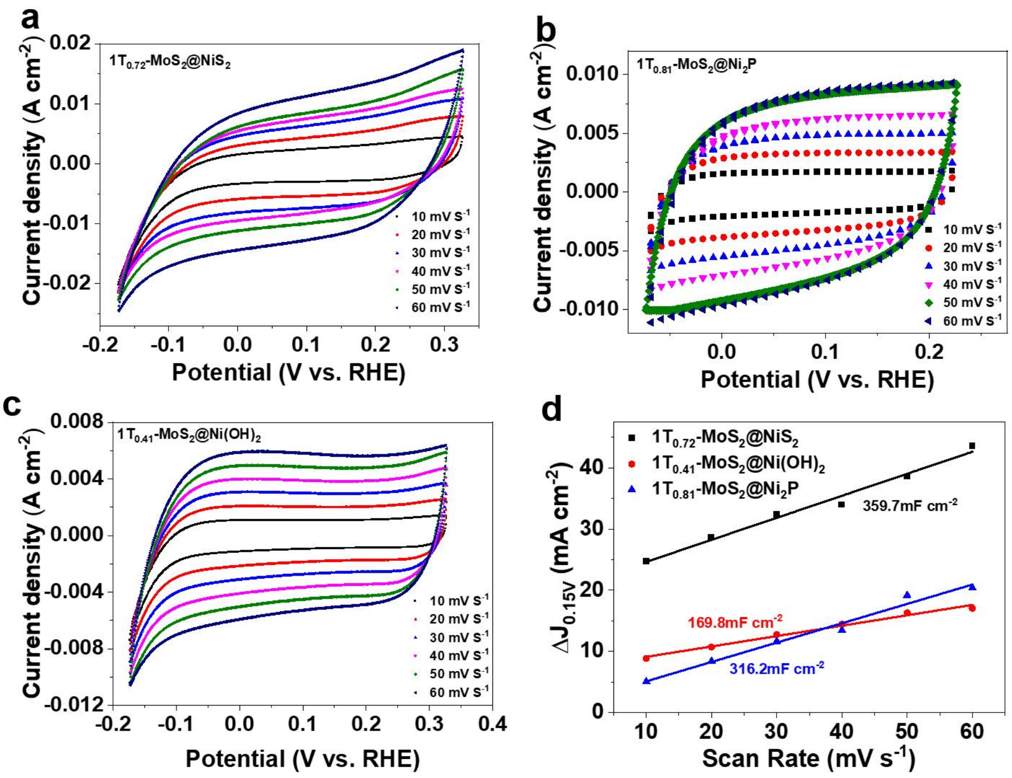

Supplementary Fig. 16. CV curves for **a** $1T_{0.72}$-MoS_2@NiS_2, **b** $1T_{0.81}$-MoS_2@Ni_2P, and **c** $1T_{0.41}$-MoS_2@$Ni(OH)_2$ with different rates from 10 to 60 mV S^{-1} in the region of -0.2-0.4 V in 1M KOH. **d** the capacitive current at 0.15 V as a function of the scan rate.

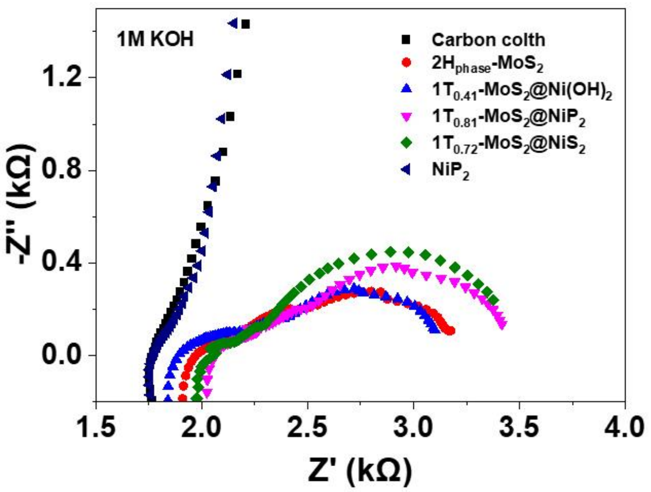

Supplementary Fig. 17. Nyquist plots of carbon cloth, $2H_{phase}$-MoS_2, $1T_{0.41}$-MoS_2@$Ni(OH)_2$, $1T_{0.81}$-MoS_2@Ni_2P, $1T_{0.72}$-MoS_2@NiS_2 and NiP_2 in 1.0 M KOH.

Supplementary Note 1

For rough estimation per-site turnover frequency (TOF), we previously carried out according to the previously approach adopted by Jaramillo et al.[1] In this way, the geometric areas of the assembled electrodes were hypothesized. Then TOF values were calculated. In order to ascertain the reasonable TOF values, cycle voltammetry (CV) method can be applied in this part. The TOF values (S^{-1}) were calculated with the following formula: TOF = I/2NF

I: current density extracted from the LSV curves;

F: Faraday constant;

N: the number of active sites.

Cycle voltammetry (CV) measurements were conducted between −0.2 V and 0.6 V vs. RHE in 1.0 M PBS at a scan rate of 50 mV S^{-1} (Supplementary Fig. 17). The absolute components of the voltammetric charges tested during one CV cycle were calculated. Assuming a one-electron process for both reduction and oxidation, the absolute charges were divided by two and the Faraday constant to obtain the number of active sites of the catalysts. The upper limit of active sites (N) for $1T_{0.72}$-MoS_2@NiS_2 and $1T_{0.81}$-MoS_2@Ni_2P catalysts could be calculated according to the equation: N = Q/2F. where F and Q are the Faraday constant and the whole charge of CV curve, respectively.

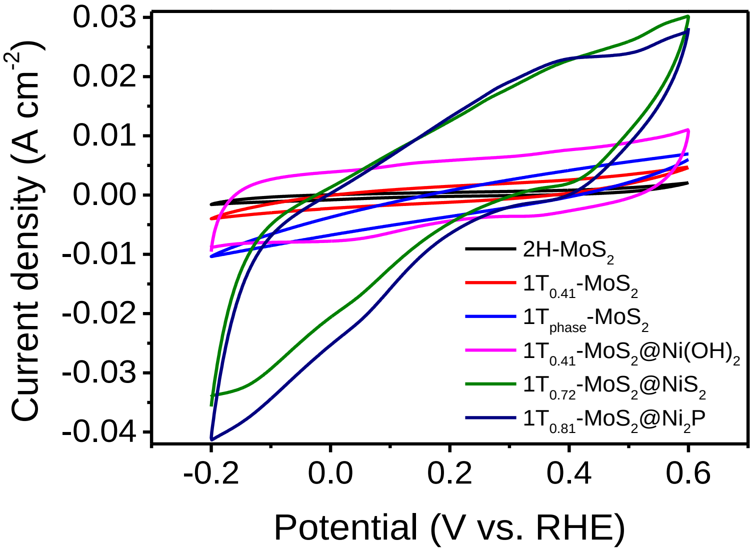

Supplementary Fig. 18. Cycle voltammetry curves of $2H_{phase}$-MoS_2, $1T_{0.41}$-MoS_2, $1T_{phase}$-MoS_2, $1T_{0.41}$-MoS_2@$Ni(OH)_2$, $1T_{0.72}$-MoS_2@NiS_2 and $1T_{0.81}$-MoS_2@Ni_2P in 1.0 M PBS at a scan rate of 50 $mV.S^{-1}$.

Supplementary Table 1. The turnover frequency (TOF) values of various HER electrocatalysts at different overpotentials in alkaline condition.

**Catalysts**	**TOF values in HER**		
	η = 100 mV	η = 200 mV	η = 300 mV
$1T_{0.81}$-MoS_2@Ni_2P	0.97 S^{-1}	3.56 S^{-1}	12.78 S^{-1}
$1T_{0.72}$-MoS_2@NiS_2	0.31 S^{-1}	2.26 S^{-1}	10.93 S^{-1}
$1T_{0.41}$-MoS_2@$Ni(OH)_2$	0.26 S^{-1}	0.89 S^{-1}	2.36 S^{-1}
$1T_{phase}$-MoS_2	0.12 S^{-1}	0.56 S^{-1}	1.17 S^{-1}
$1T_{0.41}$-MoS_2	0.09 S^{-1}	0.27 S^{-1}	0.88 S^{-1}
2H-MoS_2	0.06 S^{-1}	0.19 S^{-1}	0.56 S^{-1}

Supplementary Table 2. Comparison of TOF values of HER catalysts in alkaline condition.

Catalysts	TOF (H_2 S^{-1}@mV)	C_{dl} (mF cm^{-2})	Reference
$NiCo_2P_x$	0.056 S^{-1}@100 mV	24.2 mF cm^{-2}	Adv. Mater. **29**, 1605502 (2017)
$MoNi_4/MoO_{3-x}$	1.13 S^{-1}@100 mV	128 mF cm^{-2}	Adv. Mater. **29**, 1703311 (2017)
N-$NiCo_2S_4$	1.0 S^{-1}@125 mV	18 mF cm^{-2}	Nat. Commun. **9**, 1425 (2018).
NiO@1T-MoS_2	0.7 S^{-1}@130 mV	18.32 mF cm^{-2}	Nat. Commun. **10**, 982 (2019).
$Mo_1N_1C_2$	1.46 S^{-1}@150 mV	21.56 mF cm^{-2}	Angew. Chem. Int. Ed. **56**, 16086-16090 (2017).
Co-NiS_2	4.1 S^{-1}@200 mV	18.9 mF cm^{-2}	Angew. Chem. Int. Ed. **58**, 18676-18682 (2019).
MoO_3@MoS_2	1.93 S^{-1}@250 mV	337.3 mF cm^{-2}	Adv. Energy Mater. **8**, 1800734 (2018)
$NiMoO_x$@NiMoS	0.28 S^{-1}@50 mV	23.4 mF cm^{-2}	Nat. Commun. **11**, 5462 (2020)
Ni-Co@1T-MoS_2	0.98 S^{-1}@100 mV	108.1 mF cm^{-2}	Nat. Commun. **8**, 15377 (2017)
1T-2H MoS_2	13.14 S^{-1}@250 mV	2.41 mF cm^{-2}	Adv. Energy Mater. **8**, 1801345 (2018)
(N, PO_4^{3-})-MoS_2/VG	0.03 S^{-1}@100 mV	140 mF cm^{-2}	Angew. Chem. Int. Ed. **131**, 16435-16442 (2019)
1T−MoS_2 QS/$Ni(OH)_2$	0.37 S^{-1}@100 mV	11.5 mF cm^{-2}	Adv. Funct. Mater. **30**, 2000551 (2020)
Ni_2P/MoS_2/N:RGO	12.27 S^{-1}@100 mV	61.7 mF cm^{-2}	Adv. Funct. Mater. **29**, 1809151 (2019)
2D-MoS_2/$Co(OH)_2$	4.31 S^{-1}@200 mV	0.91 mF cm^{-2}	Adv. Mater. **30**, 1801171 (2018)
**$1T_{0.72}$-MoS_2@NiS_2**	**12.78 S^{-1}@300 mV**	**316.2 mF cm^{-2}**	**This work**
**$1T_{0.81}$-MoS_2@Ni_2P**	**10.93 S^{-1}@300 mV**	**359.7 mF cm^{-2}**	**This work**

Supplementary Note 2

Calculation for Faradaic efficiency: a glass-sealed gas circulation system (Labsolar 6A, Perfect Light, China) equipped with a three electrode H-type electrochemical cell was used to collect the gas product. The product was subsequently detected by a thermal conductivity detector (TCD) in Agilent 7890B gas chromatography (GC). The as-prepared $1T_{0.72}$-MoS_2@NiS_2 and $1T_{0.81}$-MoS_2@Ni_2P electrodes were directly used as the working electrode (1.0 cm^2). The electrochemical experiments were performed at a constant applied current density of 10 mA cm^{-2}. The electrolyte was 1.0 M KOH and 0.5 M H_2SO_4. GC data was collected for 120 min at the same intervals. The faradaic efficiency was calculated by the following equation[2]: FE% = 2nF/Q.

Where F and n are the faraday constant and the amount of produced hydrogen, respectively; Q is the total amount of charge flowed past the electrochemical cell.

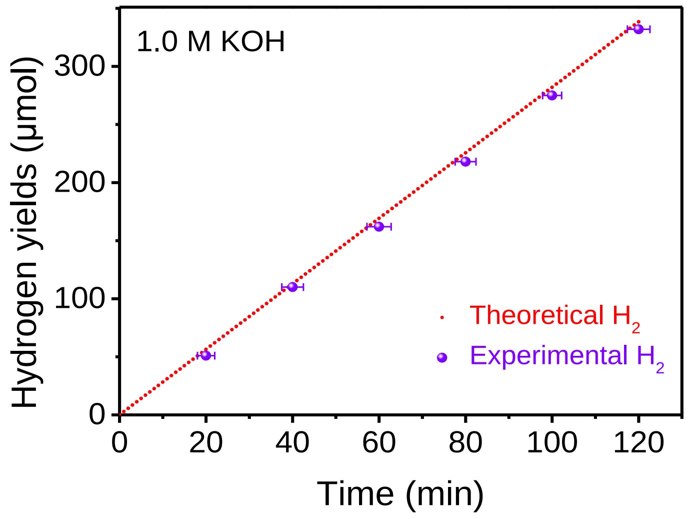

Supplementary Fig. 19. The yields of hydrogen theoretically calculated from HER current and tested from an online gas chromatography system by use of as-prepared $1T_{0.72}$-MoS_2@NiS_2 electrode at a current density of 10 mA cm^{-2} in 1.0 M KOH electrolyte. Error bars indicate the average time which takes for each sample to measure three times to produce the same volume of H_2.

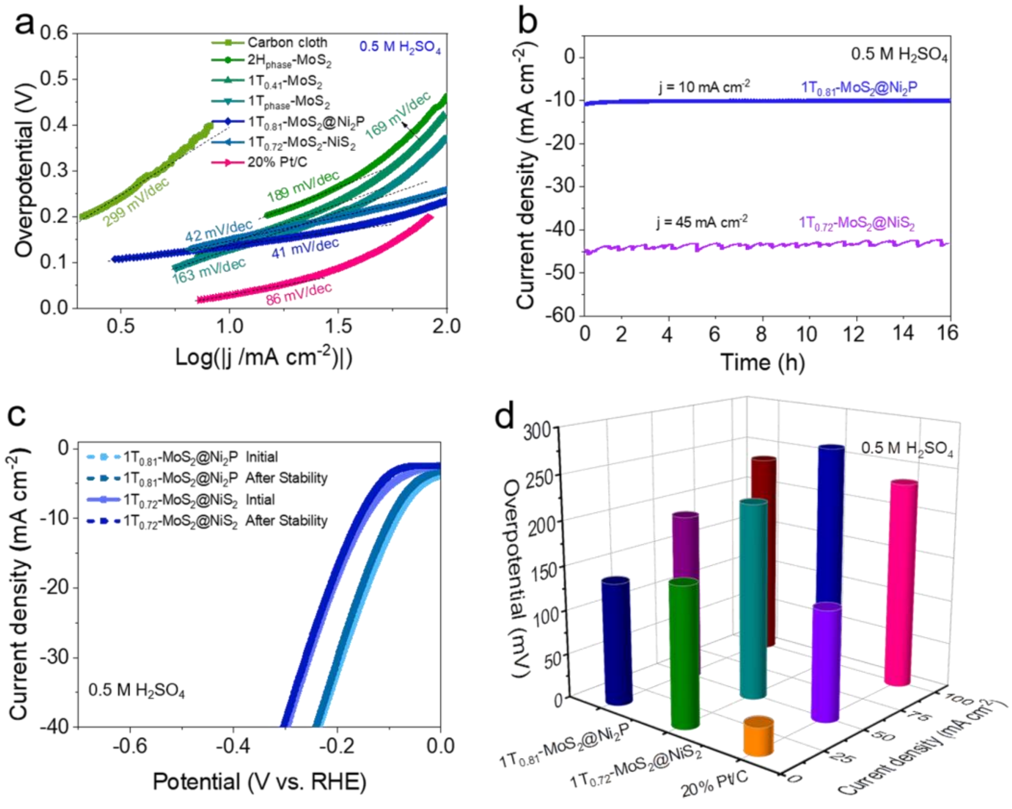

Supplementary Fig. 20. (a) Tafel plots in 0.5 M H_2SO_4. (b) Plot of current density versus time at constant overpotential for $1T_{0.72}$-MoS_2@NiS_2 and $1T_{0.81}$-MoS_2@Ni_2P in 0.5 M H_2SO_4 electrolyte for 16 h, showing its excellent stability performance during the continuous tests. (c) Durability of $1T_{0.72}$-MoS_2@NiS_2 and $1T_{0.81}$-MoS_2@Ni_2P. (d) Overpotentials at various current densities of 20.0 wt% Pt/C, $1T_{0.72}$-MoS_2@NiS_2, and $1T_{0.81}$-MoS_2@Ni_2P in 0.5 M H_2SO_4.

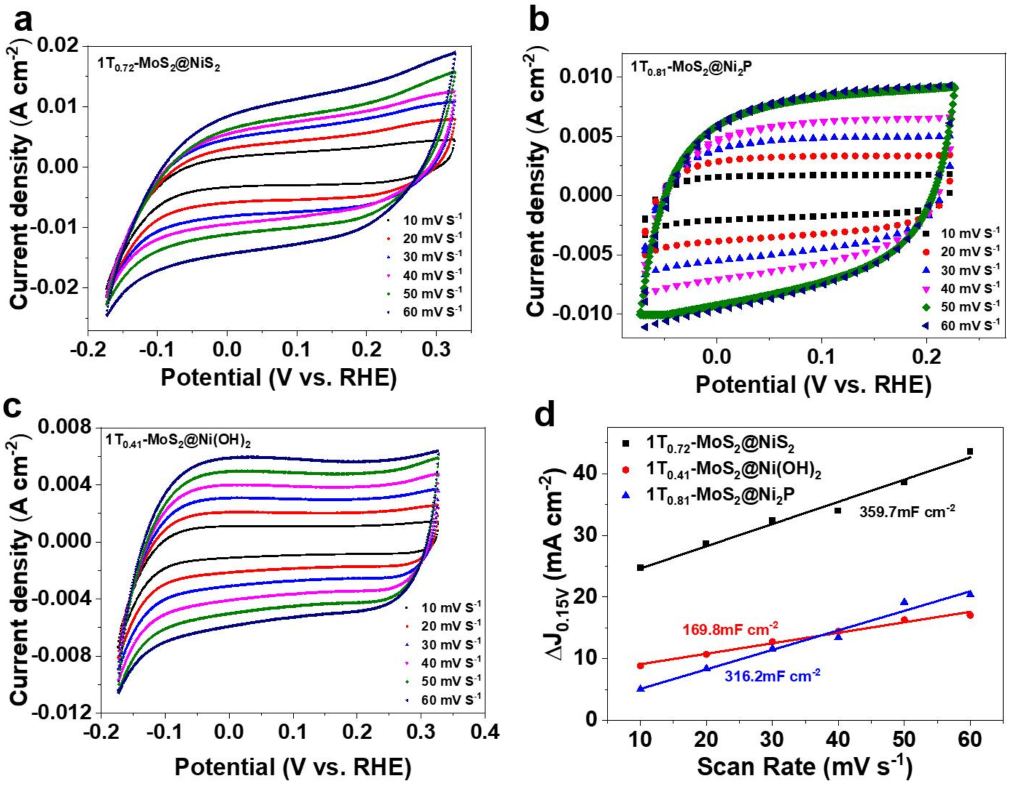

Supplementary Fig. 21. CV curves for $1T_{0.72}$-MoS_2@NiS_2 (a), $1T_{0.81}$-MoS_2@Ni_2P (b), and $1T_{0.41}$-MoS_2@$Ni(OH)_2$ (c) different rates from 10 to 50 mV S^{-1} in the region of −0.6~0.1 V in 0.5M H_2SO_4. (d) the capacitive current at 0.15 V as a function of the scan rate.

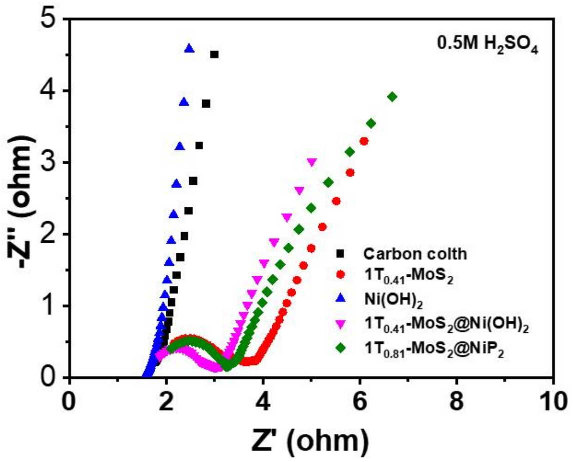

Supplementary Fig. 22. Nyquist plots of carbon cloth, $Ni(OH)_2$, $1T_{0.41}$-MoS_2@$Ni(OH)_2$, $1T_{0.41}$-MoS_2 and $1T_{0.81}$-MoS_2@Ni_2P in 0.5 M H_2SO_4.

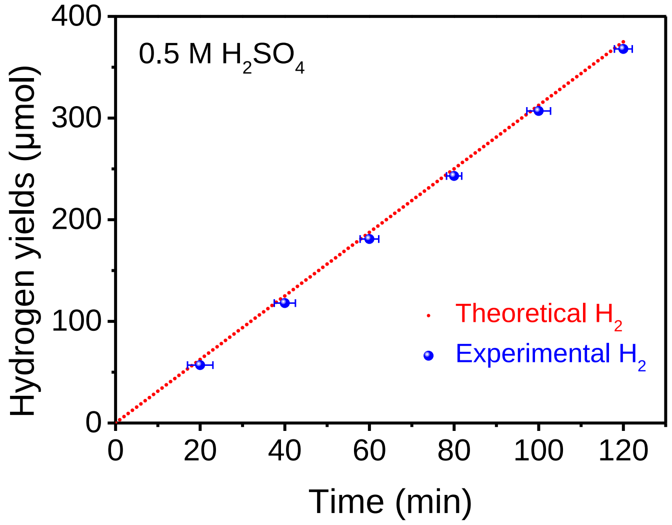

Supplementary Fig. 23. The yields of hydrogen theoretically calculated from HER current and tested from an online gas chromatography system by use of as-prepared $1T_{0.81}$-MoS_2@Ni_2P electrode at a current density of 10 mA cm^{-2} in 0.5 M H_2SO_4 electrolyte. Error bars indicate the average time which takes for each sample to measure three times to produce the same volume of H_2.

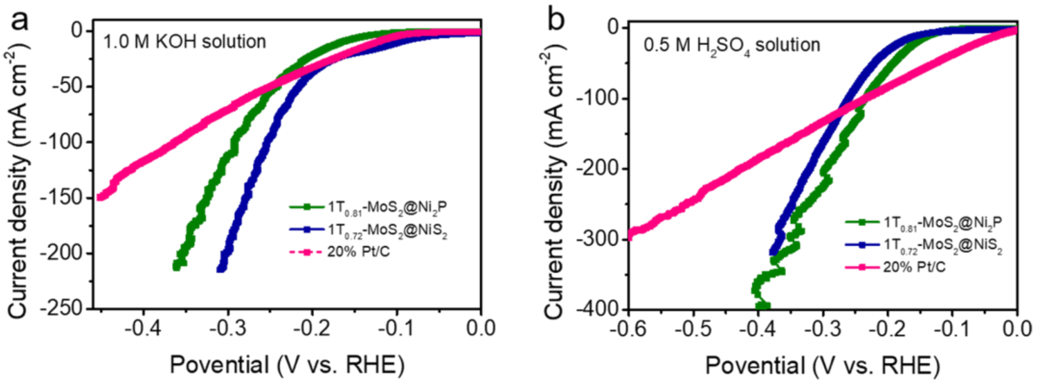

Supplementary Fig. 24. **a** The HER polarization curves of $1T_{0.81}$-MoS_2@Ni_2P, $1T_{0.72}$-MoS_2@NiS_2 and 20% Pt/C electrodes at 5.0 mV s^{-1} in 1.0 M KOH solution. **b** The HER polarization curves of $1T_{0.81}$-MoS_2@Ni_2P, $1T_{0.72}$-MoS_2@NiS_2 and 20% Pt/C electrodes at 5.0 mV s^{-1} in 0.5 M H_2SO_4 solution. (All polarization curves were corrected without iR-compensation).

Supplementary Table 3. Comparison of HER activities of $1T_{0.81}$-MoS_2@Ni_2P, and $1T_{0.72}$-MoS_2@NiS_2 electrocatalysts with the other reported electrocatalysts in 1.0 M KOH electrolyte.

Catalysts	Loadings (mg/cm^2)	η_{10} (mV)	η_{40} (mV)	Tafel slope (mV/dec)	Reference
**$1T_{0.72}$-MoS_2@NiS_2**	**0.685**	**95**	**227**	**68**	**This work**
**$1T_{0.81}$-MoS_2@Ni_2P**	**0.762**	**170**	**284**	**79**	**This work**
1T/2H-MoS_2	0.28	285	380	65	Adv. Energy Mater. **2018**, *8*, 1801345
Edge-rich 1T−MoS_2 QS/$Ni(OH)_2$	0.16	57	78	30	Adv. Funct. Mater. **2020**, 2000551
Ni_2P/MoS_2/N:RGO	0.53	149	225	39.5	Adv. Funct. Mater. **2019**, *29*, 1809151
MoS_2/NiS_2	-	62	105	50.1	Adv. Sci. **2019**, *6*, 1900246
2D-MoS_2/$Co(OH)_2$	0.285	125	186	76	Adv. Mater. **2018**, *30*, 1801171
2D-MoS_2/$Ni(OH)_2$	0.285	185	246	73	Adv. Mater. **2018**, *30*, 1801171
CoS-$Co(OH)_2$@MoS_{2-x}	0.2	143	-	68	Adv. Func. Mat., **2016**, 26(41): 7386-7393.
Ni_2P/MoS_2/N:CNT	0.526	152.1	224	65.54	Adv. Funct. Mater. **2019**, *29*, 1809151
Ni_2P/MoS_2	0.526	159	312	69.46	Adv. Funct. Mater. **2019**, *29*, 1809151
2D 1T–MoS_2	0.285	351	-	156	Adv. Mater. **2018**, 30, 1801171
MoS_2/Ni_3S_2	9.7	110	175	83	Angew. Chem. Int. Ed. **2016**, 55, 6702 –6707
Ni NP|Ni–N–C/EG	0.24	147	-	114	Energy Environ. Sci., **2019**, 12, 149-156
NiFe LDH-NS@G10	0.283	283	-	110	Adv. Mater., **2017**, 29(17): 1700017.
CoS/MoS_2/C	0.182	214	-	106	J. Mater. Chem. A, **2017**, 5, 25410-25419
Ni−Mo_xC/NC	0.86	161	246	104.8	ACS Appl. Mater. Interfaces, **2018**, 10, 35025−35038
Mo_2N–Mo_2C/HGr-3	0.337	154	256	68	Adv. Mater. **2018**, *30*, 1704156
MoP/MoS_2	2.50	98	146	63	ACS Appl. Mater. Interfaces **2019**, 11, 25986−25995
MoS_2/SnS_2	0.204	285	368	109	Nano Energy, **2019**, 64, 103918
(Ni, Fe)S_2@MoS_2	-	130	241	101.22	Appl. Catal. B: Environ., **2019**, 247, 107–114
Ni_2P-NiP_2 HNPs/NF	-	59.7	108	58.8	Adv. Mater. **2018**, 30, 1803590

Supplementary Table 4. Comparison of HER activities of $1T_{0.81}$-MoS_2@Ni_2P, and $1T_{0.72}$-MoS_2@NiS_2 electrocatalysts with the other typical electrocatalysts in 0.5 M H_2SO_4 electrolyte.

Catalysts	Loadings (mg/cm^2)	η_{10} (mV)	η_{40} (mV)	Tafel slope (mV/dec)	Reference
**$1T_{0.72}$-MoS_2@NiS_2**	**0.685**	**138**	**302**	**42**	**This work**
**$1T_{0.81}$-MoS_2@Ni_2P**	**0.762**	**38.9**	**282**	**41**	**This work**
(N, PO_4^{3-})-MoS_2/VG	-	85	118	42	Angew. Chem. Int. Ed. **2019**, 131, 16435 – 16442
Ni_2P/MoS_2/N:RGO	0.526	40	112	39.52	Adv. Funct. Mater. **2019**, *29*, 1809151
Ni_2P/MoS_2/N:CNT	0.526	93.9	152	57.8	Adv. Funct. Mater. **2019**, *29*, 1809151
Ni_2P/MoS_2	0.526	92	378	87.69	Adv. Funct. Mater. **2019**, *29*, 1809151
Mo_2N–Mo_2C/HGr-3	0.337	157	253	55	Adv. Mater. **2018**, *30*, 1704156
MoP/MoS_2	2.50	105	192	79	ACS Appl. Mater. Interfaces **2019**, 11, 25986−25995
MoS_x/NCNT	0.102	110	-	40	Nano Lett. **2014**, 14, 1228-1233
S vacancy MoS_2	-	170	-	60	Nat. Mater., **2016**, 15, 48-53
Metallic MoS_2	0.043	175	-	41	Nat. Commun., **2016**, 7, 10672
CoP/Ni_5P_4/CoP /Ni foam	-	33	-	43	Energy Environ. Sci.,**2018**, 11, 2246-2252
NiMo@NCNT	2.0	65	-	67	ACS Nano, **2016**, 10, 10397-10403
MoP/SN-G	0.5	99	-	54.41	ACS Catal. **2017**, 7, 3030-3038
WS_2/W_2C	0.21	126	-	68	ACS Appl. Energy Mater. **2018**, 1, 3377−3384
MoS_2/SnS_2	0.204	248	350	150	Nano Energy, **2019**, 64, 103918

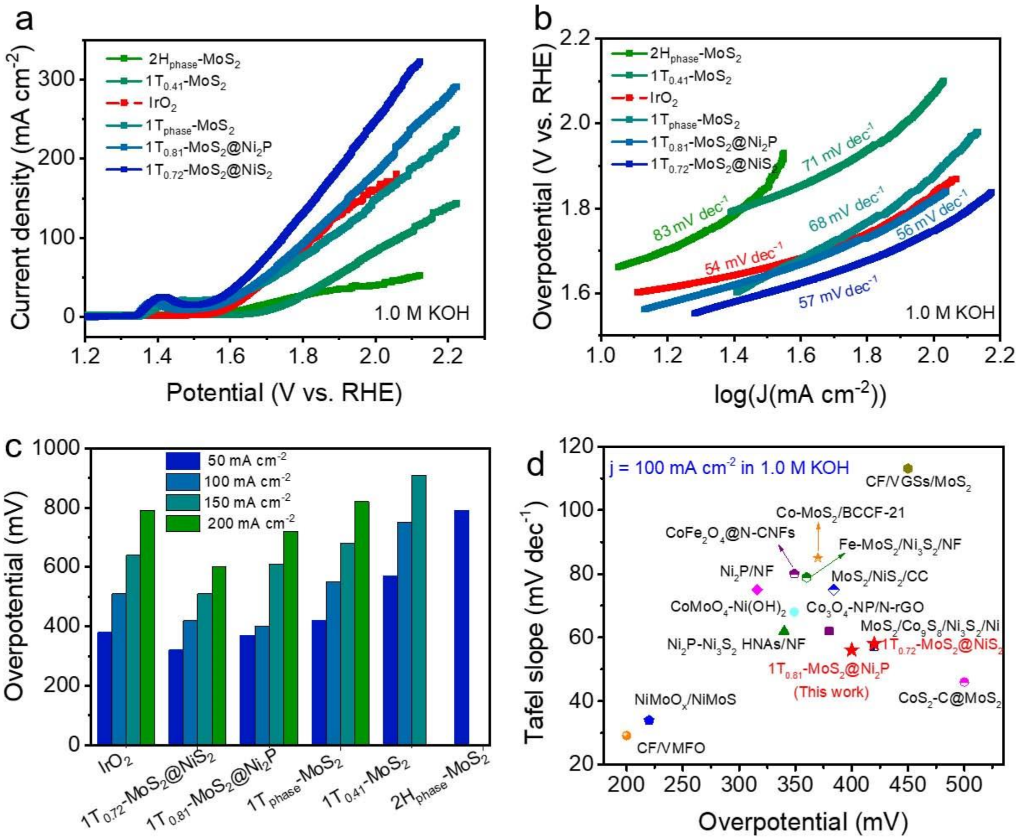

Supplementary Fig. 25. **OER performance of different samples tested in 1.0 M KOH**. **a** OER polarization curves, **b** Tafel slopes, **c** overpotentials at typical current densities of $2H_{phase}$-MoS_2, $1T_{0.41}$-MoS_2, $1T_{phase}$-MoS_2, $1T_{0.72}$-MoS_2@NiS_2, $1T_{0.81}$-MoS_2@Ni_2P and IrO_2. **d** η_{100} and Tafel slopes for various transition metal-based HER electrocatalysts in 1.0 M KOH. (All LSV curves were corrected without iR-compensation).

Supplementary Note 3

Electrocatalytic OER performance. In general, the efficiency is always limited by OER as major barrier for overall water splitting. The OER performance of our catalysts was investigated in 1.0 M KOH solution in a three-electrode system. The polarization curves of different samples are shown in Supplementary Fig. 25a, $1T_{0.72}$-MoS_2@NiS_2 sample exhibits the best OER performance among all samples. Interestingly, OER activities of $1T_{0.72}$-MoS_2@NiS_2 and $1T_{0.81}$-MoS_2@Ni_2P are better than commercial IrO_2 catalyst. Especially, the as-prepared $1T_{0.72}$-MoS_2@NiS_2 (or $1T_{0.81}$-MoS_2@Ni_2P) sample presents the low overpotentials of 320 (370), 420 (400), and 510 (610) mV at current densities of 50, 100, and 150 mA cm^{-2} towards OER, which are better than others and commercial IrO_2 catalyst (Supplementary Fig. 25c), compared with $1T_{phase}$-MoS_2 (420, 550, 680 mV), $1T_{0.41}$-MoS_2 (570, 750, 910 mV), $2H_{phase}$-MoS_2 (790 mV @50 mA cm^{-2}) and IrO_2 (380, 510, 790 mV). To in-depth understand the OER kinetic mechanism, we calculated the Tafel slopes of these electrodes using the Tafel equation [11, 12] and obtained the smallest slopes equal to 56 and 57 mV/dec for the electrodes containing $1T_{0.72}$-MoS_2@NiS_2 and $1T_{0.81}$-MoS_2@Ni_2P, respectively (Supplementary Fig. 25b). These values are even closer to the corresponding slope of the IrO_2 electrode (54 mV/dec), demonstrating the fast OER kinetics of $1T_{0.72}$-MoS_2@NiS_2 and $1T_{0.81}$-MoS_2@Ni_2P electrodes. It can be also seen that the present $1T_{0.72}$-MoS_2@NiS_2 possesses excellent catalytic activities towards OER with smaller overpotential and Tafel slope than most of the previous reports (Supplementary Fig. 25d and Supplementary Table 5). In addition, the

$1T_{0.72}$-MoS_2@NiS_2 and $1T_{0.81}$-MoS_2@Ni_2P have excellent stability. As shown in Supplementary Fig. 26 (a, c), there is only a slight decrease for the current density after testing for 16 h at 20 mA cm^{-2}, and the LSV curves measured before and after the long-term tests are also a slight drop (Supplementary Fig. 26 (b, d)). This may be due to the slight shedding of phosphide or sulfide in our catalyst during the electrochemical reaction of the alkaline medium.

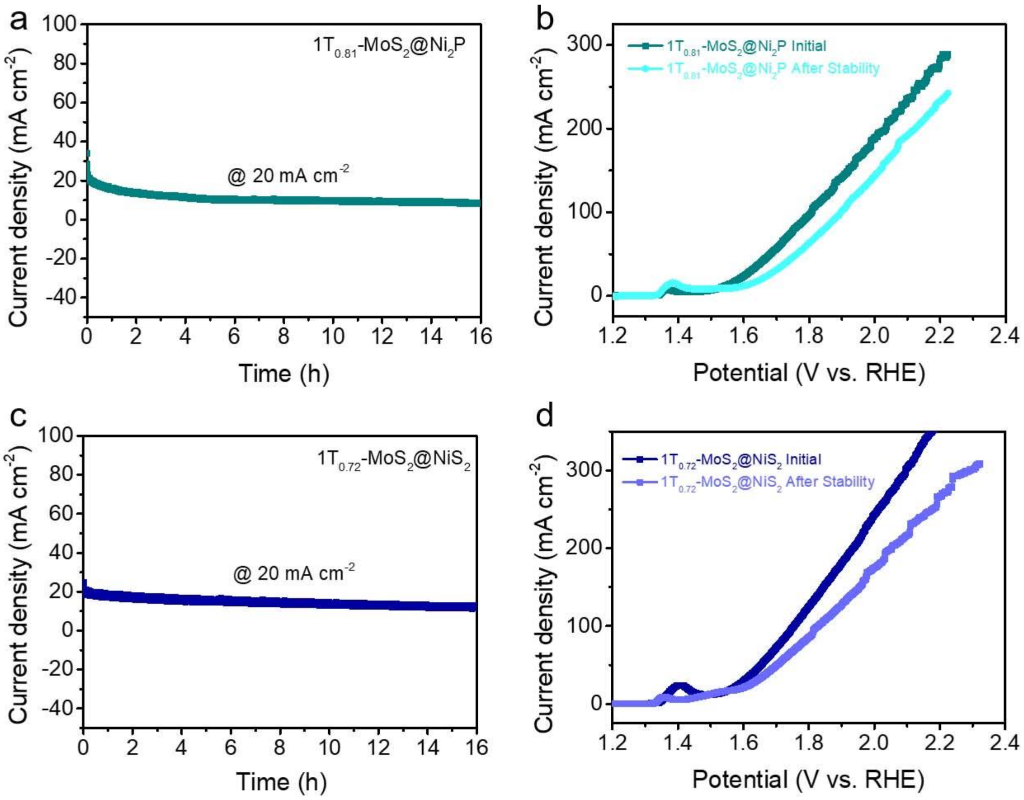

Supplementary Fig. 26. **a** Plot of current density versus time at constant overpotential for $1T_{0.81}$-MoS_2@Ni_2P in 1.0 M KOH electrolyte for 16 h, showing its excellent stability performance during continuous tests. **b** LSV curves of durability of $1T_{0.81}$-MoS_2@Ni_2P. **c** Plot of current density versus time at constant overpotential for $1T_{0.72}$-MoS_2@NiS_2 in 1.0 M KOH electrolyte for 16 h, showing its excellent stability performance during continuous tests. **d** LSV curves of $1T_{0.72}$-MoS_2@NiS_2 for the durability test. (All LSV curves were corrected without iR-compensation).

Supplementary Table 5. Comparison of OER activities for various electrocatalysts.

**Catalysts**	**Overpotential @100 mA/cm^2 (mV)**	**Tafel slope (mV/dec)**	**Reference**
$NiMoO_x$/NiMoS	220	34	Nat. Commun. **11**, 5462 (2020).
$MoS_2/Co_9S_8/Ni_3S_2$/Ni	420	58	J. Am. Chem. Soc. **141**, 10417-10430 (2019)
Ni_2P-Ni_3S_2 HNAs/NF	340	62	Nano Energy **51**, 26-36 (2019)
Ni_2P/NF	316	75	Nano Res. **13**, 2098-2105 (2020)
$CoMoO_4$-$Ni(OH)_2$	349	68	ACS Sustainable Chem. Eng. **6**, 16086 (2018)
Co_3O_4-NP/N-rGO	380	62	Adv. Energy Mater. **8**, 1702222 (2018)
$CoFe_2O_4$@N-CNFs	349	80	Adv. Sci. **4**, 1700226 (2017)
CF/VGSs/MoS_2	450	113.1	Nat. Commun. **12**, 1380 (2021)
CF/VMFO	200	29.9	Nat. Commun. **12**, 1380 (2021)
$(MoS_2)_{0.125}Mo_2C$	~	209	Adv. Mater. Interface **6**, 1900948 (2019)
Fe-MoS_2/Ni_3S_2/NF	360	78.9	Dalton Trans. **48**, 12186-12192 (2019)
CoS_2-C@MoS_2	500	46	ACS Sustainable Chem. Eng. **7**, 2899 (2019)
Co-MoS_2/BCCF-21	370	85	Adv. Mater. **30**, 1801450 (2018)
MoS_2/NiS_2/CC	384	75	Electrochim. Acta **385**, 138438 (2021)
**$1T_{0.81}$-MoS_2@Ni_2P**	**400**	**56**	**This work**
**$1T_{0.72}$-MoS_2@NiS_2**	**420**	**57**	**This work**

Supplementary Note 4

Electrocatalytic performance for overall water splitting. Based on the above results, the overall water splitting measurement was carried out in a standard two-electrode system by using $1T_{0.81}$-MoS_2@Ni_2P (or $1T_{0.72}$-MoS_2@NiS_2) heterostructure catalyst as cathode and $1T_{0.81}$-MoS_2@Ni_2P (or $1T_{0.72}$-MoS_2@NiS_2) catalyst as anode in alkaline medium (1.0 M KOH solution), respectively. For comparison purposes, CC@IrO_2(+)//CC@Pt/C(−) was also prepared and tested in 1.0 M KOH solution. Supplementary Fig. 27a shows the polarization curves of the above-mentioned two water-splitting electrolyzers. It was found that the $1T_{0.72}$-MoS_2@NiS_2(+)//$1T_{0.72}$-MoS_2@NiS_2(−) and $1T_{0.81}$-MoS_2@Ni_2P(+)//$1T_{0.81}$-MoS_2@Ni_2P(−) cells could require a cell voltage of 1.62 V and 1.86V at a current density of 20 mA·cm^{-2}, respectively, while the voltages of 1.92 V is required for the IrO_2(+)//Pt/C(−) cell. Clearly, the overall water splitting performance of as-prepared catalysts is better than that of IrO_2(+)//Pt/C(−), especially at high current density. The water splitting potential of 1.62 V at the current density of 20 mA cm^{-2} is smaller than those of most reported electrocatalysts, such as MoS_2/NiS_2(+)//MoS_2/NiS_2(−)(1.63 V)[13], MoS_2/NiS NCs(+)//MoS_2/NiS NCs(−)(1.71 V)[14], MoS_2/Co_9S_8/Ni_3S_2(+)//MoS_2/Co_9S_8/Ni_3S_2(+)(1.70 V)[15], Fe-Ni@NC-CNFs(+) //Fe-Ni@NC-CNFs(−)(1.88 V)[16], NC/NiMo/$NiMoO_x$(+)//NC/NiMo/$NiMoO_x$(−)(1.64 V)[17], and N-$NiMoO_4$/NiS_2(+)//N-$NiMoO_4$/NiS_2(−)(1.70 V)[18] (Supplementary Table 6). Currently, most reported water-splitting electrocatalysts require voltages higher than 1.62 V to reach 20 mA cm^{-2} (with iR-compensation). It should be noticed that our

OER electrocatalyst $1T_{0.72}$-MoS_2@NiS_2 is much better than the HER electrocatalyst $1T_{0.81}$-MoS_2@Ni_2P. The working stability of our electrocatalysts during overall water splitting was tested (Supplementary Fig. 27b), which indicates that the voltage keeps almost unchanged after 16 h at the current densities of 10 mA cm^{-2}, indicating excellent stability.

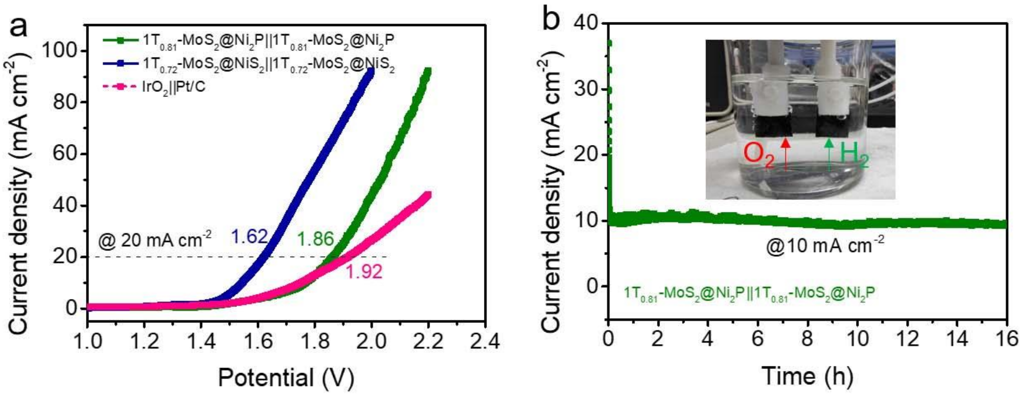

Supplementary Fig. 27. Overall water splitting activity of the samples in 1.0 M KOH solution. **a** Polarization curves of $1T_{0.81}$-MoS_2@Ni_2P(+)//$1T_{0.81}$-MoS_2@Ni_2P(−), $1T_{0.72}$-MoS_2@NiS_2(+)//$1T_{0.72}$-MoS_2@NiS_2(−) and IrO_2//Pt/C at a scan rate of 5 mV s^{-1}. **b** Catalytic stability of $1T_{0.81}$-MoS_2@Ni_2P(+)//$1T_{0.81}$-MoS_2@Ni_2P(−) at 10 mA cm^{-2} tested in a two-electrode configuration. (All LSV curves were corrected without iR-compensation).

Supplementary Table 6. Comparison of water-splitting activity of $1T_{0.81}$-MoS_2@Ni_2P(+)//$1T_{0.81}$-MoS_2@Ni_2P(−),$1T_{0.72}$-MoS_2@NiS_2(+)//$1T_{0.72}$-MoS_2@ NiS_2(−) cell in this work with other reported electrocatalysts in 1.0 M KOH solution (V_{20}-cell voltage at 20 mA cm^{-2}). *The data were calculated according to the curves (LSV curves were corrected with iR-compensation) given in the literature.

**Catalysts**	**Support**	**V_{20} (V)**	**Reference**
CF/VMFO(+)‖CF/VMFO(-)	Carbon fibers	1.48*	Nat. Commun. **12**, 1380 (2021)
Cu@NiFe LDH(+)‖Cu@NiFe LDH(-)	Cu foam	1.58*	Energy Environ. Sci. **10**, 1820-1827 (2017)
MoS_2/NiS_2(+)‖MoS_2/NiS_2(-)	Carbon cloth	1.63*	Adv. Sci. **6**, 1900246 (2019)
MoS_2/NiS NCs(+)‖MoS_2/NiS NCs(-)	Ni foam	1.71*	J. Mater. Chem. A **6**, 9833-9838 (2018)
MoS_2/Co_9S_8/Ni_3S_2(+)‖MoS_2/Co_9S_8/Ni_3S_2(+)	Ni foam	1.70*	J. Am. Chem. Soc. **141**, 10417-10430 (2019)
$NiMoO_x$/NiMoS(+)‖$NiMoO_x$/NiMoS(-)	~	1.54*	Nat. Commun. **11**, 5462 (2020)
Ni_2P-Ni_3S_2 HNAs(+)‖Ni_2P-Ni_3S_2 HNAs(-)	Ni foam	1.54*	Nano Energy **51**, 26-36 (2018)
Fe-Ni@NC-CNFs(+)‖Fe-Ni@NC-CNFs(-)	Carbon fibers	1.88*	Angew. Chem. Int. Ed. **57**, 8921-8926 (2018)
Ni@N-C(+)‖Ni@N-C(-)	~	1.78*	Inorg. Chem. **60**, 6764-6771 (2021)
NC/NiMo/$NiMoO_x$(+)‖NC/NiMo/$NiMoO_x$(-)	Ni foam	1.64*	Small **13**, 1702018 (2017)
$CoNi(OH)_x$(+)‖NiN_x(-)	Ni foam	1.73*	Adv. Energy Mater. **6**, 1501661 (2016)
N-$NiMoO_4$/NiS_2(+)‖N-$NiMoO_4$/NiS_2(-)	Ni foam	1.70*	Adv. Funt. Mater. **29**, 1805298 (2019)
Co_xPO_4/CoP(+)‖Co_xPO_4/CoP(-)	~	1.91*	Adv. Mater. **27**, 3175-3180 (2015)
**$1T_{0.81}$-MoS_2@Ni_2P(+)//$1T_{0.81}$-MoS_2@Ni_2P(-)**	**Carbon cloth**	**1.86**	**This work**
**$1T_{0.72}$-MoS_2@NiS_2(+)//$1T_{0.72}$-MoS_2@NiS_2(-)**	**Carbon cloth**	**1.62**	**This work**

Supplementary Note 5

Some phosphides or sulfides fell off from the catalyst in alkaline medium after 16 hours of HER stability test on our samples. Therefore, we performed XPS analysis on the samples after 16 hours of HER stability test. Elemental analysis of the as-prepared catalyst samples before and after the HER stability (16 hours) test, as shown in Supplementary Table 7. Obviously, the contents of Mo, Ni, S, P in $1T_{0.81}$-MoS_2@Ni_2P and $1T_{0.72}$-MoS_2@NiS_2 samples are all decreased after 16 hours of HER stability test (Supplementary Table 7). In contrast, two distinctive peaks Mo $3d_{5/2}$ (228.44 eV) and Mo $3d_{3/2}$ (232.37 eV) for $1T_{0.81}$-MoS_2@Ni_2P sample after HER stability test are negative-shifted of about 0.8 eV (Supplementary Fig. 28), compared with that of the pristine $1T_{0.81}$-MoS_2@Ni_2P sample. With regard to S 2p regions, two peaks of S 2*p*3/2 (161.79 eV) and S 2*p* 1/2 (163.28 eV) are also negative-shifted of about 0.3 eV (Supplementary Fig. 28b). Supplementary Fig. 28c shows the P 2*p* core level XPS spectrum, the peaks located at 129.06 eV and 130.60 eV are due to the Ni-P bonding, whereas the another one of 133.20 eV is associated with the P-O bonding of phosphate. With regard to Ni 2*p* regions, the peaks of Ni $2p_{3/2}$ and the corresponding satellite appear at 855.51 and 861.09 eV, respectively. The peaks of Ni $2p_{1/2}$ and the corresponding satellite appear at 873.47 and 880.50 eV, respectively. These are also negative-shifted of about 0.7 eV and a new peak Ni^0 (851.90 eV) appeared for $1T_{0.81}$-MoS_2@Ni_2P sample after HER stability test, indicating that Ni is reduced during the HER process. For the $1T_{0.72}$-MoS_2@NiS_2 sample after HER stability test, we also obtained similar results (Supplementary Fig. 29). All these results indicate

that electron transfer occurs between Mo and Ni to promote HER activity in the catalyst.

Supplementary Table 7. Elemental analysis (atomic %) of the as-prepared catalyst samples before and after the HER stability (16 hours) test.

**HER**	**$1T_{0.81}$-MoS_2@Ni_2P**				**$1T_{0.72}$-MoS_2@NiS_2**		
	Mo 3d	Ni 2p	S 2p	P 2p	Mo 3d	Ni 2p	S 2p
**Before**	19.29 %	1.78 %	32.70 %	8.32 %	13.96 %	4.39 %	36.96 %
**After**	1.21 %	0.96 %	11.71 %	2.36 %	2.78 %	1.08 %	15.46 %

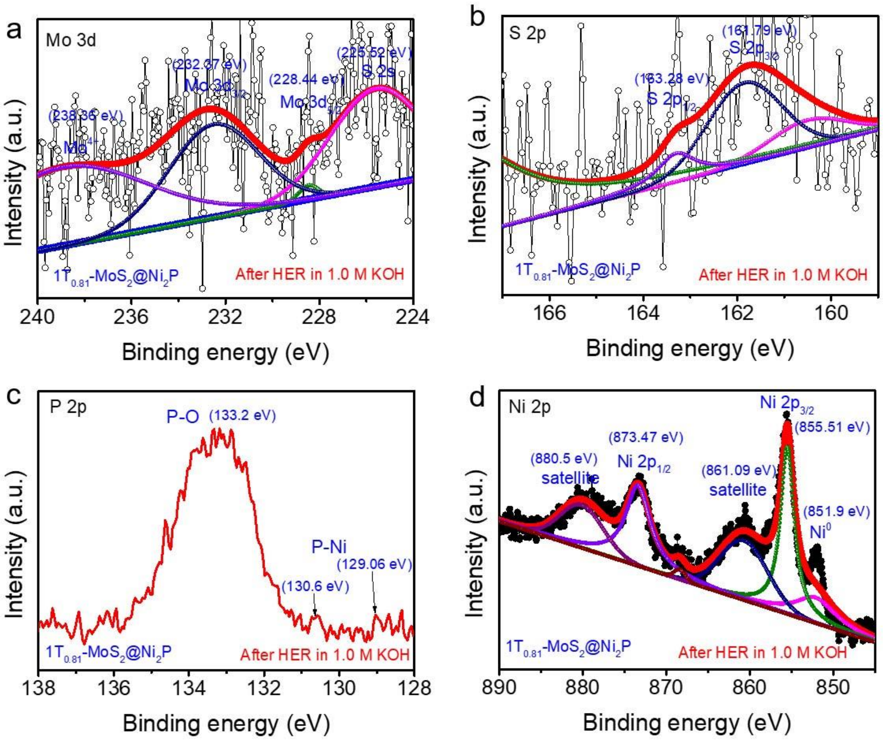

Supplementary Fig. 28. XPS spectra of $1T_{0.81}$-MoS_2@Ni_2P heterostructure catalyst after 16 h stability measurement at 10 mA cm^{-2} in 1.0 M KOH solution. **a** Mo 3*d*, **b** S 2*p*, **c** P 2*p*, **d** Ni 2*p*.

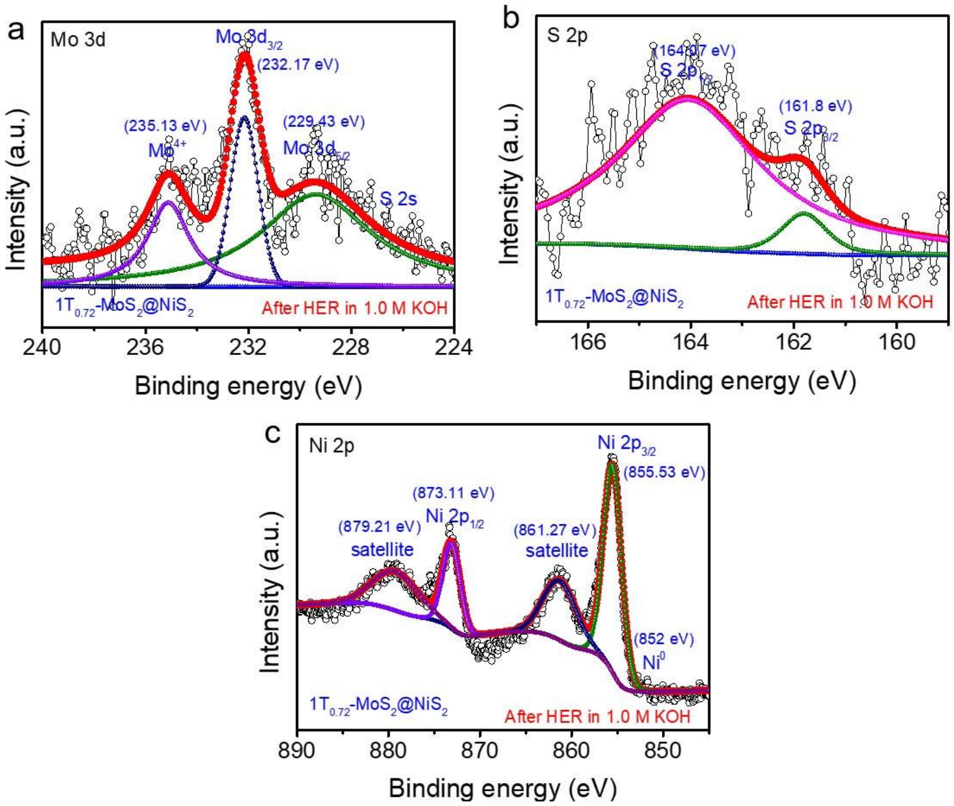

Supplementary Fig. 29. XPS spectra of $1T_{0.72}$-MoS_2@NiS_2 heterostructure catalyst after 16 h stability measurement at 10 mA cm^{-2} in 1.0 M KOH solution. **a** Mo 3*d*, **b** S 2*p*, **c** Ni 2*p*.

Supplementary Note 6

Construction of a stable nanostructured electrocatalyst is of great importance for optimizing the electrochemical performance of HER. The morphologies of the $1T_{0.81}$-MoS_2@Ni_2P and $1T_{0.72}$-MoS_2@NiS_2 catalysts still remain flower-shaped MoS_2 microspheres (shown in Supplementary Fig. 30, SEM image) after the durability measurements, which reveals that the MoS_2 microspheres nanosheet structure can reduce the disintegration tendency during the alternate processes of bubble accumulation and bubble release. In addition, the XRD patterns of the $1T_{0.81}$-MoS_2@Ni_2P and $1T_{0.72}$-MoS_2@NiS_2 microspheres retained the original crystallographic structure after the long-term test (shown in Supplementary Fig. 31). To better understand the activation mechanism, we examined the Raman, and XPS of $1T_{0.81}$-MoS_2@Ni_2P and $1T_{0.72}$-MoS_2@NiS_2 sample before and after the durability measurements. The Raman spectroscopy of the $1T_{0.81}$-MoS_2@Ni_2P shows obviously stronger and blueshifts after the long-term test (Supplementary Fig. 32a), due to strong susceptibility to the influence of electron−phonon coupling. [3] Raman spectra of the $1T_{0.72}$-MoS_2@NiS_2 also do not show any new peaks after HER long-term testing (Supplementary Fig. 32b). All of these confirm the good electrochemical stability of the as-prepared catalysts in the HER process under alkaline and acidic media. In addition, chemical stability during the durability measurements was also detected by XPS (Supplementary Fig. 33-36). For the $1T_{0.81}$-MoS_2@Ni_2P heterostructure catalyst, two characteristic peaks located at 229.54 eV (Mo $3d_{5/2}$) and 233.0 eV (Mo $3d_{3/2}$) of MoS_2 are observed (Supplementary Fig. 33a). After 16 h stability measurement at 10

mA cm^{-2}, the Mo 3*d* spectra change and shift obviously (Supplementary Fig. 34a). The $1T_{0.81}$-MoS_2@Ni_2P is also supported by the XPS measurement, which shows a shift of around +0.3 eV in S 2*p* peaks after 16 h stability measurement (Supplementary Fig. 34b). This shift in binding energy is consistent with what was previously observed at p-doped MoS_2.[4, 5] With regard to Ni 2*p* regions, the peaks of Ni $2p_{3/2}$ and the corresponding satellite appear at 857.4 and 862.8 eV, respectively. The peaks of Ni $2p_{1/2}$ and the corresponding satellite appear at 875.6 and 880.2 eV, respectively. These are also negative-shifted of about 0.8 eV and a new peak Ni^0 (852.5 eV) appeared for $1T_{0.81}$-MoS_2@Ni_2P sample after HER stability test (Supplementary Fig. 34), indicating that Ni is reduced during the HER process. For the $1T_{0.72}$-MoS_2@NiS_2 sample after HER stability test, we also obtained similar results (Supplementary Fig. 35 and Supplementary Fig. 36). All these results indicate that electron transfer occurs between Mo and Ni to promote HER activity in the catalyst.

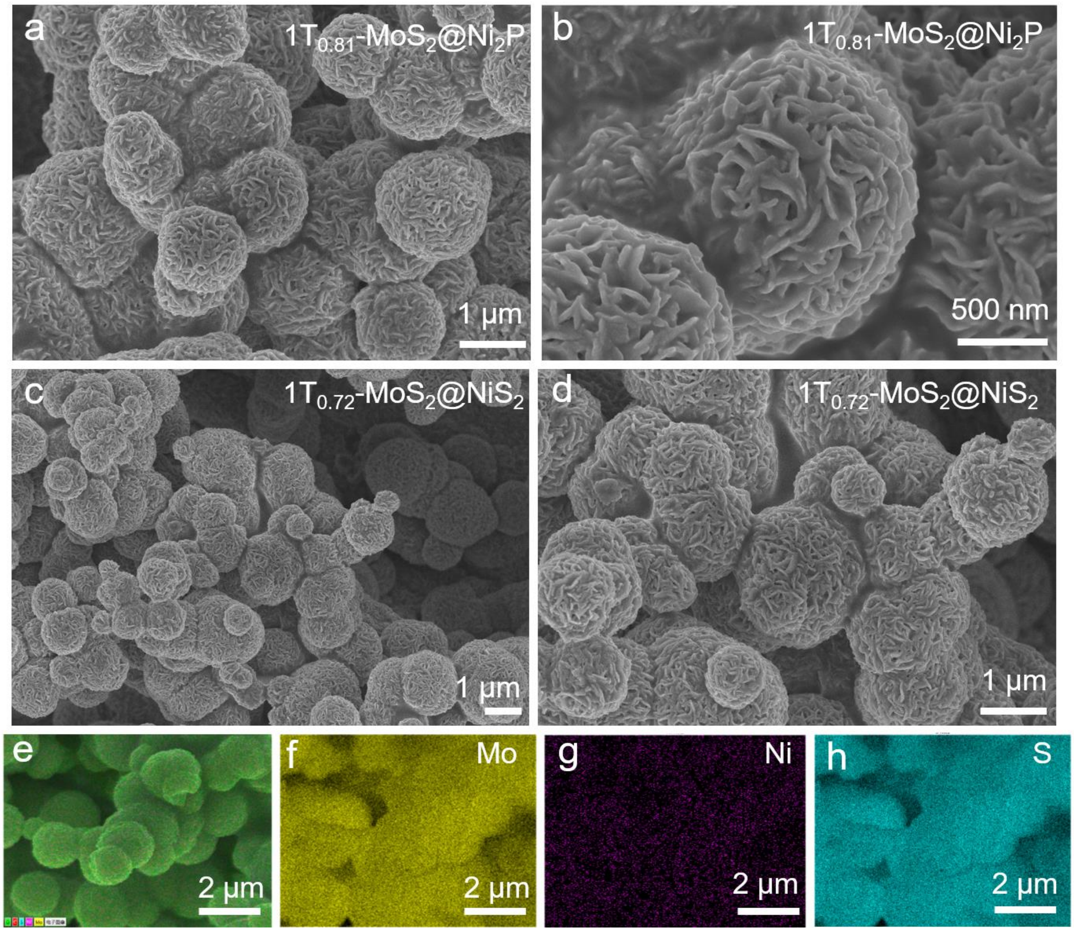

Supplementary Fig. 30. a-b SEM images of $1T_{0.81}$-MoS_2@Ni_2P after 16 h stability measurement at 10 mA cm^{-2}. **c-d** SEM images of $1T_{0.72}$-MoS_2@NiS_2 after 16 h stability measurement at 45 mA cm^{-2}. EDS mapping of $1T_{0.72}$-MoS_2@NiS_2 of the **f** Mo, **g** Ni and **h** S elements, and **e** overlap mapping of elements.

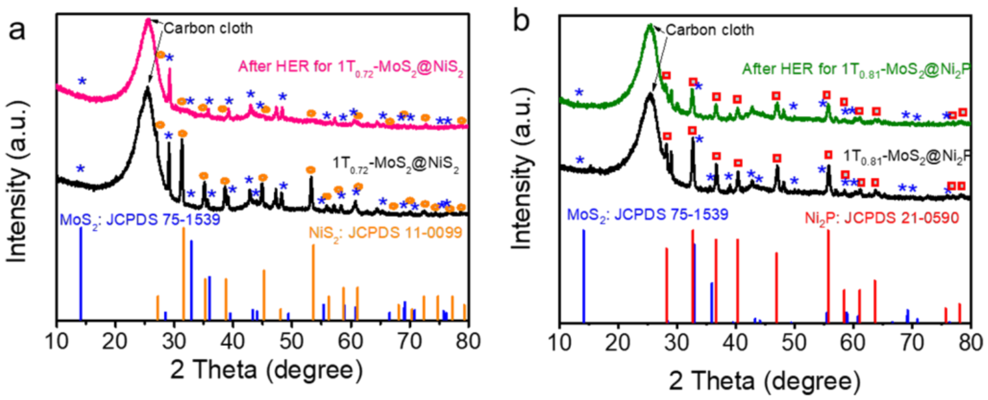

Supplementary Fig. 31. a XRD patterns for $1T_{0.72}$-MoS_2@NiS_2 catalyst before and after 16 h stability measurement at 45 mA cm^{-2} in 0.5 M H_2SO_4 solution. **b** XRD patterns for $1T_{0.81}$-MoS_2@Ni_2P catalyst before and after 16 h stability measurement at 10 mA cm^{-2} in 0.5 M H_2SO_4 solution.

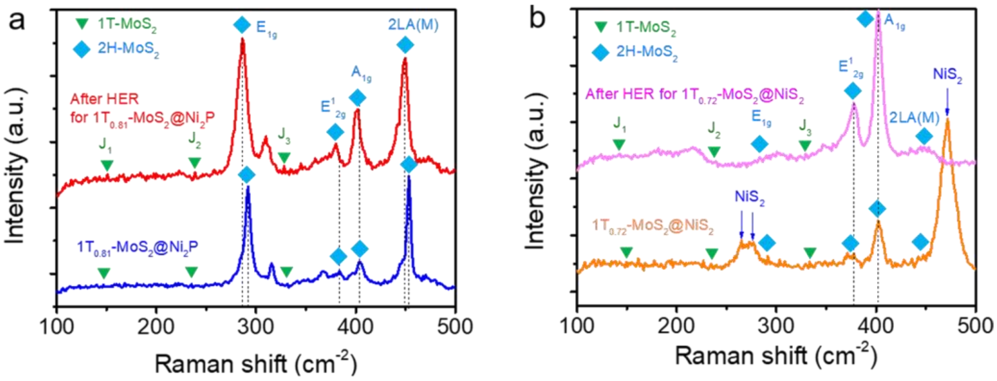

Supplementary Fig. 32. a Raman spectra of $1T_{0.81}$-MoS_2@Ni_2P catalyst before and after 16 h stability measurement at 10 mA cm^{-2} in 0.5 M H_2SO_4 solution. **b** Raman spectra of $1T_{0.72}$-MoS_2@NiS_2 catalyst before and after 16 h stability measurement at 45 mA cm^{-2} in 0.5 M H_2SO_4 solution.

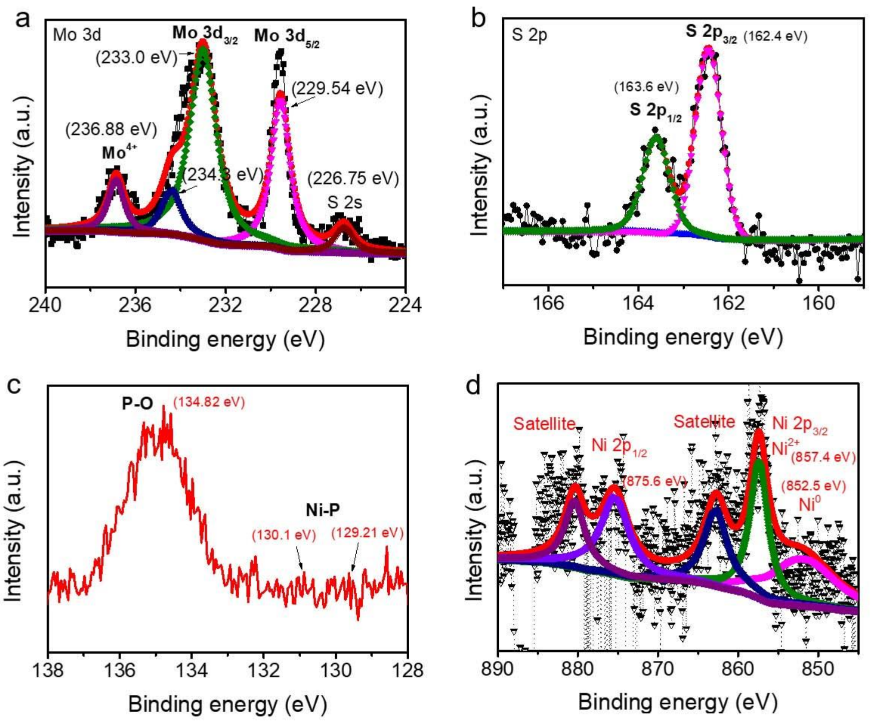

Supplementary Fig. 33. XPS spectra of $1T_{0.81}$-MoS_2@Ni_2P heterostructure catalyst after 16 h stability measurement at 10 mA cm^{-2} in 0.5 M H_2SO_4 solution. **a** Mo 3*d*, **b** S 2*p*, **c** P 2*p*, and **d** Ni 2*p*.

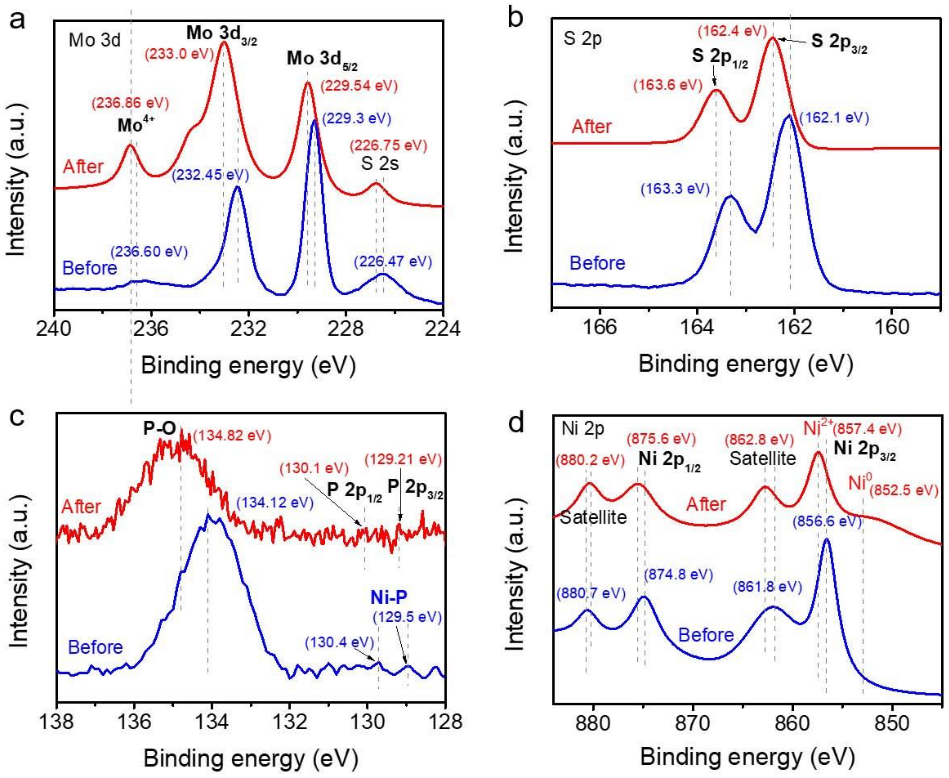

Supplementary Fig. 34. **a** S 2*p*, **b** Mo 3*d*, **c** P 2*p* and **d** Ni 2*p* XPS spectra of $1T_{0.81}$-MoS_2@Ni_2P samples before and after HER cycling in 0.5 M H_2SO_4 solution.

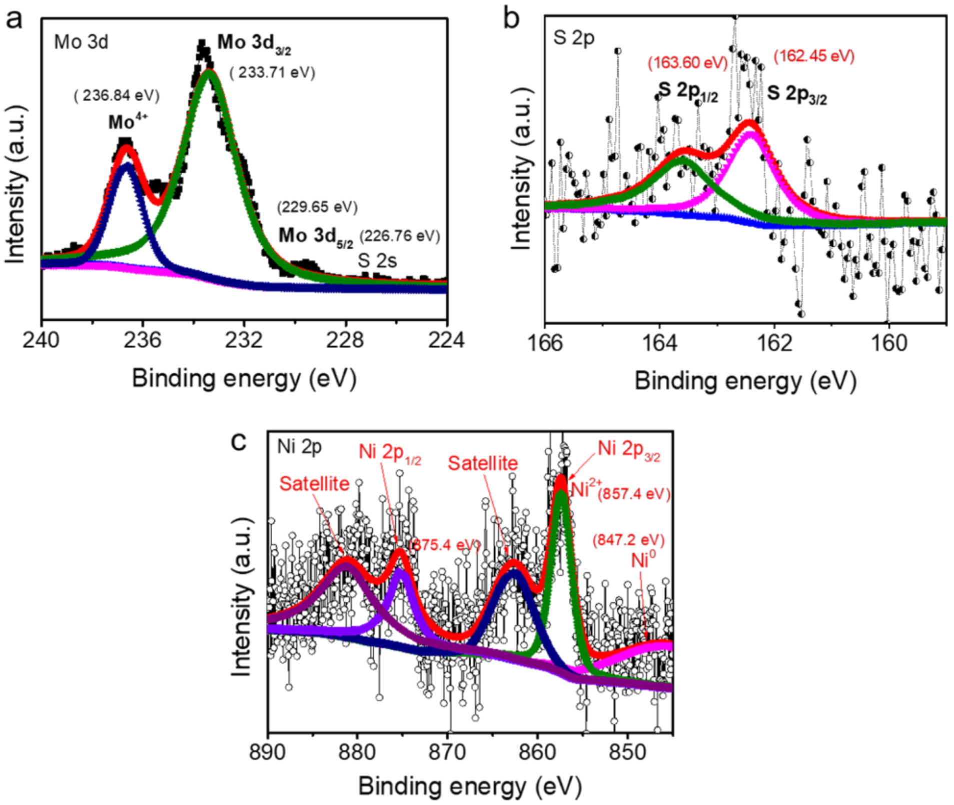

Supplementary Fig. 35. XPS spectra of $1T_{0.72}$-MoS_2@NiS_2 heterostructure catalyst after 16 h stability measurement at 45 mA cm^{-2} in 0.5 M H_2SO_4 solution. **a** Mo 3*d*, **b** S 2*p*, **c** Ni 2*p*.

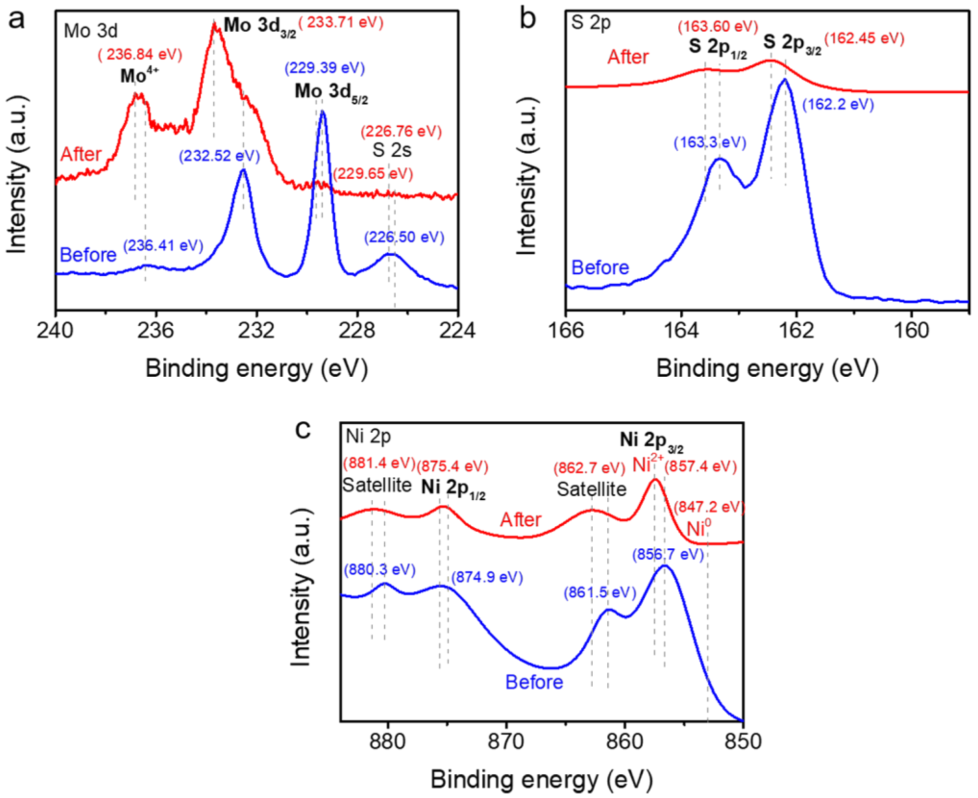

Supplementary Fig. 36. **a** S 2*p*, **b** Mo 3*d*, and **c** Ni 2*p* XPS spectra of $1T_{0.72}$-MoS_2@NiS_2 samples before and after HER cycling in 0.5 M H_2SO_4 solution.

Supplementary Note 7

Computational details:

All theoretical investigations were performed via the VASP code (Vienna Ab initio Simulation Package)[6]. Geometry optimizations were conducted using the highly accurate generalized gradient approximation (GGA) method with the Perdew-Burke-Ernzerhof (PBE) exchange-correlation function[7]. The cutoff energy, k-point, and Zn-atom initial valence-state were set to 570 eV, 6 × 6 × 1, and +2, respectively[8], and the optimal geometry was collected based on the electron-step energy convergence criterion and ion-force convergence criterion of 10^{-6} eV and 0.01 eV/Å, respectively. The LDA + U method proposed by Dudarev[9] was used in the allowing correlation effect of transition metals. The hybrid density function of HSE (Heyd-Scuseria-Ernzerhof)[10] was applied to the calculations to obtain accurate band structures theoretically.

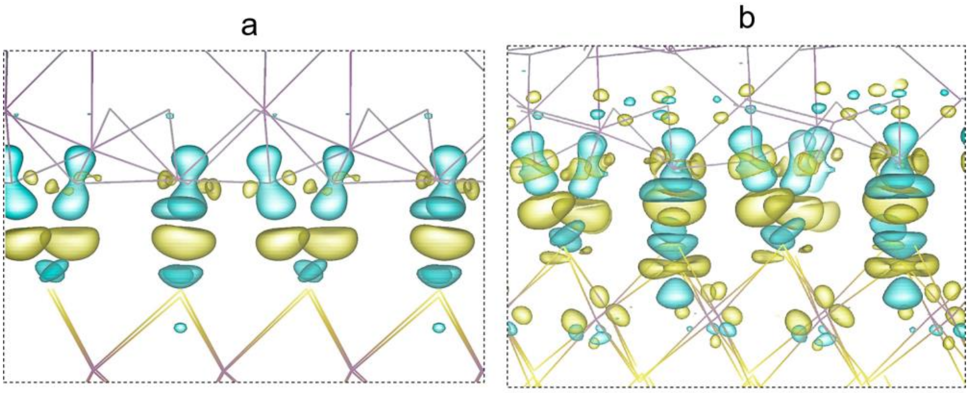

Supplementary Fig. 37. **a** Isosurfaces of local charge-density difference of $1T_{phase}$-MoS_2@Ni_2P interface. yellow and green isosurfaces represent positive and negative spin densities (0.00295308 e/$Å^3$), respectively. **b** Isosurfaces of local charge density difference of $2H_{phase}$-MoS_2@Ni_2P interface. yellow and green isosurfaces represent positive and negative spin densities (0.000509558 e/$Å^3$), respectively.

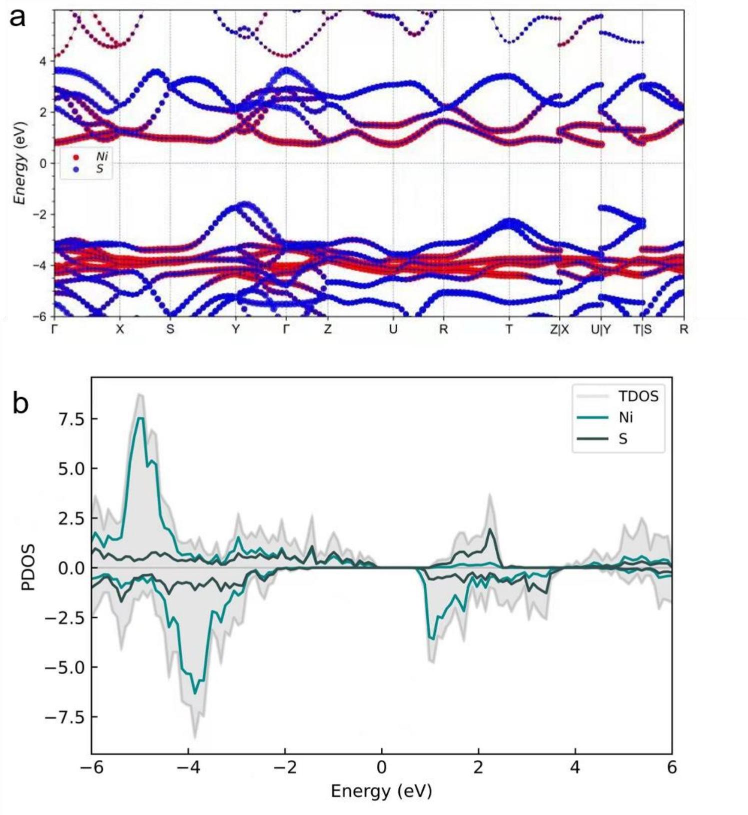

Supplementary Fig. 38. **a** Band structure and **b** spin-polarized density of states (DOS) for NiS_2.

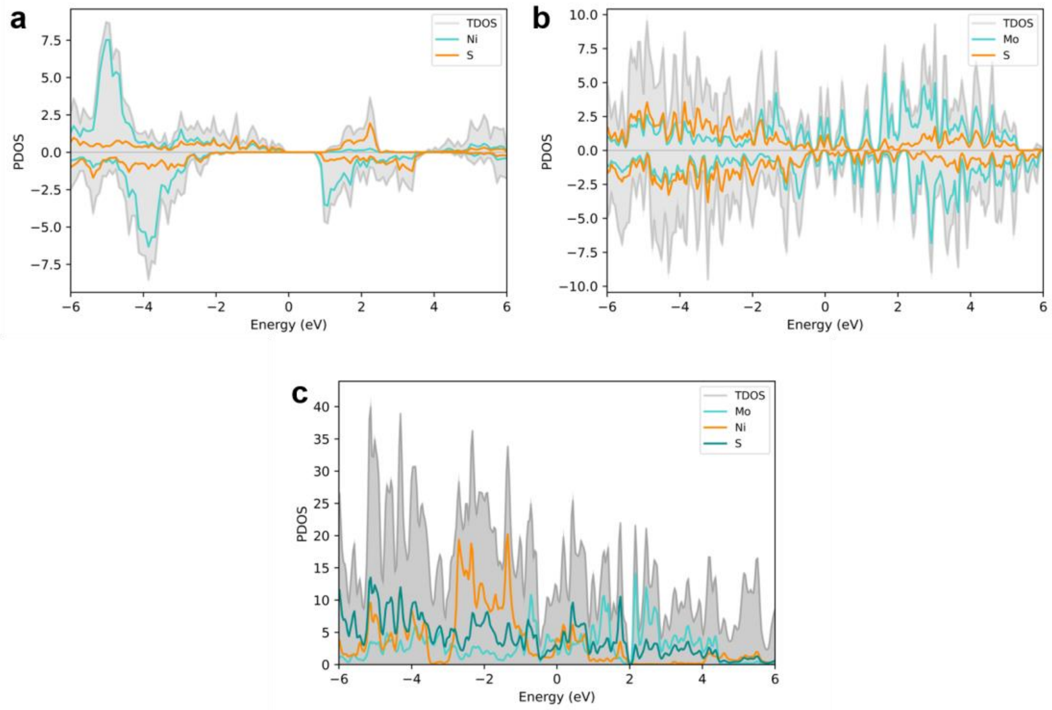

Supplementary Fig. 39. Density of states (DOS) plots of **a** NiS_2, **b** $1T_{phase}$-MoS_2, and **c** $1T_{phase}$-MoS_2@NiS_2 systems.

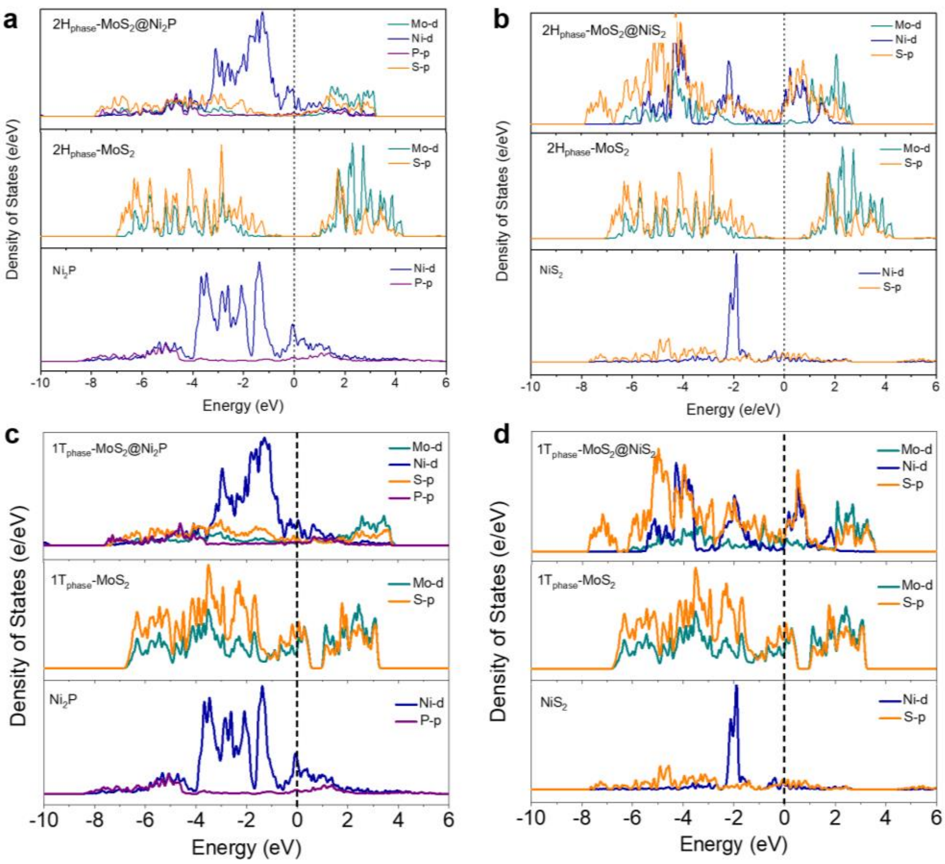

Supplementary Fig. 40. **a** Density of states (DOS) plots of Ni_2P, $2H_{phase}$-MoS_2, and $2H_{phase}$-MoS_2@Ni_2P systems. **b** DOS plots of NiS_2, $2H_{phase}$-MoS_2, and $2H_{phase}$-MoS_2@NiS_2 systems. **c** DOS plots of Ni_2P, $1T_{phase}$-MoS_2, and $1T_{phase}$-MoS_2@Ni_2P systems. **d** DOS plots of NiS_2, $1T_{phase}$-MoS_2, and $1T_{phase}$-MoS_2@NiS_2 systems.

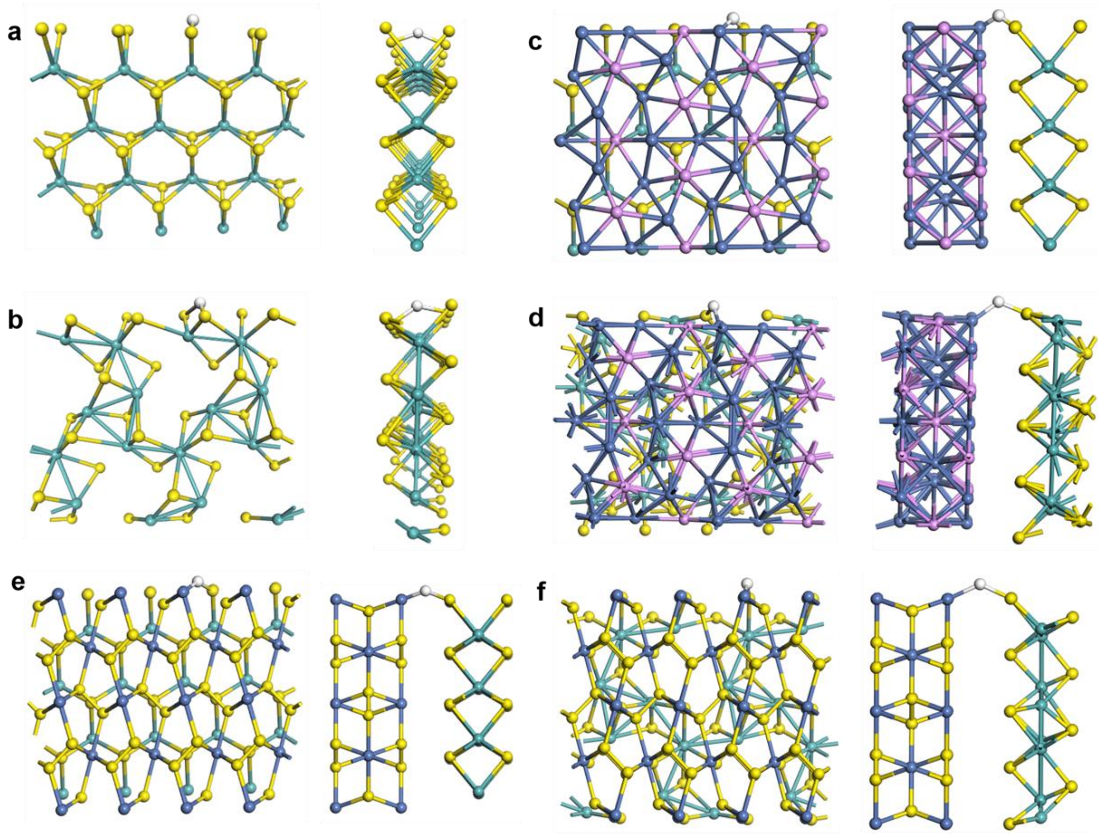

Supplementary Fig. 41. **a** Top and side views of relaxed $2H_{phase}$-MoS_2 (left) and $2H_{phase}$-MoS_2 (right) edges with *H adsorption. **b** Top and side views of relaxed $1T_{phase}$-MoS_2 (left) and $1T_{phase}$-MoS_2 (right) edges with *H adsorption. **c** Top and side views of relaxed $2H_{phase}$-MoS_2@Ni_2P (left) and $2H_{phase}$-MoS_2@Ni_2P (right) edges with *H adsorption. **d** Top and side views of relaxed $1T_{phase}$-MoS_2@Ni_2P (left) and $1T_{phase}$-MoS_2@Ni_2P (right) edges with *H adsorption. **e** Top and side views of relaxed $2H_{phase}$-MoS_2@NiS_2 (left) and $2H_{phase}$-MoS_2@NiS_2 (right) edges with *H adsorption. **f** Top and side views of relaxed $1T_{phase}$-MoS_2@NiS_2 (left) and $1T_{phase}$-MoS_2@NiS_2 (right) edges with *H adsorption. Dark green, yellow, blue, purple and white spheres represent Mo, S, Ni, P and H atoms, respectively.

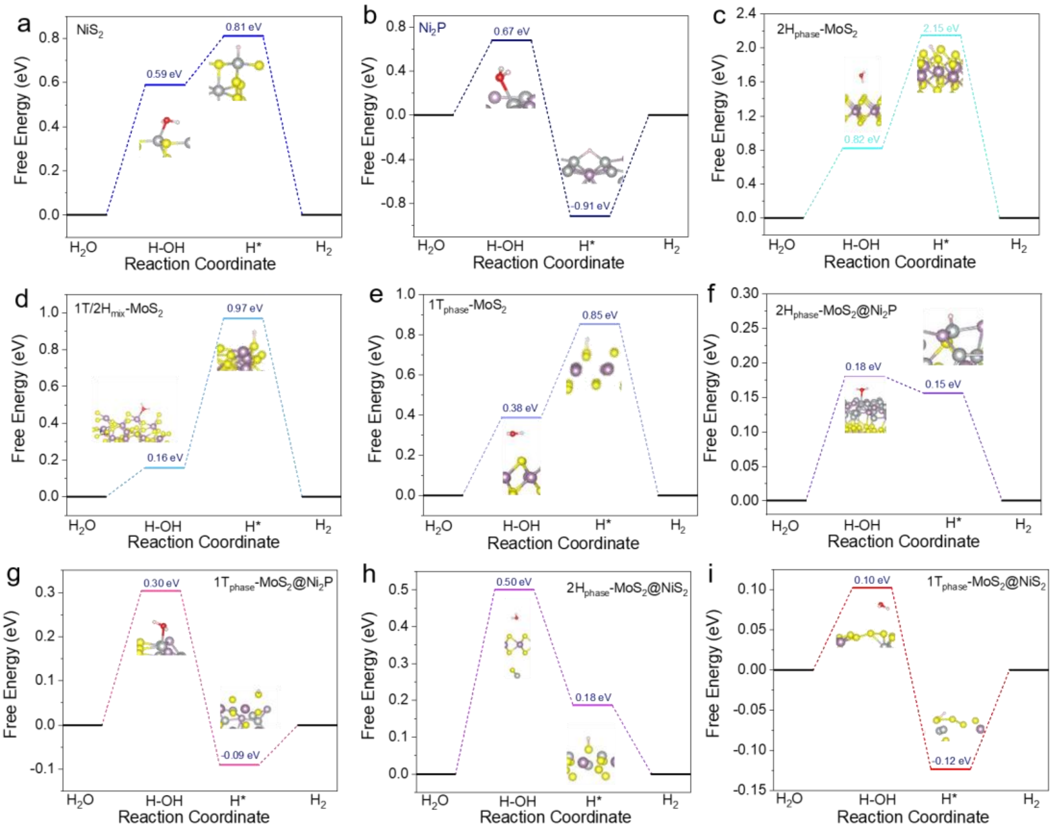

Supplementary Fig. 42. Free-energy diagrams for HER on the **a** $2H_{phase}$-MoS_2, **b** $1T_{phase}$-MoS_2, **c** pure Ni_2P, **d** pure NiS_2, **e** $1T/2H_{mix}$-MoS_2, **f** $2H_{phase}$-MoS_2@Ni_2P, **g** $2H_{phase}$-MoS_2@NiS_2, **h** $1T_{phase}$-MoS_2@NiS_2 and **i** $1T_{phase}$-MoS_2@Ni_2P interface edges.

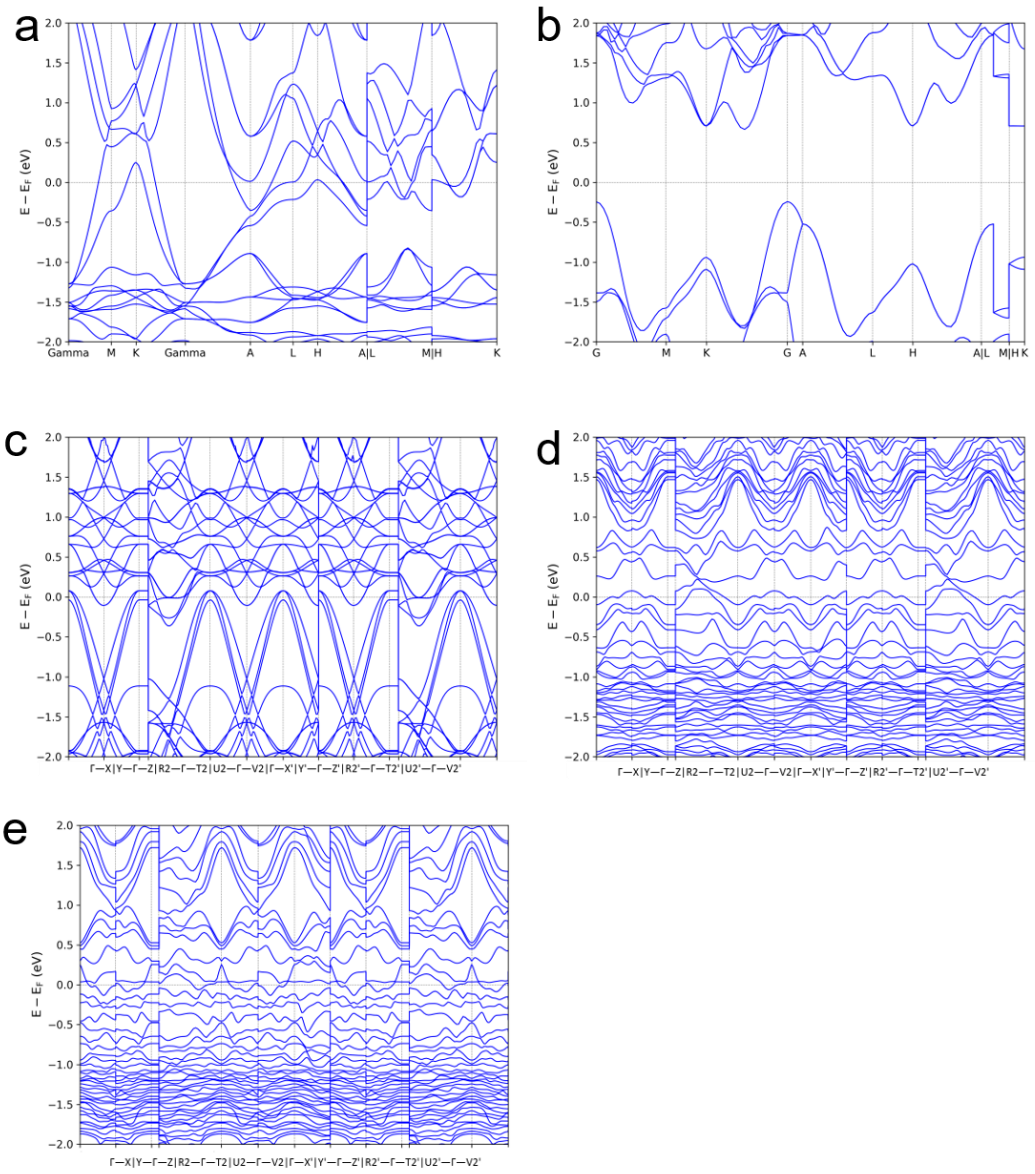

Supplementary Fig. 43. Band structure plots of **a** Ni_2P, **b** $2H_{phase}$-MoS_2, **c** $2H_{phase}$-MoS_2@Ni_2P, **d** $2H_{phase}$-MoS_2@NiS_2, and **e** $1T_{phase}$-MoS_2@Ni_2P.

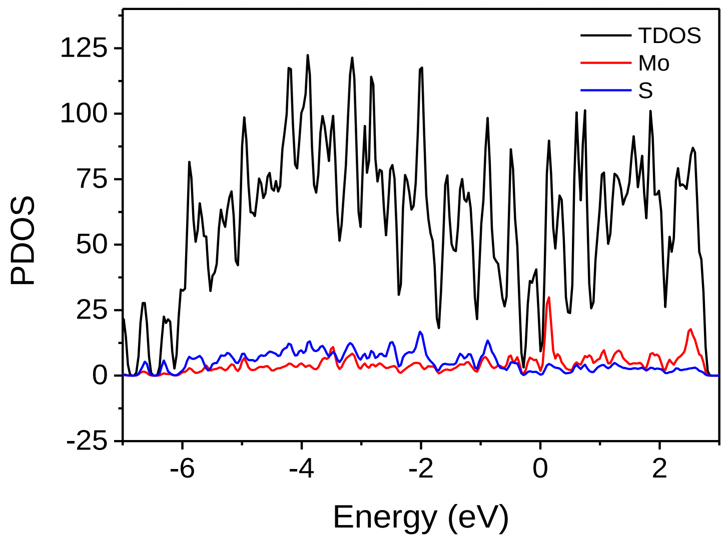

Supplementary Fig. 44. Density of states (DOS) plots of 1T/2H$_{mix}$-MoS_2.

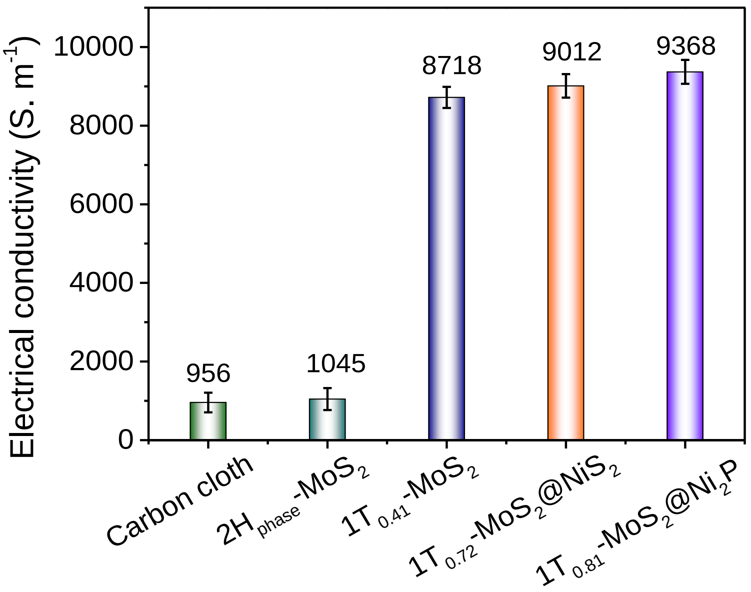

Supplementary Fig. 45. Electrical conductivities of $1T_{0.41}$-MoS_2, $1T_{0.72}$-MoS_2@NiS_2, $1T_{0.81}$-MoS_2@Ni_2P heterostructures compared with $2H_{phase}$-MoS_2 and carbon cloth. The electrical measurement with a ST2263 double-electric measurement digital four-probe tester. Error bars are the average of three measurements for each sample.

Supplementary Table 8. Calculated HER raw statistics of the six catalysts.

Catalysts	E_{DFT} (eV)	E_{slab} (eV)	E_{H+}	E_{ZPE-TS}	ΔG
**2H$_{phase}$-MoS$_2$**	**-624.53**	**-622.20**	**-3.37**	**0.20**	**0.97**
**1T$_{phase}$-MoS$_2$@Ni$_2$P**	**-225.82**	**-222.40**	**-3.37**	**0.21**	**-0.09**
**1T$_{phase}$-MoS$_2$@NiS$_2$**	**-315.42**	**-312.07**	**-3.37**	**0.21**	**-0.12**
**1T/2H$_{mix}$-MoS$_2$**	**-349.19**	**346.49**	**-3.37**	**0.19**	**0.85**
**2H$_{phase}$-MoS$_2$@Ni$_2$P**	**-545.12**	**541.88**	**-3.37**	**0.20**	**0.16**
**2H$_{phase}$-MoS$_2$@NiS$_2$**	**-177.96**	**-174.00**	**-3.37**	**0.14**	**0.19**
**1T$_{phase}$-MoS$_2$**	**-405.36**	**-403.98**	**-3.37**	**0.15**	**0.15**
**Ni$_2$P**	**-113.93**	**-109.78**	**-3.37**	**0.14**	**-0.91**
**NiS$_2$**	**-102.71**	**-102.71**	**-3.37**	**0.16**	**-0.81**

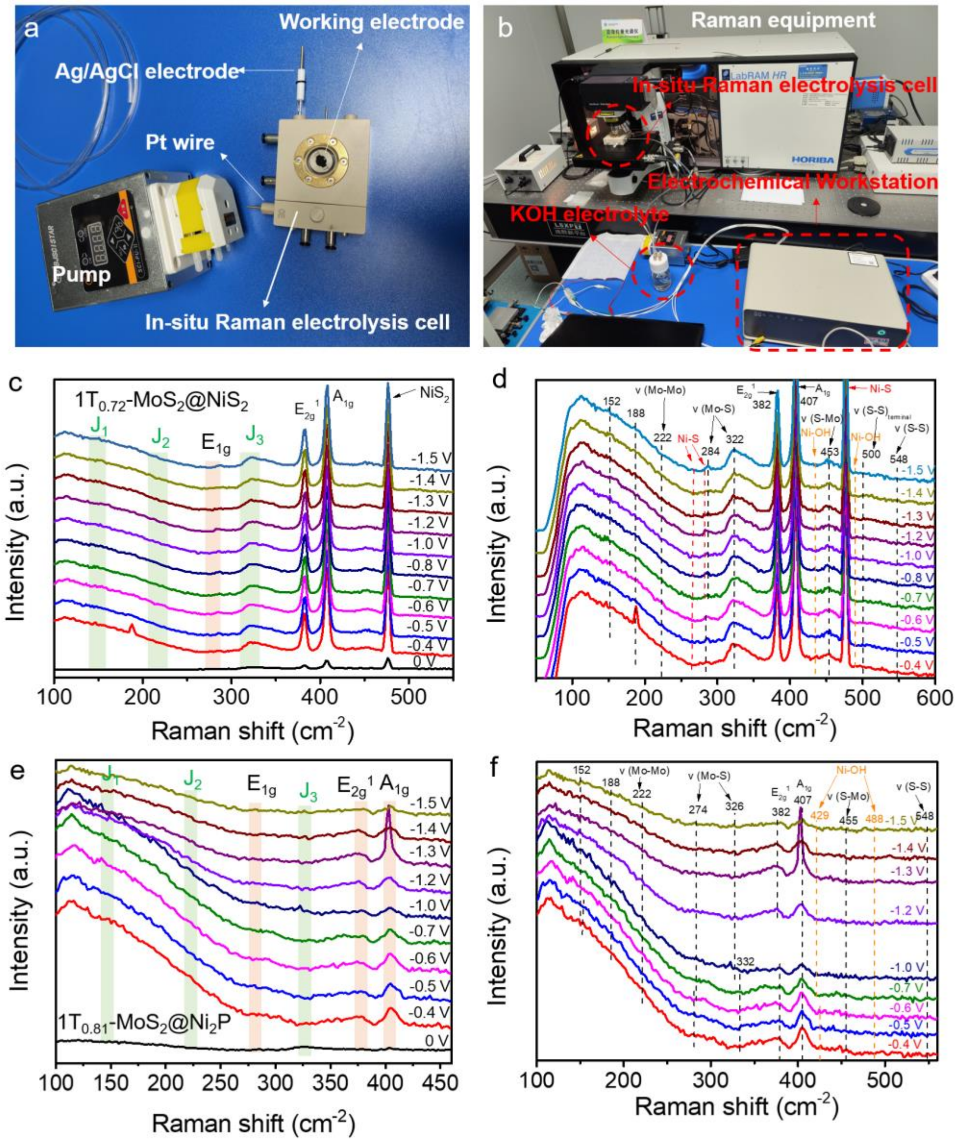

Supplementary Fig. 46. **a** In-situ Raman electrolysis cell. **b** In-situ electrochemical-Raman test system. **c-d** In-situ Raman spectra recorded of $1T_{0.72}$-MoS_2@NiS_2 and electrode, at different applied voltages from the 0 V to −1.5 V during electrocatalytic HER in 1.0 M KOH solution, scan rate 50 mV s^{-1}. **e-f** In-situ Raman spectra recorded of $1T_{0.81}$-MoS_2@Ni_2P electrode, at different applied voltages from the 0 V to −1.5 V during electrocatalytic HER in 1.0 M KOH solution, scan rate 50 mV s^{-1}.

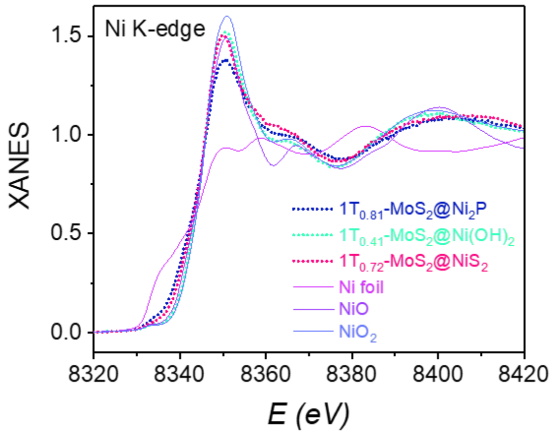

Supplementary Fig. 47. Ni *K*-edge XANES spectra of $1T_{0.81}$-MoS_2@Ni_2P, $1T_{0.72}$-MoS_2@NiS_2, $1T_{0.41}$-MoS_2@$Ni(OH)_2$, and standards Ni foil, NiO and NiO_2.

Supplementary Table 9. The fitted average oxidation states of Ni from XANES spectra on the fresh catalyst and after being used in the HER process at −0.04, −0.1 and −0.2 V vs RHE, respectively. ΔE: Ni *K*-edge position (eV), relative to Ni foil, Error = ± 0.2 eV; OS: Oxidation state (Linear fit: OS = 2.2222*ΔE-26.2222).

**$1T_{0.81}$ MoS_2@Ni_2P**	**White line peak**	**E_0**	**ΔE**	**OS = 2.2222*ΔE-26.2222**
Fresh	8350.5	8346.3	13.3	3.3
-0.04V	8350.9	8345.8	12.8	2.2
-0.1V	8350.2	8345.6	12.6	1.8
-0.2V	8350.5	8345.7	12.7	2.0
NiO_2	8351.1	8346.6	13.6	4
NiO	8350.8	8345.7	12.7	2.0
Ni foil		8333	0	0

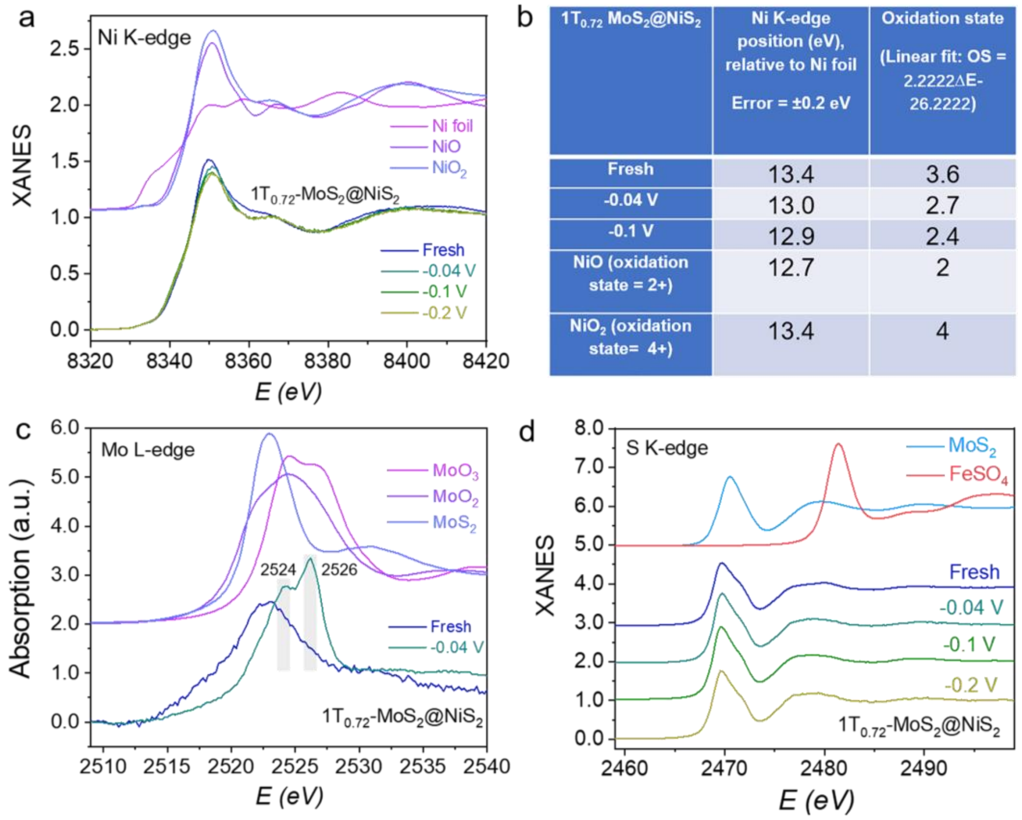

$1T_{0.72}$ $MoS_2@NiS_2$	Ni K-edge position (eV), relative to Ni foil<br>Error = ±0.2 eV	Oxidation state<br>(Linear fit: OS = 2.2222ΔE-26.2222)
Fresh	13.4	3.6
-0.04 V	13.0	2.7
-0.1 V	12.9	2.4
NiO (oxidation state = 2+)	12.7	2
NiO_2 (oxidation state= 4+)	13.4	4

Supplementary Fig. 48. a XANES spectra recorded at the Ni *K*-edge of fresh $1T_{0.72}$-$MoS_2@NiS_2$, and at different applied voltages from the −0.04 to − 0.2 V after electrocatalytic HER, and the XANES data of the reference standards of Ni foil, NiO, and NiO_2. **b** the fitted average oxidation states of Ni from XANES spectra on the fresh catalyst and after being used in the HER process at −0.04, and −0.1V vs RHE, respectively. **c** Mo *L*-edge XANES spectra of the fresh catalyst and after HER at the -0.04 V vs RHE condition for $1T_{0.72}$-$MoS_2@NiS_2$ catalyst, and the XANES data of the reference standards of MoS_2, MoO_2, and MoO_3. **d** S *K*-edge XANES spectra of $1T_{0.72}$-$MoS_2@NiS_2$ recorded on the fresh catalyst and at different applied voltages from the −0.04 to − 0.1 V after electrocatalytic HER.

a

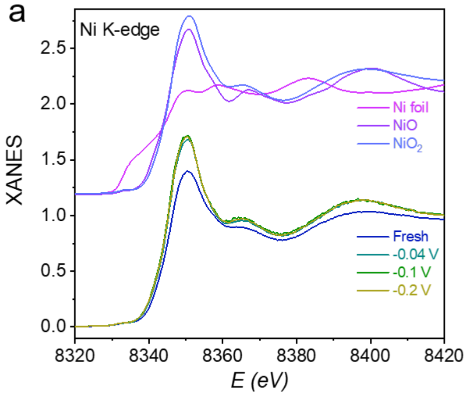

b

$1T_{0.41}$ MoS_2@Ni$(OH)_2$	Ni K-edge position (eV), relative to Ni foil<br>Error = ±0.2 eV	Oxidation state<br>(Linear fit: OS = 2.2222ΔE-26.2222)
Fresh	**13.0**	**2.7**
-0.2 V	**12.6**	**1.8**
NiO (oxidation state = 2+)	**12.7**	**2**
NiO_2 (oxidation state= 4+)	**13.6**	**4**

Supplementary Fig. 49. a XANES spectra recorded at the Ni *K*-edge of fresh $1T_{0.41}$-MoS_2@Ni$(OH)_2$, and at different applied voltages from the −0.04 to −0.2 V after electrocatalytic HER, and the XANES data of the reference standards of Ni foil, NiO, and NiO_2. **b** the fitted average oxidation states of Ni from XANES spectra on the fresh catalyst and after being used in the HER process at −0.04 V vs RHE, respectively.

References:

1. Benck, J. D. et al. Amorphous molybdenum sulfide catalysts for electrochemical hydrogen production: insights into the origin of their catalytic activity. ACS Catal. **2**, 1916-1923 (2012).

2. Zhai, P. et al. Engineering active sites on hierarchical transition bimetal oxides/sulfides heterostructure array enabling robust overall water splitting[J]. Nat. Commun. **11**, 5462 (2020).

3. Chakraborty, B. et al. Symmetry-dependent phonon renormalization in monolayer MoS_2 transistor[J]. Phys. Rev. B: Condens. Matter Mater. Phys. **85**, 161403 (2012).

4. Li, G. Q. et al. Activating MoS_2 for pH-Universal hydrogen evolution catalysis[J]. J. Am. Chem. Soc. **139**, 16194 –16200 (2017).

5. Nipane, A. et al. Few-layer MoS_2 p-type devices enabled by selective doping using low energy phosphorus implantation[J]. ACS Nano, **10**, 2128 – 2137 (2016).

6. Kresse, G. et al. Efficient iterative schemes for ab initio total-energy calculations using a plane-wave basis set. Comput. Mater. Sci. **6**, 15-50 (1996).

7. Hammer, B. et al. Improved adsorption energetics within density-functional theory using revised Perdew-Burke-Ernzerhof functionals. Phy. Rev. B **59**, 7413 (1999).

8. Wang, Q. et al.Oxygen vacancy-rich ultrathin sulfur-doped bismuth oxybromide nanosheet as a highly efficient visible-light responsive photocatalyst for environmental remediation. Chem. Eng. J. **360**, 838-847 (2019).

9. Dudarev, S. et al. First principles study of structure and properties of La- and Mn-modified $BiFeO_3$. Phy. Rev. B **57**, 1505 (1998).

10. Grimme, S. et al. Appropriate description of intermolecular interactions in the methane hydrates: An assessment of DFT methods. J. Comput. Chem. **27**, 1787-1799 (2006).

11. Chang, B. et al. Bimetallic NiMoN nanowires with a preferential reactive facet: an ultraefficient bifunctional electrocatalyst for overall water splitting. Chem. Sus. Chem. **11**, 3198–3207 (2018).

12. Zhang, J. et al. Synergistic interlayer and defect engineering in VS_2 nanosheets toward efficient electrocatalytic hydrogen evolution reaction. Small **14**, 1703098 (2018).

13. Lin, J. et al. Defect Rich Heterogeneous MoS_2/NiS_2 nanosheets electrocatalysts for efficient overall water splitting[J]. Adv. Sci. **6**, 1900246 (2019).

14. Zhai, Z. et al. Dimensional construction and morphological tuning of heterogeneous MoS_2/NiS electrocatalysts for efficient overall water splitting[J]. J. Mater. Chem. A, **6**, 9833-9838 (2018).

15. Yang, Y. et al. Hierarchical nano-assembly of $MoS_2/Co_9S_8/Ni_3S_2/Ni$ as a highly efficient electrocatalyst for overall water splitting in a wide pH range[J]. J. Am. Chem. Soc. **141**, 10417-10430 (2019).

16. Zhao, X. et al. Bifunctional electrocatalysts for overall water splitting from an iron/nickel-based bimetallic metal-organic framework/dicyandiamide composite[J]. Angew. Chem. Int. Ed. **57**, 8921-8926 (2018).

17. Hou, J. et al. Active sites intercalated ultrathin carbon sheath on nanowire arrays as integrated core–shell architecture: highly efficient and durable electrocatalysts for overall water splitting[J]. Small **13**, 1702018 (2017).

18. An, L. et al. Epitaxial heterogeneous interfaces on $N-NiMoO_4/NiS_2$ nanowires/nanosheets to boost hydrogen and oxygen production for overall water splitting[J]. Adv. Funt. Mater. **29**, 1805298 (2019).
